# Exploring presence practices: a study of unit managers in a selected Provincial Hospital in Free State Province

**DOI:** 10.1186/s12912-024-02023-7

**Published:** 2024-05-31

**Authors:** Bernardine Smith, Precious Chibuike Chukwuere, Leepile Alfred Sehularo

**Affiliations:** 1 NuMIQ Research Focus Area, Faculty of Health Sciences, North-West University, Potchefstroom, North West Province South Africa; 2 Lifestyle Diseases Research Focus Area, Faculty of Health Sciences, North-West University, Mafikeng, North West Province South Africa

**Keywords:** Caring, Nursing, Presence practices, Unit manager

## Abstract

**Background:**

Nursing presence depends on an individual’s belief system, truths, sensory experience, professional skills, and active listening. Thus, one may assume that presence occurs when nurses care for patients in a kind and compassionate way. This study aimed to explore and describe presence practices amongst unit managers in a selected provincial hospital in Free State Province.

**Methods:**

A qualitative research approach with an exploratory descriptive contextual research design was employed in this study. A purposive nonprobability sampling technique was utilised to select participants. Data were collected through semi-structured interviews and analysed using the six steps of thematic qualitative data analysis. The study’s trustworthiness was ensured through ascertaining credibility, dependability, confirmability, transferability, and authenticity. Approval to conduct the study was obtained from the North-West University Health Research Ethics Committee (NWU-HREC), DoH in the Free State Province, and the CEO (the gatekeeper) of the selected hospital.

**Results:**

Four themes were generated, namely, presence practices amongst unit managers in a selected provincial hospital in Free State, the impact of presence practices on hospital dynamics in a selected provincial hospital in Free State, unit managers’ practices of relational care and human connectedness in the unit, and the perceptions of unit managers on barriers to presence practices in a selected provincial hospital in Free State. Each of these themes presents categories and sub-categories. Unit managers actively foster supportive work cultures, effective management, human connectedness and relational care, and effective communication to yield team cohesion and positive impacts on patient care. Unit managers also display resilience and highlight the need for ongoing support from colleagues and top management.

**Conclusion:**

Unit managers exhibit diverse presence practices which emphasise their commitment through visibility and accessibility despite staff shortages and resource constraints.

**Supplementary Information:**

The online version contains supplementary material available at 10.1186/s12912-024-02023-7.

## Introduction

Nursing presence is described as an aspect of the art of nursing [1]. Nursing presence depends on an individual’s belief system, truths, sensory experience, professional skills, and active listening [2]. Thus, one may assume that presence occurs when nurses care for patients in a kind and compassionate way [3]. Furthermore, when true presence is practised by nurses, they identify what patients perceive as important through merely listening and observing a patient’s verbal and non-verbal behaviour [4]. Hence, it is important that effective communication occurs between the patient and the nurse to maintain and ensure a mutual relationship that is healthy and therapeutic [5]. Hansbrough and Georges describe nursing presence as occurring when nurses utilise practices to intentionally enter a positive and mutual relationship with their patients [6]. In essence, presence practices aim to facilitate human connectedness, relational care, and the attuning of care to the needs of patients [7, 8]. In healthcare, human connection or interactions nurture robust relationships and enhance nurse-patient engagement which could improve patient outcomes and experiences [1]. Therefore, presence practices are pivotal in ensuring quality nursing care.

Practising nursing presence can be beneficial to both nurses and patients. Patients may feel more comfortable in communicating their signs, symptoms, or concerns to nurses when there is a human connectedness and positive therapeutic relationship between the nurse and the patient [5]. This is essential as it aids the healing process of patients. Nurses also benefit both on a professional and a personal level when practising presence [9]. Moreover, the employer also benefits when presence is practised because there are less complaints from patients and their families and there is an improved organisational culture. Therefore, leaders should ensure team cohesion, listen to nurses, and provide the necessary information and a safe environment [10]. Similarly, the workplace culture would improve when all nurses feel enriched by practising presence whilst taking care of their patients in the hospital or care centres [11]. Nurses, who believe that their profession is perceived as positive, do a better job, engage more, listen more to patients, and in general achieve better results [12].

Unfortunately, presence is not always practised by nurses. Contributory factors were identified as possible causes that may lead to a lack of nurses practising presence. These include burnout and exhaustion, lack of professional nursing skills, and lack of resources [13]. Nurses, therefore, owe it to themselves to continuously develop professionally in order to keep up to date with new knowledge and skills that will enable them to provide better care to patients. To this end, society considers it vital, almost a prerequisite, that nurses possess high technical skills. This perpetuates the classical view of healthcare, nursing care, the nursing profession, and the recipient of care [14]. Thus, the creation of a nurturing environment, characterized by health, support, and compassion, is essential for enhancing the experiences of patients, their families, nursing staff, and unit managers [15]. Toxic leadership may impact negatively on nursing performance and adversely affect organisational culture [16]. Nurses also reported that the lack of respect from patients and the public demotivates them and has a major influence on their ability to practice presence [12].

Interestingly, the unit manager’s key role is to lead his or her subordinates in his or her perspective unit [17]. Therefore, a unit manager must be in possession of leadership skills, communication skills, and resilience in order to guide the team in the right direction [18]. Unit managers must be able to manage conflict situations and ensure team cohesion [19]. According to Beukes and Botha, employee engagement and staff performance will increase when there is effective communication which is essential to achieve quality patient care [20]. Mtise and Yako further mention that the unit manager’s roles encompass staff performance management, coaching and mentoring, budgeting, effective communication, human resource management, and equipment and stock management [21]. Abou Zeid and colleagues suggest that nursing leaders should be in possession of spiritual competencies which would aid their followers’ psychological capital as well [22]. Importantly, nursing leaders must acknowledge the capability of nurses to act in their positions and be the role models that they require [23].

However, there are insufficient published studies regarding presence practices amongst unit managers specifically in a selected provincial hospital in the Free State in South Africa. Shopo and Tau highlight the importance of an in-depth understanding of presence in South Africa [24]. Furthermore, Oukouomi-Noutchie heighten the concern over the lack of presence practices by South African nurses [25]. Thus, the focus of this study was to narrow the gap regarding this phenomenon. Owing to the vital roles of nursing unit managers and the importance of presence practices in nursing, the objective of this study was to unpack the presence practices amongst unit managers in a selected study area in the Free State in South Africa through a qualitative research approach. The study was founded on the core principles of presence practices, relational care, human connectedness, and the attuning of care [7, 8, 26].

## Methods

### Research design

A qualitative research approach and an exploratory descriptive contextual research design were followed [27–29]. The research approach and design were followed to explore, describe, and contextualize the perspectives of the unit managers on presence practice and how they practise relational care and human connectedness in the unit at a selected hospital in Free State Province. Therefore, owing to the vital roles of nursing unit managers and the importance of presence practices in nursing, the objective of this study was to unpack the presence practices among unit managers in a selected study area in the Free State in South Africa through a qualitative research approach.

### Study setting

There are four provincial hospitals in the Free State. The study was conducted in one of the main public provincial hospitals in Free State. This selected hospital was the ideal setting to conduct the research because it was a government institution with a variety of units that had large numbers of nursing staff. Additionally, the researcher believed that this selected hospital was ideal because of its high value in research and the fact that it is a referral hospital with an emergency unit.

### Population and sampling

A research population includes the entire group or units that constitute the research focus such as the unit managers working in a selected provincial hospital [28]. The population includes the unit managers working at the main provincial hospital in Free State. A non-probability purposive sampling technique was used in this study. The researcher purposefully selected this sample because the participants have all of the characteristics needed to take part in the study. The unit managers at the provincial hospital in Free State were the participants of the study. They were the most suitable candidates/participants to answer the research questions because of their roles. A total of 12 registered nurses participated in this study (10 female and 2 male participants). The sample size was determined through data saturation.

Inclusion and exclusion criteria were clearly defined for participant selection. Professional nurses registered with the South African Nursing Council (SANC) and employed as unit managers at the main provincial hospital in Free State for at least one year were invited to participate in the study. This criterion was chosen to ensure that participants held administrative and leadership roles, thus capable of providing in-depth insights into the research questions. Additionally, only unit managers who had been in their positions for a minimum of one full year were included. Interviews were conducted in English with all eligible participants. All other nursing staff categories, including registered nurses, enrolled nurses, and registered nursing assistants, were excluded from the study. Furthermore, the nursing manager (Matron) was also excluded.

An administrative officer assisted the researcher in distributing pamphlets at all strategic work areas within the selected hospital, including but not limited to nursing stations, waiting rooms, administrative offices, and staff break areas. The nursing manager encouraged the participants to partake in the study. The study participants who were interested in participating in the study contacted the researcher. A time was scheduled to obtain informed consent first. An independent person not directly involved in the research facilitated the signing of consent forms. The independent person was a master’s student from the primary researcher’s institution who had undertaken a course on research methodology and, hence was knowledgeable about facilitating the signing of informed consent forms. The researcher endeavored to educate the independent person about this study and the expected roles. The independent person was not directly involved in the study. The independent person ensured that participants understood their rights regarding participation in the study, gave them the opportunity to answer pressing questions regarding the study, and further allowed them to make informed decisions regarding participation in the study before signing the consent forms.

### Data collection method

Semi-structured interviews were used in this study to collect data from the study participants. Data collection commenced after the necessary ethical clearance was obtained from the relevant authorities. Semi-structured interviews were conducted with the participants who were interested in the study [29]. Data were collected from the participants who signed the consent form. Data were collected in a secured venue in the selected hospital which was clearly marked with ‘*Do not disturb*’. The participant was the only person allowed in the room with the interviewer when the data were collected. Data were collected using semi-structured interviews and in the English language. The interviews were conducted during working hours to avoid burdening the participants by requiring them to stay extra hours after work. Prior to each interview, the researcher introduced herself and confirmed that the participant had signed the consent form. During the semi-structured interviews, the researchers used a few communication techniques such as paraphrasing, probing, clarifying, and reflection. The interview sessions were audio-recorded with permission from the participants prior to recording. The interview sessions lasted for 45 min to one hour to enable the researcher to probe the questions for in-depth and broad data collection. The researcher took reflective field notes during the interview sessions whilst asking the research questions and probing questions and also maintained full control of the interview sessions.

The following research questions were addressed:

- What are the presence practices amongst unit managers in a selected provincial hospital in Free State?
- What are the perceptions of unit managers on presence practices in a selected provincial hospital in Free State?
- How does one practise relational care and human connectedness towards one’s subordinates in the unit?

### Data analysis

The data that were generated from the semi-structured interviews were analysed by the researcher and an independent co-coder using thematic analysis (TA). The analysts used ATLAS.ti to analyse the data [29]. Braun and Clarke’s six steps of thematic qualitative data analysis were used in the study [30]. These six steps are presented below:

- **Step one** – The researcher transcribed the data verbatim from the tape recorder, read through the data, and reflected on what the participants said. Data were shared with a co-coder who read through the data and reflected on the findings.
- **Step two – **Codes were generated from the collected data (phrases or categories were then coded) and were written down in a book to assist with the interpretation of the data [30]. The researcher and the co-coder independently conducted this step.
- **Step three** – The researcher searched for themes and categories which gave meaning to the data set [30]. This step was conducted independently by the researcher and the co-coder and then consensus was determined.
- **Step four** – All potential themes and categories were reviewed for quality purposes, coherence, and suitability [30].
- **Step five** – Themes and categories were named and defined by clearly stating the uniqueness of these themes and categories to provide structure for the analysis process [30].
- **Step six** – The researcher validated the generated themes and categories and reflected on them to ensure that they reflected the participants’ authentic responses [30].

### Trustworthiness

A qualitative researcher should be open and flexible at all times to ensure that the methods of data collection foster thoroughness and authenticity [28]. Polit and Beck mention that a framework for qualitative criteria was created by Lincoln and Guba in 1985 which suggested four criteria to ensure trustworthiness in qualitative research, namely: credibility, dependability, confirmability, and transferability [29, 31] In this study, trustworthiness was ensured through ascertaining the study’s credibility, dependability, confirmability, transferability, and authenticity. The researcher ensured that the research methods used in this study provided data that were analysed and interpreted truthfully [29]. The researcher ensured the portrayal of the truth whilst the collected data were interpreted. Credibility was ensured in this study through the application of techniques such as prolonged engagement with participants by spending time with the participants during data collection. This enabled them to consider and answer the research questions critically and thoroughly. Additionally, peer debriefing occurred through the consultation with peers to confirm the validity of the data. Finally, member checking occurred through summarising the participants’ answers during the interviews and confirming with them whether the information was correct. Dependability refers to the condition of the data over time as well as the reliability or stability of the data collected during the interviews [29]. This study ensured dependability as the findings of this research would remain the same if the study were to be repeated in the same context and with the same participants. In this study, dependability was ensured through means of an audit trail as detailed records were kept of the study from the beginning to the end of the study. The data of this research are representative of the information provided by the participants and not the researcher’s own inventions [29].

To ascertain the confirmability, one considers the objectivity, meaning, and relevance of the collected data in this research and confirms the findings. Objectivity was ensured by employing an independent co-coder. The study supervisors served as independent checkers throughout the study. The findings of this research are the true reflection of the participants’ own voices regarding their influences as unit managers on their subordinates. As noted by Lincoln and Guba, rich data that is descriptive in nature should be provided by the investigator [29, 31] Therefore, transferability was ensured by providing an in-depth description of the adopted research methods. Amidst the implementation and data collection of this research, authenticity was evident in the report on the findings by revealing a range of realities [29]. Authenticity in this research was ascertained through active listening to the participants during the data collection, which fostered the collection of in-depth and broad data that reflected the views of the participants.

## Findings

### Demographic characteristics of participants

Twelve registered nurses participated in this study, which included ten female participants and two male participants. The ages of the participants ranged between 44 and 59 years of age. These registered nurses are unit managers with years of experience in their current field which ranges between one and 20 years. Each of the direct quotes were supported by participant’s distinct characteristics which are participant number, gender, age and years of experience.

### Organisation of the themes

Four themes were extracted from the data analysis and are presented in Table 1 below.

**Table 1:** Themes, categories, and subcategories extracted from the thematic analysis

THEMES	CATEGORIES	SUB-CATEGORIES
1. Presence practices among unit managers in a selected provincial hospital in Free State	1.1. Leadership presence and accessibility	• Be available and accessible to patients and staff; • Be involved and visible; and • Establish a conducive workplace environment.
	1.2. Supportive work culture	● Supportive and helpful attitude towards staff; ● Acceptance of personal differences; and ● Personal interviews with staff and referral of nurses and patients who require interventions.
	1.3 Effective management and development	● Problem-solving and conflict management; ● Staff education and staff empowerment; ● Patient feedback with corrective interventions; ● Planning, time, and resource management; ● Clinical governance and participative leadership; and ● Encouragement and motivation of staff.
2. Impact of Presence Practices on Hospital Dynamics in a selected provincial hospital in Free State province	2.1 Positive impact on patient care and staff management	● Collaboration, harmony, and teamwork; ● Healing and calming effects on patients and their families; ● Patient satisfaction; ● Prevention of adverse events; and ● Effects on staff management.
	2.2 Challenges and negative outcomes	● Negative effects on patients’ and their families’ healing; ● Patient dissatisfaction; and ● Decline in quality patient care.
3. Unit managers’ practices of relational care and human connectedness in the unit	3.1 Practice of relational care	● Establish rapport and trusting relationships; ● Communication; ● Establish professional relationships; and ● Supportive practices.
	3.2 Practices to facilitate human connectedness	● Reaching out to others; ● Supportive actions; ● Empathy; and ● Team-building activities.
4. The perceptions of unit managers on barriers to presence practices in a selected provincial hospital in Free State	4.1 Managers and staff-related barriers to presence practices	● Staff compassion fatigue and burnout; ● Staff attitude-related barriers; and ● Staff members with personal disequilibrium.
	4.2 Work-related barriers to presence practices	● Insufficient resources; ● Staff shortages; ● Work overload and multiple expectations; and ● Inadequate support and acknowledgement.
	4.3 Managers coping with barriers to presence practices	● Coping through support from others; ● Emotion-focused coping (control stress and emotions); ● Problem-focused coping (improvisation); and ● Self-sacrifice.

#### Theme 1: Presence practices among unit managers in a selected provincial hospital in Free State

The above theme represents the presence practices among unit managers in the selected provincial hospital in the Free State. The theme demonstrates that participants practise presence in various ways. The participants’ responses provided insight into the discussion of presence practice in patient care. A few categories emerged, which are presented below and supported by direct quotes.

##### Category 1.1: Leadership presence and accessibility

Participants in the study underscored their commitment to practising presence through leadership presence and accessibility. This emphasis on leadership presence and accessibility illuminates the integral role that managerial figures play in fostering a culture of presence within healthcare settings. For instance, a few of the direct quotes from the participants revealed the following:

For, my patients as well. My patients usually I will start with them, because we are here for the patients. I’ll start with them in the morning after taking the report, we go bed by bed with them. I go with my staff, the matron is also there, myself going bed to bed seeing that every, you know, patients are being taking care of accordingly [Part 1, F-59, 2 yrs exp].

But for me definitely I think it is part of being here being visibly here, having an open door-policy,. But for the staff these days you have to be more present emotionally. You have to be seen physically as well, otherwise it is like oh, this matron is just in the office the whole day long [Part 2, F-51, 20 yrs exp].

A number of the participants further confirmed that they practise presence as managers through being there at all times and being visible in the unit for their staff. The participants’ direct quotes are presented below:

The presence means, okay on my side I understand it as I must be there all the time when my staff needs me, when there is something about my patient, I need to be there for that patient … [Part 12, F-54, 18 mnths exp].

What I understand when you said practice presence, according to me, I think I must be present on the patient at all times [Part 7, M-56, 1 year exp].

For, me presence means being visible with your team. Seeing that things or processes are running and everything. So, my presence being visible … [Part 6, F-54, 8 year exp].

Furthermore, certain participants explained how they, as unit managers, establish a conducive workplace environment for their subordinates. The participants’ responses are presented as the direct quotes below:

But if I can make the environment where you work, where a patient maybe sees his last minutes here, maybe before they die and I can make it positive, then I’ve done my job the day [Part 3, F-51, 6yrs exp].

… you don’t know how emotionally the person is heard but in the meantime on my side I have to start to make the environment of the theatre to be calm so that everybody is covered … [Part 12, F-54, 1 year.6 months exp].

##### Category 1.2: supportive work culture

Participants highlighted their ability to create a supportive work culture for both the subordinates and their patients by conducting personal interviews with staff and patients before referring them for specific assistance when needed. This supportive work culture also includes fostering positive and helpful attitudes towards staff members and recognising the diversity of their subordinates. In this instance, a few of the direct quotes from the participants revealed:

So I’m coming with her we are doing this thing like this and this and then if she, you see she is struggling you help. That’s why I said to you I carry them along because there are those you say nna cannot afford this I don’t think I can manage you come with them, that’s what I mean sitting with them, supporting them, supporting them that’s the main thing [Part 1, F-59, 2 yrs exp].

To say, but we, we are supporting you don’t resign or don’t drink. Because a lot of things came out yesterday, she’s not in a good state. Just to find out, she’s not drinking. We said OK we’ll take her out of that place, we are putting in another clinic, but whilst we’re doing that, we support you and motivate [Part 6, F-54, 8yrs exp].

The support that I give them is I’ll be there for them. They know even if I’m off during the week there is a problem, they called me and I come and help them. For there’s nothing I can do. There’s no way I can get other people somewhere else. So I’ll be there even if I’m home, they know they call me, matron come and help, I come and help [Part 9, F-50, 6yrs exp].

##### Category 1.3: effective management and development

Furthermore, in the participants’ attempts to explain their presence practices, it was established that they practise presence through effective management and development. As leaders, they verbalised the need for problem-solving and conflict management skills to ensure the smooth operation of the unit. The participants also highlighted how they motivate and encourage the subordinates to empower themselves through education and the provision of regular in-service training. In this instance, a few of the direct quotes from the participants revealed:

So, then they will come to me that I follow up with their doctors, so we solve the problems normally very quickly and there’s no further complaints on on issues like that, that small things normally [Part 3, F-51, 6yrs exp].

One of the responsibilities is to ensure that all the conflicts are resolved so that the key environment is very friendly to all of us. So, it’s very it’s very it’s very it’s very straining to try to keep to try to keep up with their fight, is very straining to to try to keep up to say let’s have a team because somebody have done something to and they take it personal [Part 6, F-54, 8yrs exp].

I will listen to the patient or the patient relative what is the problem, then I will go to the my staff and ask what was really happening just to investigate the matter. And then if we maybe the thing need to be solved, because at times it will be misunderstanding between the patient and the nurse, you know, miscommunication then we resolve it in my office like asking apologies and all those stuff [Part 7, M-56, 1 year exp].

Participants also mentioned the importance of clinical governance to ensure continuous improvement which contributes to high-quality nursing care and participative leadership in the workplace. Moreover, unit managers should plan their daily schedules actively whilst implementing effective time and resource management in order to ensure sufficient staff and resources to complete the daily activities in the units. Additionally, one of the participants mentioned how they respond to complaints and provide feedback to the patients, which included corrective interventions. A selection of the direct quotes from the participants are presented below:

… definitely just involving them will also increase your practice in presence in a leadership situation…actually participative management. So at least if you practice that type of management. I think people will also feel responsible for the unite and take ownership for the unit … [Part 2, F-51, 20yrs exp].

Now with clinical governance, we are accountable for, for whatever that we are doing to our patients, we are accountable [Part 4, F-44yrs, 2yrs exp].

And then do follow ups and sometimes maybe sometimes if the problem you know when you are working in an environment and then I’m working with you maybe I’ve got issues with you, like I’ll try to find out what is the problem, then I will separate them like in a shift maybe the other one will go to the other shift and the other one to the so to prevent the mis understanding [Part 8, F-50, 15yrs exp].

#### Theme 2: Impact of presence practices on hospital dynamics in a selected provincial hospital in Free State

The second theme delineates the impact of presence practices on the hospital dynamics in a selected provincial hospital in the Free State. According to the participants, practising presence can have both positive as well as negative effects on hospital dynamics. The categories which emerged are presented below and supported by direct quotes from the participants.

##### Category 2.1: positive impact on patient care and staff management

Participants communicated the positive impact on patient care and staff management when practising presence in a selected provincial hospital in the Free State. Participants explained how nursing presence improves patient satisfaction and positively affects staff management which contributes to harmony, teamwork, and collaboration in the team. The resulting environment has a calming and healing effect on patients and their families whilst preventing adverse events from occurring. The following direct quotes from the participants were selected to support this category:

Maybe the manager would say but we’ve got shortage in maternity, in ICU, wherever and then she place you there and she wasn’t actually for that. So, make the place friendly for her, ja. Obviously, she will be anxious, but I didn’t like this, though I’m a nurse, I didn’t like this. So I like making the work place friendly for them, ja by sitting around with them and then saying but this is not complicated [P1, F-59, 2yrs exp].

But if the environment is positive, I can’t fix the things at home for you? But if I can make the environment where you work, where a patient maybe see his last minutes here, maybe before they die and I can make it positive, then I I’ve done my job for the day neh. So, for me it’s very important that patients are happy and and that my staff is happy because if you if you can get that part, you will work harder. Everyone will give a extra more of themselves. If your environment is positive and what you call it? Conducive [P3, F-51, 6yrs exp].

If your personnel they are satisfied, happy and you always support them, even if you are having like, we having shortage of staff like in South Africa, even if there’s a shortage, they will always be willing to help, you see because they know that our matron is always there for us, so they will support you [P8, F-50, 15yrs exp].

All the support that you are giving them, they become relaxed, they they they tend to learn how to care for their babies and you won’t even struggle with milk production [P4, F-44, 2 yrs exp].

It’s all about like patient being like healed going out of the hospital being like healed you know going back to his family his or her family so that makes us very good as a team yes [P7, M-56, 1 year exp].

If my staff practice presence the advantages is that the stay of the patients in the hospital will be short and then one other thing, they would have too much burden of work if they do what they are supposed to do [P11, M-53, 1.7 yrs exp].

To make them feel relaxed that whichever way I’ll be taken into theatre, I will be operated that’s it, the most important thing is for our patients to know that is the most important thing, I mean the advantage of knowing is you are calming them, you are making them relaxed, you are making them believe that whichever way I’ll be done today [P12, F-54, 1.6 yrs exp].

##### Category 2.2: challenges and negative outcomes

Participants expressed their concerns regarding the challenges and negative outcomes associated with a lack of presence practice amongst unit managers and staff in a selected provincial hospital in the Free State. These negative outcomes and challenges mentioned by the participants may lead to patient dissatisfaction and a decline in the quality of nursing care. Subsequently, the healing process of the patients and their families could be affected negatively. These concerns are expressed in a selection of direct quotes from the participants:

So many things can go wrong, so many things can go wrong if really they are not happy, they are not coming to work because who will nurse the patients you know [P1, F-59, 2yrs exp].

There will be more death, the death rate will be higher. So, mortality will increase as it is at the moment we don’t have a high care so, my ward is also a high care. So, if we are not present, we will really have a bad mortality rate here and it will really increase [P4, F-44, 2yrs exp].

Yeah, the mothers well if we don’t practice presence. They are also not supported. They don’t support their babies and they feel frustrated. They don’t know who to talk to. Therefore, you’ll be having this, this, I don’t want to say bitter mothers that you will behave in these mothers that are not active in their babies care that are not participating whenever you need them to participate, and that also indirectly affects the baby. Because they need their moms to be there for them [P4, F-44, 2yrs exp].

One patient will complicate, two you won’t identify the problems on time [P5, F-48, 2yrs exp].

The disadvantage, the anxiety with our patients they are lying there not knowing. I’m being collected from my ward but I’m lying here, you understand, so it’s the anxiety that I think that can go through [P12, F-54, 1.6 yrs exp].

I would think, depending on whether your patient is a minor or not. Or even a geriatric patient, or a patient cannot speak for him or herself. They will also be feeling negative about the very institution where the patient is being nursed. They might feel unhappy with the care that the patient is getting [P2, F-51, 20 yrs exp].

Furthermore, some of the participants observed that the image of the of the hospital could be affected negatively when unit managers do not practise presence. Simultaneously, it could affect negatively the patients’ and their families’ healing. It is important to acknowledge the public or community trust in the nursing profession and the crucial roles of the unit managers in managing the units. For instance, participants maintained the following:

Even our image as a hospital, you know will be at the bad side you know to the community, they won’t even trust our hospital, yes [P7, M-56, 1 year exp].

If I don’t practice presence the disadvantages is that there will be no smooth running of the department, there’s gonna be chaotic, and then its gonna, there’s gonna be lot of complaints that the patients are not cared for. And then even that will lead to the long stay of the patients [P11, M-53, 1.7 yrs exp].

The patient will die, definitely the patient will die. That is the only thing the patient will suffer, there’s nothing. If you don’t do anything the best to the patient that you are here for, who’s suffering, that helpless patient, if you don’t do anything if you don’t do wound dressing on daily basis the patient will end up being septic, if you don’t clean the wound of the patient the patient will be septic [P10, F-55, 3 yrs exp].

#### Theme 3: unit managers’ practices of relational care and human connectedness in the unit

The third theme demonstrates how unit managers practise relational care and human connectedness in the units with their staff and their patients. This theme explores the different strategies employed by these unit managers to attune relational care and human connectedness in their units. A few categories emerged which are presented below and supported by direct quotes from the participants.

##### Category 3.1: unit managers’ practices of relational care

The ability of unit managers to practise relational care to patients and staff was expressed by the participants. This category recognises the participants’ use of supportive practices towards staff members as well as patients and their families. Communication was highlighted as integral to practising presence whilst establishing trustworthy and professional relationships as well as rapport in the work environment. The direct quotes from the participants reveal this category:

The relationship, the relation I’m building with my staff, listen to their concerns and then if maybe I see they are not on the line I discipline them and then I try to motivate them to do what they are supposed to do [P11, M-53, 1.7 yrs exp].

So, I’m always around and helping all over the hospital, and that’s also something that I say to them, remember it don’t help we keep our knowledge for ourselves, so even if we need to go to other departments and share our knowledge, use it like that [P3, F-51, 6yrs exp].

Like when you are a manager mos you must communicate with your staff. So, like every morning when during report taking I’m there with them so that I can know what is going on in the unit and then to understand if there is any challenges like staff shortages or equipment [P8, F-50, 15yrs exp].

Supporting everybody. Where they need support, knowing your staff, seeing when they are having an off day or not feeling well, asking them about them [P2, F-51, 20 yrs exp].

##### Category 3.2: practices to facilitate human connectedness

In this category, participants verbalised their practices to facilitate human connectedness by reaching out to others and engaging the team in team building activities. They described the importance of unit managers having empathy with staff and providing the others with the necessary support. Direct quotes from the participants reveal their perceptions:

Well, sometimes, for instance, when they are busy and there are a few people, I will assist but if I can’t assist, I will do just give them a little something like for instance a cappuccino sachet that they can enjoy on teatime or something like that and they do appreciate those little gestures [P2, F-51, 20 yrs exp].

If somebody have done and everything correctly I will buy some a cup just to say you have done well good for this, you understand. Others are depending being depressed neh because of some other social problem that I have but what I usually do I will let the staff to contribute whatever they have to buy something, it can be a slipper or whatever or a present or whatever, just to say [P12, F-54, 1.6 yrs exp].

Ja, is to my staff and we do some things in the ward just for team building, mustn’t be work, work, work all the time. So sometimes I’m there doing somethings cheering us up [P1, F-59, 2yrs exp].

Human connectedness to my staff, sometimes we hold the small, the small parties together and go out to be together in our free time so that we can have time to know each other well even outside our workplace [P11, M-53, 1.7 yrs exp].

#### Theme 4: The perceptions of unit managers on barriers to presence practices in a selected provincial hospital in Free State

The final theme depicts the perceptions of the participants on barriers to presence practices in a selected provincial hospital in the Free State. This theme also identifies different types of barriers that could hinder the practising of presence. A few categories emerged which are presented below and supported by direct quotes from the participants.

##### Category 4.1: managers and staff-related barriers to presence practices

Participants identified the managers as well as staff-related barriers that may impact presence practices. They highlighted a few factors such as fatigue and burnout as barriers to presence practice. Participants also highlighted other contributing factors, such as staff attitude as well as staff members with personal disequilibrium, as barriers to presence practices. The following direct quotes from the participants address this category:

The unit manager, operational manager. Name it what you want to is actually emotionally and psychologically drained she cannot support the rest of the people, neither the patients, neither the staff, neither the family members, nobody [P2, F-51, 20 yrs exp].

They don’t want to be confronted, you know, they feel that if you are wanting things to be done as they’re supposed to be done, you’re on them, on their case. So, that’s what I’m saying people are just on the edge. If you say a thing, no matter how in what context they’re just on fire day [P6, F-54, 8yrs exp].

The challenges sometimes the nursing staff others especially at night *mos* they would be rude maybe they are tired so like when the patient is asking for something they would be like rude to the patient or ignorant [P8, F-50, 15 yrs exp].

Others will be abusing substances. There are those that are abusing substances. There are those that are that are going through divorce [P4, F-44, 2 yrs exp].

##### Category 4.2: work-related barriers to presence practices

Barriers that are work-related were identified by the participants. These include, but are not limited to, insufficient resources, work overload, multiple expectations, staff shortages, inadequate support, and the lack of acknowledgement. A few direct quotes from the participants may elaborate:

It’s not always to say that we can physically change the situation or. Say for instance, you don’t have a certain kind of consumable. Doctors, sisters, everybody will get frustrated and highly frustrated. And then you have to be the sound board for that [P2, F-51, 20 yrs exp].

Shortage of resources, cos sometimes they are the one next to the patient. If maybe there is no resources, they become frustrated. So, they have to leave the patient in the ICU to go and ask around you know, so those things like frustrate them and then their interpersonal relationship also like people they are not the same so those things [P4, F-44, 2yrs exp].

Presently, it’s so challenging because like, some of the things that, I can’t even, not a problem, for example there’s a shortage of staff, there’s not enough personnel as I indicated, shortage of what you call it, equipment and also absenteeism also affect the things that we manage, some of the things that affects me, yes [P7, M-56, 1 year exp].

Sometimes we are so exhausted even the nurses I see sometimes really they are so exhausted but we keep on working, we keep on working because even if there is no someone, I have to call them come to do overtime, come to help us [P9, F-50, 6 yrs exp].

##### Category 4.3: managers coping with barriers to presence practices

Participants shared their coping mechanisms for the barriers to presence practice. Evidently, as managers, they are faced with several challenges. Consequently, they are forced to resort to various courses of action in. order to cope with these barriers which could affect practising presence. Some of the participants indicated that they rely on others for support. Additionally, they have to result to improvisation, self-sacrifice, emotion-focused coping, and problem-focused coping. This is illustrated by several direct quotes from the participants:

…and I must say that, sometimes you get more support than other times in the sense of not only just nursing management, but also your doctors that actually works with you or the ones that’s in charge of your units [P2, F-51, 20 yrs exp].

Even me, I need sometimes to just go and rebrief with my manager or with one of the doctors, you know, because we need because of the death rate [P3, F-51, 6yrs exp].

I do get support from the two ladies that I’ve mentioned my assistant manager and my nurse manager, recently just they’ve supported me fully. I don’t want to lie [P5, F-48, 2 yrs exp].

Fortunately with me I’m a very, very, calm person, even if I see that I can’t manage, I’m a very, very, calm person, I wouldn’t even show you that now I’m so much overwhelmed. I’ll take them one by one, one by one until I finish what I’m supposed to be doing for that day [P12, F-54, 1.6 yrs exp].

So, we’ll be improvising, improvising and then at end when the families came here then they gonna log the complaints? [P11, M-53, 1.7 yrs exp].

## Discussion

Presence practice exists amongst unit managers, but it is practised differently. In this study, the unit managers practise presence merely by being visible, available, and accessible to the staff and the patients. Mohammadipour et al. explain that “nursing presence” refers to being there for patients and that it is the essence of interaction between nurses and patients [32]. Unit managers attempt to provide the staff with a conducive working environment to the best of their ability. The supportive work cultures also were evident because the unit managers conducted personal interviews with the staff and patients regarding problems that were identified. Subsequently, the unit managers referred them to the relevant persons for assistance when necessary. Moreover, the unit managers also attempted to manage their staff effectively through participative leadership, clinical governance, problem-solving, and conflict management. These are critical skills and knowledge that are essential traits for unit managers to facilitate evidence-based nursing and effective management of their units. In addition, unit managers encourage and motivate their staff by providing staff development opportunities and empowering them to grow professionally. Even though not all staff members may be interested in furthering their academic studies, the unit managers frequently provide in-service training or on-the-job training which similarly empower their staff in the units. Lovinck et al. refer to “a leader who fosters and reinforces changes for improvement” [11].

To ascertain the perceptions of the unit managers on presence practice, the study identified positive impacts as well as negative impacts on hospital dynamics. When nurses practise presence, it prevents adverse events whilst having a healing and calming effect on patients and their families. When unit managers practise presence, it has a positive impact on staff management. Staff work together in harmony when there is collaboration and team cohesion. Additionally, Du Plessis highlights the importance of staff supervision and direction to improve staff work performance, motivation, and morale [33]. Consequently, the unit managers should receive training to improve their competencies and skills [17]. Despite the positive impact, challenges and negative outcomes were verbalised when unit managers did not practise presence in their units. This could have a negative effect on the patients’ healing process, lead to patient and family dissatisfaction, and hamper the quality of patient care. Paturra et al. state that “nurse leaders both indirectly and directly influence their subordinates’ performance” which could impact the quality of nursing care [17].

Additionally, unit managers attempt to practise relational care in their units. They establish rapport, they attempt to build trusting relationships through effective communication, and establish professional relationships with others by means of supportive practices. Shandu emphasises the importance of two-way communication to assist in strategies that enhance hospital dynamics [34]. Simultaneously, unit managers attempt to facilitate human connectedness by showing empathy, reaching out to others, supporting others, and even planning extra curriculum activities such as teambuilding activities after hours. Du Plessis further adds that “presence is about connecting to the other and attuning to their needs” [33]. An added benefit to an institution could be if a leader is also a spiritual leader who can revolutionise the nursing environments and provide adequate guidance and support [22].

Finally, the study revealed the participants’ perceptions of the barriers to presence practices in a selected provincial hospital in Free State. The study revealed that staff-related barriers to presence practices were issues such as staff attitude, staff with personal issues, and staff burnout or fatigue. Blackman et al. allude to the reasons for nursing care to be neglected as a lack of adequate staffing and inadequate resources which could result in staff burnout and attitude issues [35]. A few other barriers to presence practice that were highlighted are insufficient resources and work overload due to a shortage of staff. In essence, these were factors outside of the unit manager’s control. Interestingly, a study conducted by Blackman et al. states that staff shortage is not the primary cause of missed patient care, even though it does contribute to it [35]. Sarkhosh et al. also emphasise in their study how resource constraints impact on patient safety [36]. At times, the unit manager lacks the provision of support and fails to acknowledge staff performance. These unit managers are also under tremendous stress. At times, they need to find ways to cope with certain barriers to practise presence. Some of the participants expressed the lack of support whilst others indicated that they were fully supported from top management and their fellow colleagues. According to Quenon et al., top managers are under immense pressure as they are held responsible for positive and negative results as well as ensuring that their facilities run smoothly [10].

## Conclusion

In conclusion, this study unveils the perceptions and practices of unit managers regarding presence in a Free State hospital. Unit managers exhibit diverse presence practices by emphasising their commitment to visibility despite resource constraints and staff shortages. They actively foster supportive work cultures and effective management in order to have positive impacts on patient care and team cohesion. Conversely, challenges arise when managers lack presence, which affects patient healing and nursing care quality. The study underscores the importance of relational care, effective communication, and coping mechanisms. Unit managers display resilience by navigating obstacles and emphasising the need for ongoing support from top management and colleagues.

### Supplementary Information

Supplementary Material 1.

## Data Availability

To access the data in this study, kindly contact the corresponding author. The interview (data) used in this study was developed for this study and has previously not been published elsewhere. The data was uploaded as a supplementary file.

## Abbreviations

CAQDAS: Computer-Assisted Qualitative Data Analysis Software
NWU: North-West University
SANBS: South African National Blood Services
SANC: South African Nursing Council
