# Supplementary material for: Exploring presence practices: a study of unit managers in a selected Provincial Hospital in Free State Province
Source: BMC Nurs. 2024 May 31;23:367. doi: 10.1186/s12912-024-02023-7 (PMC11140984; doi:10.1186/s12912-024-02023-7)
Supplement: Supplementary file 1 — Supplementary Material 1. [file 12912_2024_2023_MOESM1_ESM.docx]

**Topic: What are the presence practices amongst unit managers in the Free-State Province:**

- What are the presence practices amongst unit managers?
- Define presence?
- How do you as a unit manager practices presence in your unit?
- How do you as a manager practice relational care and human connectedness towards your subordinates?
- How does your leadership role affect you as a unit manager to practice presence in the unit?
- What are the advantages of nurses and unit managers practicing presence?
- What are the disadvantages of nurses and unit managers not practice presence?

**Participant 1:**

Good day Matron, my name is Bernardine Smith, I’m a master’s student at the NWU, nursing +

sciences.

Participant 1: You are welcome.

Thank you very much. I really appreciate it taking this time off to answer a few questions. Like I mentioned there is no right or wrong just your perspective. It will be about 30 min minimum, could be a little bit more depending on how our interview goes. You don’t mind if I record it.

Participant 1: No I don’t mind, I don’t mind at all.

If I do not, if there might be maybe follow up questions is it fine if I maybe contact you on a later stage just to ask a few follow-up questions.

Participant 1: Yes, it would be definitely fine.

Thank you very much. Thank you so much. So let’s start, my research is about presence practice amongst the unit managers, okay in this hospital, okay. So what can you tell me about what is your understanding about presence practice of the unit manager.

Participant 1: Of the unit manager? The unit managers, if I may tell you about it here at this hospital, it’s a lot, especially being an operational manager, a unit manager because you are not being a manager in your office only. It goes to an extend of helping the patients, supporting your staff. It carries a lot but ahh. What we have studied it’s all about the management. It’s just how you utilize your time.

So how does your role, how does your leadership role influence your position as a unit manager, when it comes to presence practice.

Participant 1: Is my, can you phrase it well because I don’t want to answer it as if I’, in a classroom.

Okay.

Participant 1: I want to be broad.

How does leadership roles influence unit managers practicing presence.

Participant 1: You know what as a leader neh you lead people, but how do you lead them, you must carry them along. You must not just dictate. You know there’s different styles of leadership mos. That’s why I said you mustn’t be just dictating leader, you must just take them along. Be that leader who should lead your people but at the same time seating with them around communicating that, in a way you are leading them but you just also want to know how are their feelings, how do they know their skills about this. You know you don’t just dictate to people because if you do that they don’t seems to be interested. You must be friendly to them though its a norm that as a leader you must do this and this but coming in a way that is positive to them that is friendly to them. That’s why I said you take them along as a leader and that said okay this is what you suppose to do and if it’s suppose to be done, this is how its been done, you must also teach them because its useless to just through somethings to them and just leave them like this because they become, If they don’t know it means they won’t do it. But if you also show interest in them, they said okay this is how we do it and then you see okay this person is also interested in this and then you go along with them. But leadership goes a long way a long way that’s why I set your terms with them must be that friendly one but remember you mustn’t be that one with goes like if they tell you do this matron no you don’t want to do this then you leave it. You must tell them it must be done but let’s find a way how we can do it because at the long run it must be done. Because there’s this thing CPD points. I understand the other provinces is being done so we starting here. So I’m gonna start it with them something very, its not difficult but new and I see its gonna be a very its gonna be very disturbing to them because it has some of them, they don’t like reading so it means you must show them how important to read to update your brain update yourself your skills. So that’s why I said if theres something new got to be brilliant how to come to them otherwise you gonna if you just gonna throw it to them be dictative then you gonna have a problem and most of the things is because of shortage you know. Some people are willing to do things but shortage and also encourage them to read go to school, education goes a far way because if you don’t then you are talking to people who are clueless and skillsless you know you encourage them, mm, thank you.

Researcher: You mentioned that you have to carry them a long can you give me more details on how you go about to carry them along.

Participant 1: For instance this one, this point it means every time when I’ve got something new, I go to them and say you know this is what we supposed to do, in other words I sit with them carrying a long sitting. I don’t like throwing something to somebody, sitting with them and then okay lets do this together and see if its how easy or how difficult it is, so I’m coming with her we are doing this thing like this and this and then if she, you see she is struggling you help. That’s why I said to you I carry them a long because there are those you say nna cannot afford this I don’t think I can manage you come with them, that’s what I mean sitting with them, supporting them, supporting them that’s the main thing.

Researcher: Thank you ma’am. You also mentioned about encourage, how do you encourage them.

Participant 1: How do I encourage them. Mm, ja you know people mos are not interested most of the things now so you must encourage people, you must encourage them like I said mostly with me I encourage people by doing things with them, by doing things with them lets do and see how we can come far with you with this so you see if this is not so impossible jah, that’s how I mean by encouraging and encourage goes also goes a long way I know it goes a long way ee like talking to somebody now and then, you mustn’t it mustn’t be only once, I said this must be done okay tomorrow if today maybe this is her mind tomorrow maybe she would be somebody different. I think so.

Researcher: Okay thank you very much. What do you understand by presence practicing.

Participant 1: Presence practicing, ey in nursing, its I don’t know, I don’t want to say.

Researcher: Not what the reality is but according to you, what do you think should be practicing presence.

Participant 1: you know what, I think its not much, the practicing is still fine, the practicing is still fine I think its because people are over loaded so they think this is impossible but if people, staff can be maintained and people gives us staff then thing wouldn’t be so impossible. Because now nowadays its like hey you are allocating us for so many things so we cannot grab this we cannot do this you know at once, give us chance to do this but if you allocate this, this one wena you are doing this and you for me and you must do this, I think then one one has got a chance to learn then you swab the allocations, then somebody can see at the long run every one knows what is suppose to be in the unit. Because now, I think at the schools of learning of educations they still being given though the curriculum changes, they still being giving the opportunity to practice whatever they must practice in the ward. The thing is now the skills lack because things are moving fast, things are moving fast we expected them to do this know this, know this and when it comes to practice in the ntho it becomes a bit difficult for them but the old people as I can see, the old staff, I can work better with them than the old the new ones. Because they’ve got patience, they,ve got patience but okay even though I don’t do this today I think tomorrow I will do it neh and the the practice, they don’t jump jump they do exactly. It can just take time for them but at the end they would do whatever they are supposed to do but the new ones they don’t have patience, they don’t have patience. So, the practice is a little bit for nowadays impossible for the new ones I see it as an older midwife, its impossible because they don’t have patience but with us its still there because unfortunately the routine should be like that and the practice should be like that its just it changes with people, its people who changes it actually.

Researcher: you said this are moving fast, can you give us a few examples of what are those things that you are mentioning? You said things are moving fast.

Participant 1: mmm, mmm, like like eee, like I said as I compared the curriculums. In our olden curriculum, mos it would be like we also had a four year program but for it would be introductory year, the following year you would be doing this psychiatric or midwifery or what now this intertwined, they just mix, you’ve got to grab this and the the lecturer is done with you. Then this one is done with you so that’s why I think things moves fast and ee when it comes to practice there are new things mos that government is expecting us to do this expecting us to do this so some people they cannot cope with it. That’s why I said and like this CPD points it’s expected us to know you know some of the things and okay the problem is the training, the training, like I said you must go along with somebody. If you expect somebody to do this than you must give her prac, eee learning opportunity and training neh. So nowadays things are just thrown to us, you’ve got to grab them, know how to do it, so that’s why I said things are going fast, so it’s a load to us, load to us but as I said can tell you with us the older staff its much better. Ja we can comprehend better but the new ones they are the ones who know technology much better but I don’t know why they are luggish to do the things in the ward.

Researcher: when you say new ones are talking about students, are talking about newly employed staff or what are you actually talking about.

Participant 1: The students they do come and go but I cannot count on them, but the new ones, the newly employed, the newly employed ones because they will come, they will come then are we still there neh, the will come, you orientate them, you are giving them the things to do in the ward and then hey you can see whether this one in more enthusiastic then the other. Then the others they just dragging their feet, so aaa I think its like comparing the newly employed ones with the ntho those who are exactly from the college and now they just give you 2, 3 years and then they resign, they dpnt stay long. I think the impact of ward is not not, not them ja. They cannot cope with the work load, workload.

Researcher: As a unit manager what can you do to assist them coping with the work load, is there anything that you can do from your side as a unit manager.

Participant 1: We do, I do, you know will be taking them for several in service trainings.

Researcher: in-service trainings

Participant 1: Ja, how do I, you said how do I encourage them to stay or to cope. Most of them do just maybe they just anxious. To alleviate that anxiety, usually I sent them for in-service training besides me talking to them , taking to them every day, eyy you know after the eyy coming on duty the roll call. I, I like to sit with them, today this is what we going to do, or for this month, I’m allocating for you just to get use to the ward, or this is what we are going to do, besides this im sending them for the in-service trainings, in-service trainings, where they get people to talk to them when they can see how things are being done somewhere so that they can say no but we are not in a strange place this we can do, so if you should come with them like that its getting better. Like just throwing things at them, allocating them, you know, not seeing them as newly employed thar needs support. Besides what they are supposed to do in the ward, support, ja they must, you must encourage them to tell you if they are having problems so that you can talk how you can e e e you know come to that extend that you how to solve their problems, yeh because some of them they are staying far away, how can I, because you applied here, you know that this far away from your home but maybe in your mind you had this, so she must tell you what she had in mind, so maybe after the allocation, the orientation, you say okay, now that you are getting used to the ward, I can allocate you maybe for night duty so you get more off duties and then you can get more offs so you can take care, visit people or if she’s locally, some of them, mm, they’ve got babies mos a family, so you mustn’t. They know the hours but just make sure that is she happy with the hours which hours will be more suitable for her. That’s my support to them.

Researcher: Mmm, last question on that topic, is mmm you said that they become anxious. What are the reasons that they are suffering from anxiety? What makes them axious?

Participant 1: you know, I think in the first place, they will be applying to the hospital and then though they given their choice of where they should be allocated, maybe the manager would say but we’ve got shortage in maternity, in ICU, wherever and then she place you there and she wasn’t actually for that. So, make the place friendly for her, ja. Obviously she will be anxious, but I didn’t like this, though I’m a nurse, I didn’t like this. So I like making the work place friendly for them, ja by sitting around with them and then saying but this is not complicated. So I like besides the what is this, my staff in the ward, like senior sisters, senior professional nurses, I like orientating them myself so that I cannot blame somebody and say that you didn’t do this and this. So usually I go step by step and then the others will concentrate maybe on the routine but some staff I like to sit with them and then talk to them just to make them comfortable in the ward ja.

Researcher: okay thank you, so as a unit manager, how do you practice presence in your unit

Participant 1: My presence,

Researcher: How do you practice presence, being there for them.

Participant 1: For them, aa

Researcher: in your unit

Participant 1: For everybody now,

Researcher: for your patients as well

Participant 1: For my patients as well. My patients usually I will start with them, because we are here for the patients. Ill start with them in the morning after taking the report, we go bed by bed with them. I go with my staff, the matron is also there, myself going bed to bed seeing that every, you know, patients are being taking care off accordingly. so I go on and on and then after that I will go again to them because maybe yesterday they were newly admitted patients ans then I said I’m matron (name with held), and then if you are having anything just come to me or to the sister but you don’t sleep with a problem you just come to me. But the others I know they are shy then we’ll give them those papers to write what ever the question is, just to write whatever they feel about us. And so that’s how I will pick the problems from the patients if there are any. And then to the staff, I like them to do like myself, I know sometimes is like a little bit under pressure but to even if you are under pressure don’t show it to the patient, don’t show it. If you don’t have a time I’m also here, I’m part of you, just call me and say matron come and help here, because sometimes you want to change the routine and here is the patient we are stuck with her, she wants this, she’s impossible so I said no, call me and I will go and talk to the patient. Ja, is to my staff and we do some things in the ward just for team building, mustn’t be work, work, work all the time. So sometimes I’m there doing somethings cheering us up.

Researcher: so I think that what you just mentioned now was you last one is building up to my next question. How do you as a unit manager practice relational care, relational care to your subordinates.

Participant 1: aaa there’s lot of things that you can do to my staff to, to show that I care for them, like I said when they are, you know your staff in the first place, you know so you can see ey this one today is not in a mood so you try to bring her closes to you and said are you still fine are you okay they will said yes I’m good. You know today im okay but this and this. Then you talk to her then you try to solve whatever it might be and if they having real problems, we are having like the EAP here employee programs where I can refer them if sometimes the other mos they feel okay I got a problem but I cannot tell you, then theres somebody you can call and come talk to them. Or sometimes I just call the social worker of the EAP and say I don’t want the whole people, I just want you to come and talk to us, just talk to us, and then she will talk ill be also present. She will talk to us, you know, put us in that position even if it doesn’t show like that but just, sometimes even if you hear somebody saying this and this or pat your shoulders, then it gives you that encouragement. So it goes along with them, that if they’ve done something good, you give them a praise, give them praise, give them motivation, we encourage them to apply for higher post for study leaves that’s how I uplift them, that’s how I build a relationship with them, my staff.

Researcher: That’s nice. How do you as a unit manager practice human connectedness with respect to managing your unit.

Participant 1: The human one is like, like I say you must know your personnel. You can see if theres somebody today, is sad then you go a little bit deeper, you want to help. You cannot let somebody who is not, even if she’s not sick physically but emotionally you can see she’s not fine then you talk to her and then come like come closer deeper into her and said how can I help, how can I help because it might be a problem at home. See hoe you can help. that’s why I said sometimes it’s difficult somebody would or refer, you know now these people they think you are inquisitive, but you can see, you cannot work with somebody who is not happy, who is not happy, ja.

Researcher: Okay we almost done. Two more questions than we done. Okay. So what are the advantages of nurses and managers' practice presence. What can everybody gain from that, what is the advantages, if they’re practicing presence within the ward to the patients.

Participant 1: I think the advantages will be, if they are being treated with dignity you know showing the humanity, then they are willing to come to work every day to help the patients you know do all those things. If you are close with to them as a manager, if you are close to them as a manager, then I think you can go a long way with them, you must not distance yourself from them, from them all the time.

Researcher: And what is the disadvantages if nurses do not practice presence. What can go wrong in the ward, if they are not there practicing their presence, how can this affect actually the patients in the hospital.

Participant 1: That one can kill the emotions of the patients of their colleagues if they are not doing the right thing. So so many things can go wrong, so many things can go wrong if really they are not happy, they are not coming to work because who will nurse the patients you know. The standard will go down, I think so. And ag, mostly the standard will go down if they are not happy if they do not come to the patient and the patient also will not be happy to be nursed by somebody who’s not happy or who will nurse the patients if they don’t come to work. Ja there will always be problems because this didn’t help up correctly because somebody didn’t come to work so it will be a lot of chaos. So the right thing like I said is start with them in a right way. I know the will be the others that will be sluggish that won’t agree with you but try, ja just try and if it comes to that push that this one you cannot manage, you refer her somewhere, but you cannot leave a problem being unattended because at the end it’s gonna be a disaster.

Researcher: If you have to define presence, how will you in your own word define presence I nursing.

Participant 1: In nursing. The presence is like now neh, like how is nursing now. Do you want me is it something like that. I don’t know if I get you right, don’t know if I get you right.

Researcher: Can I try and explain more, okay

Participant 1: Okay

Researcher: When I talk about presence I mean my or the dictionary will explain like being there

Participant q1: Okay the presence. Okay.

Researcher: So what is your understanding when we talk about presence.

Participant 1: in nursing and ey, the presence in nursing it should be there, it should be there because if its not there, then who will guide people, who will encourage people, so it must be there. It must be start with the nursing above there, the nursing manager there. If they are with us as there the operational managers on the floor, we will also be there for the lower people there. So it just come from above until down there because if its not there, then we are breaking a chain somewhere and then it will never, it will never go well.

Researcher: Thank you very, very much. I really appreciate you affording your time to me and I really appreciate it, thank you very much. If there are any follow-up questions, I’ also new in research so if they maybe busy with analysing the data and more data is needed, you said earlier on its fine if I should come back to collect

Participant 1: You are welcome to come back and if you didn’t understand me properly you can come back to me or even if myself if I forgot one point ja but this one I should also mention it. You more than welcome

Researcher: Currently there is nothing else maybe I didn’t ask something that you want me to ask. I s there anything else that you would like to add. I think you touch most of the things and im glad its mostly the relationship between because people will come with how do you do this how do you do this, you must do this but yours it was broad you know to get our feelings how we do things ja and how do I relate with my staff how do I relate to my patients and how do I relate with my management. So I’m glad you asked that.

Researcher: Before I finish off how many years are you in practice as a unit manager.

Participant 1: You cannot believe it’s only short time, two years. It’s 2 years because I like bedside nursing, I like it, so to be here is like because the matron who was here resigned okay and then the post was advertised so I liked the unit as much as I like, you know I don’t like if it can be somebody coming here. Okay she’ll manage but I know exactly how I want the unit to continue running, yes.

Female; Age- 59 yrs; 2 yrs unit manager

**Participant 2:**

[Researcher: OK. Is more than welcome to postpone if you feel like postponing you are OK if I record the session. Yeah, OK, fine. If I don't cover everything and I need some follow up questions, it's fine if I come back at a later stage. OK. Thank you very much. How are you today? OK. OK. Thank you very much. So my research goes about the presence practice amongst unit manager in this hospital. So can you tell me? What, according to your understanding, what is presence practice in this hospital as a unit manager?](https://livenmmuac-my.sharepoint.com/personal/s219266212_mandela_ac_za/Documents/Transcribed%20Files/Part%202%20universitas.m4a)

[Participant 2: UM I think that is something that one has to sort out for yourself personally because we are also people with different personality types and so forth. But for me definitely I think it is part of being here being visibly here, having an open door policy, something like that, so you need to be there for your patients for the parents because sometimes there's parents involved. It's not only your patient and then also you have to be there for your staff which is very important these days. Yes, emotional, physical, all that you have to be holistically present if you can say it like that.](https://livenmmuac-my.sharepoint.com/personal/s219266212_mandela_ac_za/Documents/Transcribed%20Files/Part%202%20universitas.m4a)

[So you mentioned you have to be there for them. Can you give examples of how you can be there for your patients and how you can be there for your subordinates?](https://livenmmuac-my.sharepoint.com/personal/s219266212_mandela_ac_za/Documents/Transcribed%20Files/Part%202%20universitas.m4a)

[Participant 2: For the patients, I think you must just be. They must know who the matron or the in charge of the world is, so that at least when they have questions and so forth, or want some clarity about certain types of care, at least then they can actually ask the right person, or ask to be put in contact with that person specifically. And for that purpose we also have pictures in the corridor where underneath the OPM (Operational Manager) is actually clarified. So I think that also helps a bit, but sometimes the parents are so overwhelmed about the physical area that they don't necessarily see it on their very first day. So I try to hmmm greet them when they come in, but I'm not always there when we have met a patient or get a transfer from one of the other areas. So yes, you have to be there. What I do also is during certain times when they are there, all the parents for instance, then I go around and I greet them and so forth and also ask are they OK, give a little bit of comments about their child and usually then they start asking questions about like picking up weight or how did the feeding go or something like that? So definitely they have a need for that and specifically also when there is some I don't want to say conflict, but in clarity’s that's also good for them to know who to talk to in the end. For the staff these days, I must tell you I've been here in this position for quite a while, so actually 20 years now. But for the staff these days, you have to be more present emotionally. You have to be safe physically as well. Otherwise it is like oh, this matron is just in the office the whole day long. She never sees us or we can't see her or whatever. So you have to have that open door policy for them specifically and then. The emotional support is really, really very important because. All of us have more troubles these days than before. There's more economical troubles, social home, or sometimes it's it's bad for the staff at home, the marriages, the kids. So it's so important that you can be physically, emotionally being able to support them and the unfortunate of it, is that all of us that are in the caring profession. Our cups empty very quickly because there's so many people that drink from the same cup that in the end you yourself find yourself in trouble.](https://livenmmuac-my.sharepoint.com/personal/s219266212_mandela_ac_za/Documents/Transcribed%20Files/Part%202%20universitas.m4a)

[OK.](https://livenmmuac-my.sharepoint.com/personal/s219266212_mandela_ac_za/Documents/Transcribed%20Files/Part%202%20universitas.m4a)

[Participant 2: That's more how I see it and I don't know if there's other views of it as well, but definitely that's that's one of my experiences.](https://livenmmuac-my.sharepoint.com/personal/s219266212_mandela_ac_za/Documents/Transcribed%20Files/Part%202%20universitas.m4a)

[OK. Thank you. And you just mentioned. In our profession, the cup. What was your direct words?... runs empty. Can you explain more about what, what what is the symbol behind the cup? Yeah.](https://livenmmuac-my.sharepoint.com/personal/s219266212_mandela_ac_za/Documents/Transcribed%20Files/Part%202%20universitas.m4a)

[Participant 2: OK, so there's this saying of you cannot drink from an empty cup, and that's actually what I mean. So if. The unit manager, operational manager. Name it what you want to is actually emotionally and psychologically drained she cannot support the rest of the people, neither the patients, neither the staff, neither the family members, nobody. And that is one thing that I have experienced myself that. Psychologically, emotionally. You get really, really very drained. And remember, we have been in the same circumstances with COVID, with all those things. That trauma that we experienced during that then your subordinates than your patients. So I think all of that contributes to the fact that. Your cup runs empty at some stages. I always say that people think we are Jack of all trades, but it's not like that. And in the end, we have a lot of workloads and I mean you do 8 hour duties, you do admin duties, you do whatever duties there are and you must stand in. If you don't have personnel in your ward, then you must cover your words specifically. So definitely. There's many times or many opportunities for your cup to run empty.](https://livenmmuac-my.sharepoint.com/personal/s219266212_mandela_ac_za/Documents/Transcribed%20Files/Part%202%20universitas.m4a)

[So as a unit manager. How can you ensure that your cup doesn't run empty? How can you make sure that your cup gets refilled? And how do you refill it?](https://livenmmuac-my.sharepoint.com/personal/s219266212_mandela_ac_za/Documents/Transcribed%20Files/Part%202%20universitas.m4a)

[Participant 2: That's $1,000,000 question. I really think it also depends on personality types. I do try to exercise at least twice a week, but I don't always get there twice a week. Sometimes you are just so drained and tired that you're not physically able to go to training even if you have booked it a session or what. And then obviously you must do things that you like. That's, that's the one thing of it. Unfortunately, I have a problem with all of this because I'm a perfectionist. I want to do things. In a certain way and in the end I'm very hard on myself and I drive myself very, very hard. In to the extremes, actually. So I'm just back from two months of sick leave due to burnout. Now, so unfortunately I'm not the expert in getting my cup full.](https://livenmmuac-my.sharepoint.com/personal/s219266212_mandela_ac_za/Documents/Transcribed%20Files/Part%202%20universitas.m4a)

[OK. Thank you for that. If you have to define presence, practicing presence, what will your definition be about? What is practicing presence actually?](https://livenmmuac-my.sharepoint.com/personal/s219266212_mandela_ac_za/Documents/Transcribed%20Files/Part%202%20universitas.m4a)

[Participant 2: I don't actually get exactly what you mean. Because I have spoken about the emotional physically and all those presence just if you can refine that.](https://livenmmuac-my.sharepoint.com/personal/s219266212_mandela_ac_za/Documents/Transcribed%20Files/Part%202%20universitas.m4a)

[That So what is your definition about presence? How would you define presence?](https://livenmmuac-my.sharepoint.com/personal/s219266212_mandela_ac_za/Documents/Transcribed%20Files/Part%202%20universitas.m4a)

[Participant 2: Well, presence is being there in the moment and presence is also felt even if the person is not in the room at that very stage, but presence. I always think that operational managers can put a stamp on their areas. So that people will say, OK, but oh, I can see she's back from leave or I can see she's here again or something like that because. Not to say that you are missed, but in the end to say that there are certain ways that you like things being done and your personnel has to do things in the right way and not that doesn't mean only my way is the right way. It means like the standard of care, everything like that.](https://livenmmuac-my.sharepoint.com/personal/s219266212_mandela_ac_za/Documents/Transcribed%20Files/Part%202%20universitas.m4a)

[So yes. OK, thank you. How can user unit manager practice human connectedness? With your subordinance.](https://livenmmuac-my.sharepoint.com/personal/s219266212_mandela_ac_za/Documents/Transcribed%20Files/Part%202%20universitas.m4a)

[Participant 2: Once again, that open door policy, very, very important, because they have to feel comfortable enough to come and see you in the office or in the ward or wherever, depending on what the privateness is of the subject that they want to discuss. And also, support them because a lot of times they just want to ventilate their feelings or their frustration sometimes, so it's not to say that you can physically do something about it always, but where you can at least do something about it and try and improve the the situation that we all are in. Yeah, I would say that's more or less, yeah.](https://livenmmuac-my.sharepoint.com/personal/s219266212_mandela_ac_za/Documents/Transcribed%20Files/Part%202%20universitas.m4a)

[OK. Thank you. What is the advantages would you say? Umm. Of nurses. Practicing presents.](https://livenmmuac-my.sharepoint.com/personal/s219266212_mandela_ac_za/Documents/Transcribed%20Files/Part%202%20universitas.m4a)

[Participant 2: Well, I think communication definitely is better in the end. Because you are open for communication and your subordinates know that you will support them. Umm, maybe Team Spirit will definitely be better motivation behind everything. The staffing motivation will also be better, but even though you say we say that we want to be there in present or being present. It we cannot work miracles if a system is not working correctly on oil wheels or there's a lot of hiccups or whatever. It's not always to say that we can physically change the situation or. Say for instance, you don't have a certain kind of consumable. Doctors, sisters, everybody will get frustrated and highly frustrated. And then you have to be the sound board for that. And one of our duties is definitely to get stock so that the consumables are here, order it as it should be and so forth. But a lot of times that. Ordering has gone through. You've done your part, but the rest of it. There's no stock or something like that in in the warehouses or wherever. So then your frustrations or their staffs frustrations actually becomes your own frustrations as well.](https://livenmmuac-my.sharepoint.com/personal/s219266212_mandela_ac_za/Documents/Transcribed%20Files/Part%202%20universitas.m4a)

[OK. How does? The lack of these consumables impact you on executing your duties in the ward.](https://livenmmuac-my.sharepoint.com/personal/s219266212_mandela_ac_za/Documents/Transcribed%20Files/Part%202%20universitas.m4a)

[Participant 2: Well, definitely a lot because in the end. Everybody is complaining to the OPM asking me, where is this where’s that where’s this where’s that even the doctors will come and say, but we need this to do 123. So definitely it impacts us very negatively. If you go through a phase where there is a certain type of consumable that you use a lot on a daily basis. And that's not available so definitely it will be a negative impact and in the end, I myself would like to provide my patients with everything I need they need to get healthy and to go out of hospital sooner, but our hands are sometimes really tight and we get a lot of frustration due to that fact.](https://livenmmuac-my.sharepoint.com/personal/s219266212_mandela_ac_za/Documents/Transcribed%20Files/Part%202%20universitas.m4a)

[OK. To go back, my eyes falls again on the cup that ran empty as a unit manages. What support do you get to ensure that your cup never runs empty?](https://livenmmuac-my.sharepoint.com/personal/s219266212_mandela_ac_za/Documents/Transcribed%20Files/Part%202%20universitas.m4a)

[Participant 2: Umm I've been here for such a long time and and, and I must say that, sometimes you get more support than other times in the sense of not only just nursing management, but also your doctors that actually works with you or the ones that's in charge of your units. So UM, a lot of times. The support is not always there and the support changes from person to person, so yeah, you get through stages where support is brilliant and other stages with these lack of support. And delegation is high on the list. So it's really a problem and I think we're or that influences actually my as an OPM motivation in doing my job. Because, If you don't have or, you don't have that feel in your head that you can go to anybody when there's a problem or so forth Uhm, it's difficult to do your duties that is expected of you. Also, the other thing that I also just want to say is it's not always just the work situation that you are in. It might be a personal situation at home or wherever as well. That actually influences that cup to become more drained more quickly, specifically if everything is happening all at once. That can be a very unfortunate situation to find yourself in. Uhm, ja support little things sometimes goes a long way. If I can think. We get excited if we get new equipment. We get excited if there's a consumable coming that wasn't there for a while. So immediately it pushes your morale up. If you can say yes, I finally got this, we can use it now again. Other things also like. General infrastructure. If you have to struggle against infrastructure. Problems that you experienced then also it gets difficult because that's a big thing that has to be done and. Say for instance if, if there was an example, now of a tea room that was renovated as part of Mandela Day. I mean, obviously it will lift up the staffs morale because to go to a tea room where the chairs are broken and you don't have a microwave or things like that. It's not really fun. Not that it must be fun, but still it it's a break that you can take and it feels better if you do it in a in an aesthetic environment rather than doing it in an environment where paint is falling from the walls, chairs are broke and things like that.](https://livenmmuac-my.sharepoint.com/personal/s219266212_mandela_ac_za/Documents/Transcribed%20Files/Part%202%20universitas.m4a)

[OK. Thank you. So we've spoken about your the cup, you said it's both work related and personal related. How do you draw the line between work and in personal life, is there a balance? How do you balance it?](https://livenmmuac-my.sharepoint.com/personal/s219266212_mandela_ac_za/Documents/Transcribed%20Files/Part%202%20universitas.m4a)

[Participant 2: Yes, there's definitely a balance. We always are taught that you have to leave your personal problems at home. You must come to work and then you do your job. So obviously there is a balance, but. Sometimes it just goes out of balance because of the fact that there might have been a few things happening all at once at the same time. Work, personal life, whatever situation it is. UM. That can actually now in the end influence you more than it would have if everything was OK at personal level or whichever level you are thinking of. So I would say both those levels are actually in a sense equal. Because if I'm not a whole person, how can I physically perform my duties in a whole way in an excellent manner on the standard, even sometimes. So that's also something that we as operational managers must also think about that can happen with your staff. If you see these things happening with them, all the experiences that impacted them negatively on personal level, obviously you can think that person's performance in a whole would go down a little bit. So definitely there they also need our support, but it would be great if we can be supported as well in the in the same sense.](https://livenmmuac-my.sharepoint.com/personal/s219266212_mandela_ac_za/Documents/Transcribed%20Files/Part%202%20universitas.m4a)

[OK, so how does a leadership role? As a manager, your leadership role, how does that influence? You practicing presence in your in your in your unit.](https://livenmmuac-my.sharepoint.com/personal/s219266212_mandela_ac_za/Documents/Transcribed%20Files/Part%202%20universitas.m4a)

[Participant 2: I would say definitely if you are a leader, you actually have to be able to involve your subordinates in whatever decisions is going to be taken for your unit. Uhm so, definitely just involving them will also increase your practice in presence in a leadership situation. I've now forgotten the word, actually participative management. That's the word I'm looking for. So at least if you practice that type of management. I think people will also feel responsible for the unit and take some ownership for the unit and that would be the way that I would say that that you can demonstrate your presence in a leadership role now.](https://livenmmuac-my.sharepoint.com/personal/s219266212_mandela_ac_za/Documents/Transcribed%20Files/Part%202%20universitas.m4a)

[OK. Thank you. And then? We've spoken about. Practising presence, what is the advantages? What will be the disadvantages if nurses as well as managers do not practice presence in the unit?](https://livenmmuac-my.sharepoint.com/personal/s219266212_mandela_ac_za/Documents/Transcribed%20Files/Part%202%20universitas.m4a)

[Participant 2: Well, I think one of the things would definitely be pasients complaints would go up. It would definitely rise. I can't think that patients will be comfortable. Not having everybody there and being present in the moment and seeing that people are doing the right thing at the right times, and I think there's nothing as bad as seeing people sitting in the tea room all day long, drinking tea or chatting with one another but ignoring the patients. So I think that's definitely one of the disadvantages that will be noticeable. UM. My cup ran empty. Yeah, so that definitely is. But I also think it depends on the personality type of the leader. The more sensitive the person, the quicker it might happen. The less sensitive the person. Empathy is not always there. Maybe so, I think a person who's not having empathy will never burnt out, I don't think so.](https://livenmmuac-my.sharepoint.com/personal/s219266212_mandela_ac_za/Documents/Transcribed%20Files/Part%202%20universitas.m4a)

[OK.](https://livenmmuac-my.sharepoint.com/personal/s219266212_mandela_ac_za/Documents/Transcribed%20Files/Part%202%20universitas.m4a)

[Participant 2: Yeah, I can't think at the moment of any more disadvantages. Yeah, you are too quick on me now.](https://livenmmuac-my.sharepoint.com/personal/s219266212_mandela_ac_za/Documents/Transcribed%20Files/Part%202%20universitas.m4a)

[OK. And on the family?](https://livenmmuac-my.sharepoint.com/personal/s219266212_mandela_ac_za/Documents/Transcribed%20Files/Part%202%20universitas.m4a)

[Participant 2: How could that impact on the family of the patients in the hospital, the family of the patients? Same story. I would think, depending on whether your patient is a minor or not. Or even a geriatric patient, or a patient cannot speak for him or herself. They will also be feeling negative about the very institution where the patient is being nursed. They might feel unhappy with the care that the patient is getting. Obviously they are also open or we are open for them also to complain not also not only for the patient themselves and specifically whether it’s a minor, obviously it will be the father or the mother that launches the complaint or lodges the complaint. So ja I think, knowing from their sight if they know that we care and we, we have empathy with the situation they find themselves in. It will be best for the the family members and the patients themselves.](https://livenmmuac-my.sharepoint.com/personal/s219266212_mandela_ac_za/Documents/Transcribed%20Files/Part%202%20universitas.m4a)

[And then lastly, how do you as a unit manager practice practice that human, human connectedness and relational care with your subordinates?](https://livenmmuac-my.sharepoint.com/personal/s219266212_mandela_ac_za/Documents/Transcribed%20Files/Part%202%20universitas.m4a)

[Participant 2: Well, I just think communication there is the number one that that should be happening. If there's not a two way communication happening, then unfortunately I don't think even being on the floor will help you being present. And that's the only thing really that I can think of that I can physically do and. Yeah, supporting everybody. Where they need support, knowing your staff, seeing when they are having an off day or not feeling well, asking them about them. Things like that, little things sometimes, as I said, may count. It's not only just the the big actions, the shiny actions, that you see in the newspapers that will that will count for for this type of relationship between staff and supervisors.](https://livenmmuac-my.sharepoint.com/personal/s219266212_mandela_ac_za/Documents/Transcribed%20Files/Part%202%20universitas.m4a)

[And when you see one of your staff is having an off day, how do you go about intervening in assisting?](https://livenmmuac-my.sharepoint.com/personal/s219266212_mandela_ac_za/Documents/Transcribed%20Files/Part%202%20universitas.m4a)

[Participant 2: Well, sometimes, for instance, when they are busy and there are a few people I will assist but if I can't assist, I will do just give them a little something like for instance a cappuccino sachet that they can enjoy on tea time or something like that and they do appreciate those little gestures. That's if the group is now under pressure. But if it's a person specifically and I notice that there's one of them that Is off or have a problem then I usually call them aside to the office and then we talk a little bit and things go better afterwards. Uhm, For instance, we had now, recently somebody that was almost hijacked, one of the nurses and on her return from what after it happened and she was now on sick leave coming back. You could see that she's not herself. And I called her to the office and I asked her exactly what happened and she relayed to me what happened. And then I also referred her to EAP, so that at least she can be supported there because in my opinion, she's got a little bit of PTSD and also needs trauma counselling, so you have to be able to do those kind of things. There are sometimes people that don't want to be referred to EAP, they want to go on their own terms and go to their own private doctors and things like that and there’s other people that will tell you no, matron. You know what? I'm OK now, but if I don't feel OK, I will tell you I will. I will ask you then to refer me. That's literally, I think that they must just feel that you are there for them.](https://livenmmuac-my.sharepoint.com/personal/s219266212_mandela_ac_za/Documents/Transcribed%20Files/Part%202%20universitas.m4a)

[Thank you very much. Is there anything that I maybe did not ask that you would like to speak about regarding the topic? That you feel passionate about any, any, anything that you'd like to add? Maybe I've forgotten about something that I should have asked. Do you think I should ask something different to help me? Definitely.](https://livenmmuac-my.sharepoint.com/personal/s219266212_mandela_ac_za/Documents/Transcribed%20Files/Part%202%20universitas.m4a)

[Participant 2: It's just that you know what we do these things, but we don't always think about it. So for us or for me personally now to quickly think about what I need to or what I think is the is the way I'm practising things. Is a bit difficult, but it's good to have this knowledge and exposure, I would say for myself, to actually think, but actually there's a name for it. What you've been doing is is there, but there's physically a name for that and you don't always think that there is a name for that type of presence you want to say it like that. Yeah. Yes.](https://livenmmuac-my.sharepoint.com/personal/s219266212_mandela_ac_za/Documents/Transcribed%20Files/Part%202%20universitas.m4a)

[Thank you very much. If I have follow up questions, I will definitely if you if anything more that you'd like to say that if you can think about something. Feel free to. Call me. OK. OK. Thank you very much. OK.](https://livenmmuac-my.sharepoint.com/personal/s219266212_mandela_ac_za/Documents/Transcribed%20Files/Part%202%20universitas.m4a)

Female; Age-51; 20 yrs of unit manager

[**Participant 3:**](https://livenmmuac-my.sharepoint.com/personal/s219266212_mandela_ac_za/Documents/Transcribed%20Files/Part%202%20universitas.m4a)

[Researcher: Good day, ma'am. My name is Bernadine Smith. I'm a master’s student at the Northwest University, and my research is about present practice amongst unit managers in a selected hospital. Thank you very much for affording me the opportunity to do my research with you. If you have any questions, you can ask me, OK. There's no right or wrong answer. All that is needed is your point of view, OK? If possible if I do not cover all the questions or follow up questions, is it fine if I can contact you afterwards as well, thank you very much and you don't mind me recording it?](https://livenmmuac-my.sharepoint.com/personal/s219266212_mandela_ac_za/Documents/Transcribed%20Files/Part%202%20universitas.m4a)

[Participant 3: 100% with me. No. I don't mind.](https://livenmmuac-my.sharepoint.com/personal/s219266212_mandela_ac_za/Documents/Transcribed%20Files/Part%202%20universitas.m4a)

[Researcher: OK. Thank you very much. So, my first question will be; What is present practice amongst the unit managers?](https://livenmmuac-my.sharepoint.com/personal/s219266212_mandela_ac_za/Documents/Transcribed%20Files/Part%202%20universitas.m4a)

[Participant 3: In my ward I like to be always available if I think of my night shift, my day shift. I come normally anytime from 6:10 in the morning that I can meet with my night staff that if they do have any problems or questions or things or i have information to to give to them. Then I come normally I every morning anyway, I come 10 past six 6:15 to work. And then all information that I received from any area in hospital, like from my managers, I will also give to every shift that information. Then also on my patient care or and before patient care, nor on nursing staff my I've got an open door policy so anytime they have we have WhatsApp and we communicate also like that. Otherwise they come anytime to my office. And then on patient care, every morning I go and greet all the patients and all my staff also. If I'm busy in the office and we admit new patients, they immediately come and show them where the manager's office is. That if there's any complaints or problems that they can come to talk to me. So that is basic what we do so mothers know I'm always available. So we just circulate all the information all the time to them, yes.](https://livenmmuac-my.sharepoint.com/personal/s219266212_mandela_ac_za/Documents/Transcribed%20Files/Part%202%20universitas.m4a)

[Researcher: OK, so if you have to define how would you define presence in a ward?](https://livenmmuac-my.sharepoint.com/personal/s219266212_mandela_ac_za/Documents/Transcribed%20Files/Part%202%20universitas.m4a)

[Participant 3: Presents mean I must be all the time available. So, for me I must be on board if something happen or good or bad. With doctors being there for the staff if I see something that they are not happy with maybe with the doctor or something to also follow that up. Same story with patients. If a patient is complaining, maybe a doctor didn't visit them for the day and then I must follow up and then go back to them. To be present I always feel is that you, you hear stories out and you report back to them then it means the circle is being completed. So, for me being always around neh they must know exactly what is what I ask from them, what's needed from them and then if not, I need to go back to them and try to correct things like that, so being present is to be always available for them, but with answers and also to listen what questions they have.](https://livenmmuac-my.sharepoint.com/personal/s219266212_mandela_ac_za/Documents/Transcribed%20Files/Part%202%20universitas.m4a)

[Researcher: OK. You mentioned something about following up of complaints. What will the complaints mostly be all about?](https://livenmmuac-my.sharepoint.com/personal/s219266212_mandela_ac_za/Documents/Transcribed%20Files/Part%202%20universitas.m4a)

[Participant 3: On nursing site complaints normally come in where a doctor don't hear what they ask or what they need. Then I will follow up. Or if it feels for them that the doctors maybe don't hear them out now you you tell them something you querying medication and then the doctor will say no, it's fine like that, not during that they are working along in the department. You know, doctors are moving around the whole time that so complaints like that and then things like patients stay for long times in the wards and doctors don't follow up always on yellow charts or on prescriptions. So then they will come to me that I follow up with their doctors, so we solve the problems normally very quickly and there's no further complaints on on issues like that, that small things normally. On patient side I don't normally don't have very much complaints on what you call it on attitude and things like that attitude is normally a very low degree or things normally come the complaints from mothers is more a question saying that they don't have soap and things like that. And the doctor keeped them longer than they have planned. So we issued them then with some clothes and some toiletries that that can complain can be covered. Other complaints will be things like. The other thing is that I get complaints of mothers once in a while. You will get a complain of a mother complaining about the attitude thing, but they normally we call in the nurse as well and we sorted out that complaint. And normally the mother is happy afterwards. So if they come and complain that I need to go through to quality insurance, then also they will be follow-ups on on that like a written warning and things like that. But that don't happen often at once or twice from when I when I come to this ward it normally it's a thing that we can solve immediately. Or sometimes a mother will complain that they didn't get a blanket to sleep with, but we don't supply mothers we supply the patients neh, so it's small things that we can correct immediately, yes.](https://livenmmuac-my.sharepoint.com/personal/s219266212_mandela_ac_za/Documents/Transcribed%20Files/Part%202%20universitas.m4a)

[Researcher: Thank you very much. And how do you as a unit manager practice, human connectedness and relational care with your subordinates?](https://livenmmuac-my.sharepoint.com/personal/s219266212_mandela_ac_za/Documents/Transcribed%20Files/Part%202%20universitas.m4a)

[Participant 3: OK. Like I said earlier, my I've got an open door policy, so all of them know they can come any time to me with even their personal problems and the confidentiality of around private things. I really take it to heart so and always if they have problems at home. Or see problems not having food or things like that I will refer them, for EAP that we can try to help them say your child is using drugs, how can we, I support them. And then also I told them not to tell the staff the rest of the stuff to why I’m sending them to occupation or something you just, I told them normally just tell you going quickly for doctor's appointment because it is a doctor's appointment. So not knowing what this round that so I think on that stage on that area, I really support them in even home problems and here at work as well. For me there is although we have different ranks, we are one unit we must function together. So, if, say, maybe I've got to complain about you from other staff member I will first hear your story. And I've never used names because I don't want ructions in the department, and if you know, I'm complaining about you, then you don't want to work with me and then we sit with a terrible situation in the ward. So, I normally use my things like in general, when there come a complaint. So if a there is a complaint that one staff member is not helping the others. I first will tell there was complaints that you are not helping or I will do it in a general saying maybe there's more than one complaint of the same type of matter, but not only for one person. I will then generalize it and say there is complaints please people let we help each other fixing this because we are very busy we are short staff let we try to help each other. Yeah, so that's my. I don't know if you need more than that, ok is it fine.](https://livenmmuac-my.sharepoint.com/personal/s219266212_mandela_ac_za/Documents/Transcribed%20Files/Part%202%20universitas.m4a)

[Researcher: Okay, unless there is something else that you would like to mention to that?](https://livenmmuac-my.sharepoint.com/personal/s219266212_mandela_ac_za/Documents/Transcribed%20Files/Part%202%20universitas.m4a)

[Participant 3: Like I said, for me confidentiality is a very important thing. That's why all my files and things are always locked. If I leave the office and that people don't just come in and and for me, if you don't tell someone something, I don't even tell if someone get pregnant neh and they come and tell me they must not wait till they either show or tell the people themselves. So I know a few of my staff is engaged and others don't even know they are engaged because they don't want me to say it so if they have a personal thing that even if it's something good, if they don't want me to mention it, I keep it quiet, yes.](https://livenmmuac-my.sharepoint.com/personal/s219266212_mandela_ac_za/Documents/Transcribed%20Files/Part%202%20universitas.m4a)

[Researcher: Okay. So how does your leadership role impact on you as a manager practicing presence?](https://livenmmuac-my.sharepoint.com/personal/s219266212_mandela_ac_za/Documents/Transcribed%20Files/Part%202%20universitas.m4a)

[Participant 3: UM. Like like today neh if we had met I I try always to hear the story out neh I normally I'm early morning here to listen to the report so if there is problems that I can solve it immediately that the people that's on duty can cope with their job and like I said earlier, I tried to follow up the whole time. So, if someone come and say we are short of staff I will somme, I've got groups on my phone, even with other departments on it, to try to solve that problem for them. So other manager things I I really help where I can to let them train neh, give them the opportunities. It's not everyone that is accepting the opportunities you you know that I've tell them several times of this knowledge hub as well to better yourself. So, wherever I go if if I get any information from like from the doctors, that is interesting information and things I also share it with them as well because I always I feel if you are willing to learn, I will give you all the opportunity. So, some of my staff even lower categories will come to me and say, can you learn me how to check the defib then I will do it or I will ask one of the sisters because gain, then for her it it means that it will also better her by knowing already that I do have this and and my team is back up backing me up. So in in anything that we do, I'm always their back up. So, for me it's very important that they get better skilled. And even if someone want to move out of the department, I will back them up, I said I can't back them up more than only from my side because what happened at times is that, say, maybe you want to go to Welkom because it's nearer to your home. You already told me you have family problems and it's difficult from working from this site and things. Although and when I talk to them, I will explain to them, although I signed consent that I will let you go, do know it won't be approved. I'm telling them sommer straight and it won't be approved because already we are short. So next level about me won't approve that they know what is going to happen. But I do approve because for me, the fact that I was short of the shortness of staff then it's the bad part, but in the end of the day, I can't keep someone here if it's not making them happy neh. So, from my side, I will always agree, but I don't know what management after me will do so yeah for me I Yeah. That's what I can say to to that. I don't know if you need more or different type of information.](https://livenmmuac-my.sharepoint.com/personal/s219266212_mandela_ac_za/Documents/Transcribed%20Files/Part%202%20universitas.m4a)

[Researcher: That is OK, so you said Senior management. So how does senior management their roles impact on your, on your duty as a unit manager to practice presence?](https://livenmmuac-my.sharepoint.com/personal/s219266212_mandela_ac_za/Documents/Transcribed%20Files/Part%202%20universitas.m4a)

[Participant 3: Again, here, you see, just now the call was my assistant manager, so I just ignore her now but normally now so normally if if they have anything that is new or things that we must do, or information that we must convey to the departments they will give me the information, sometimes good and sometimes bad information, and then we just take it further. I always say we mustn't play each other neh. You must use the information to, to and and give the information as positive as possible. Like now again you know all this what is it CPD points that everyone must get. So people are very negative about it because if you don't get it, so the way you give them the information I said, we are giving training every day, single day of our lives. Just make sure that you capture it somewhere it goes through as CPD points, so it's part of your job neh doing and giving education. So, use it in a positive light. Just let someone witness it and make sure that's that that person signed for it. I will make sure that there's enough papers there or even in the communication book let them just sign that you have given the information. So, most of I've got my small form yearbook here if I went to a nurses meeting so once a month, all the operational managers come together to give information or to get information from the head of nursing. So, all that information, the that next morning because it's Wednesday, so normally the shifts are changing on a Friday so on Thursday morning I will give all that information then to that shift. Then in the morning I will give to that night shift and then the next day I will give to the new shift and then when the new night shift come on duty, I will also convey that information again. But normally most of our the biggest information that causes trouble there is maar shortage of staff and if we don't get new staff from management, but what I always tell them is what I ask but we don't know what we're going to receive, so it's not a one way thing here because if management asks, maybe for a 100 new members, head of Department of Health can maybe only give us 20, then maybe we will get one if we are lucky. So that is mostly the the problems that when we give the information over or things like they want to work more overtime and they're only allowed to work 24 hours. You know, things like that. But we we continue with what we have or the uniforms and then always when there's money involved then you feel the things. But even here, if there is coarses again, it's maybe more on previous questions that you have asked. When staff need money for training, I go to my doctor's because we are a team and ask for donations and things to pay for the courses for them, you know? So I really try from my side to to get my my people educated and get the the knowledge because for me I always say if I have all I'm talking a lot. So all my information I don't like to keep it for myself. For me to give them, and I'm very strict on or very strict, strict, very straight, whatever come to me I want to give it over to you like that. So, the quicker I can start doing it the better for myself. So, but motivation I am very positive and I'm giving my staff to try to motivate them so even if it's something bad, I always say let me see the the bright side on it, because in the end of the day, the more positive we stay the better nursing care you can give, but I'm always around here. If I'm the they're not here the people will say what happened to me. Even Mme will tell you if she is not there she must be really sick. So, I'm always around and helping all over the hospital, and that's also something that I say to them, remember it don't help we keep our knowledge for ourselves, so even if we need to go to other departments and share our knowledge, use it like that. So yeah, I think I've said what I want to say on that topic yes. Researcher: Ok, thank you. What do you think are the advantages of nurses as well as unit managers practicing presence?](https://livenmmuac-my.sharepoint.com/personal/s219266212_mandela_ac_za/Documents/Transcribed%20Files/Part%202%20universitas.m4a)

[Participant 3: I think also there I've said earlier, if number one if my if the unit manager is available and present it makes your staff to be also more present because most people follow the leader, so if you are there for others neh you see I even want to cry now because my heart is in nursing neh. So, if you do what is expected of you, your nursing staff will follow and the the moment you’re your registered nurses following you can also see your lower categories start following neh, and in the end of the day that is what we need to to make a fire to burn proper neh, I always say if there's one of us not being part of this challenge, we are a broken chain like cause problems in the department? So if we can hold hands and I said if everyone just have a small fire, we can make a big fire and then everything can go well in in the department. So presence on all the different categories what I also say even to my my rest of my staff, you know your lower categories, like your, your cleaners and things. If your cleaners are not available you don't have a clean ward. If you don't have a clean ward. You’ll have nurses to leave their job and attend to cleaning. Then you have infection that is higher in the department. So from your lowest category in the department, you need them all to be part of this chain and I continue saying that to to them the whole time as needed as much as they need the manager in the department they need all those categories that is in the department. They need all of us to to be present and that we stand in for each other. Like at the moment I've got a crisis. I only have two cleaners neh for day and night shift, so I'd rather put them on day because in the during the day it's more busy and you need more cleaning. So, but they stand in for each other. So, the one was on night shift but when one of the day shift fall sick, that one immediately said I'm willing to come neh. So, and I. I think it's because we always communicate. Umm, communication is one of the most important parts to be present. There you can’t be present not communicating, not only physically but verbally as well, you need to be present all the time, yes.](https://livenmmuac-my.sharepoint.com/personal/s219266212_mandela_ac_za/Documents/Transcribed%20Files/Part%202%20universitas.m4a)

[Researcher: OK. Thank you. Lastly, what is the disadvantages if nurses and the unit managers are not practicing presence?](https://livenmmuac-my.sharepoint.com/personal/s219266212_mandela_ac_za/Documents/Transcribed%20Files/Part%202%20universitas.m4a)

[Participant 3: Number one, nursing care won't be continuing. If there's no nursing staff neh and they are not, or they, even if they are there but not present, you know there's a difference being there not present. You will start getting more complaints. And that is nogal one big issue. So, if the ward is very full and the sisters is not visible the whole time and hear parents out then but for the complaints it will go, it will escalate neh. The complaints will get bigger. There will be more death, the death rate will be higher. So, mortality will increase as it is at the moment we don't have a high care so, my ward is also a high care. So, if we are not present, we will really have a bad mortality rate here and it will really increase. So for for learning for presence with nursing care, for allocation for all that things, if we are not present, everything will just collapse. And again, if, if you just think of operation argg professional nurse, if they are not present, so they will see that nurses maybe don't feed a patient, then as the complaint come in then you don't have backup neh. Because if it's not written it's not done. So, to be present mean you must evidence and write down, so if you don't do it it’s where the complaints and the problems will start. Same story to the manager. If I'm not present, I won't know what's happening in my department and then all those wrong things will happen. Around me and me not knowing about it, and again to be present again. Like I said earlier, I need to inform my staff the whole time. I need to know if there is no stock because if I'm not present, if the sr, number one, I do all my orderings and and everything. So the moment i maybe see no, there's no stock. It will affect my staff as well because I were not present neh so, but all the other things will also be part of mine as well. But basic things people don't think of to be present make sure that all equipment is work is in a working condition. That you have all your stock being part of you being present because if you're not there and if your patient staff don't report to you that they maybe don't have nappies, I will not know if I were not present that I must order, say, maybe I was week-end off I don't know which stock is now finished then they need to come back to me and also report again. So, if you don't do that, the whole circle will collapse again neh. Because one thing feed the other thing. So in every category of nursing being manager neh you need to be in all aspects neh from baseline training to the reason why we are here, the nursing part, all that things in between. If one of the chains or the “skakkels” are not part of of the whole circle, then the next thing will will fall apart, so yeah.](https://livenmmuac-my.sharepoint.com/personal/s219266212_mandela_ac_za/Documents/Transcribed%20Files/Part%202%20universitas.m4a)

[Researcher: OK. You mentioned. There's a difference of being there, and there's a difference of being present. Can you explain for me?](https://livenmmuac-my.sharepoint.com/personal/s219266212_mandela_ac_za/Documents/Transcribed%20Files/Part%202%20universitas.m4a)

[Participant 3: I can come every day on duty and sign that I'm here, but I'm not available neh and that's again where the open door policy came in any time they phone me sometimes at night that makes me present neh, although I'm not now what you call it physically, yeah, but I'm still present, so I know of everything that's going on. And being on duty and the whole time hiding in my office, I'm not present neh. That's why I said it's important for me to not only sign on duty I must be there for them. And that's why for me, it's very important that every shift change that I am follow up if there's problems and things like that because that makes me present. While being actively present neh, just signing on duty you also present neh, but I must be actively present so every now and then as well like now I will go on the floor and just make sure they are all still OK because in the end of the day to to be here and not there for them makes me present but not being part of there, that part of you not really present yes.](https://livenmmuac-my.sharepoint.com/personal/s219266212_mandela_ac_za/Documents/Transcribed%20Files/Part%202%20universitas.m4a)

[Researcher: Thank you very much. I really appreciate your cooperation. Is there anything that you think that I didn't cover that I didn't ask? When it comes to practicing presence as a unit manager in the unit that you would like to add.](https://livenmmuac-my.sharepoint.com/personal/s219266212_mandela_ac_za/Documents/Transcribed%20Files/Part%202%20universitas.m4a)

[Participant 3: Just extra say to you. Like I earlier said to you, for me it's very important that to keep my to being part of present is to do things that make people to be 1 to be coming to this department. If you can look at I I always say if your environment is nice, people want to come there, so that is just something extra. So by doing the thing of the environment, start coloring the walls in different colours. Try let people come and draw paintings on the walls and things make you make also your presence visible neh. Because you work much nicer in an area where everything is positive neh, you can be negative but something that I can just add. I came here in 2017. The department was very negative. The ward was looking totally different and my first thing was changing curtains and make duvets and things not to being put on a pedestal or something like that. But if the environment is positive, I can't fix the things at home for you? But if I can make the environment where you work, where a patient maybe see his last minutes here, maybe before they die and I can make it positive, then I I've done my job for the day neh. So, for me it's very important that patients are happy and and that my staff is happy because if you if you can get that part, you will work harder. Everyone will give a extra more of themselve. If your environment is positive and what you call it? Conducive. Yes, because then you will be giving a bit of yourself and really, if I must really say the six sisters, I only have six sisters. And they really some of the best in this hospital, I think they they really give more than that more they have than, You know they will leave their home behind and come on duty even being sick and we'll just put the mask that they don't harm the patients because they know there's no one else that can fill that position. And if you're not happy when you come on duty, you won't do things like that, yeah. Yeah. So yeah, I think that is what I can say. So for for me being a try to keep give, give them a positive ward to work in, I think you touched them, you touch people's heart, and if you touched a person’s heart they are more willing to give.](https://livenmmuac-my.sharepoint.com/personal/s219266212_mandela_ac_za/Documents/Transcribed%20Files/Part%202%20universitas.m4a)

[Researcher: Yeah, that's true. Thank you very much. I really appreciate it. I can see you do a very great job. From listening to you keep up the good work. Thank you. There's a few left. Participant 3: Yeah, that is the other part. I worked 18 years in private. Yeah. So, when I came to this site to government hospitals, again, I say don't matter where you go because a lot of people will ask you why do you come to government and one of the nurses that always were moonlighting in private in my department were saying I must come and make a change and then I just fill in a form, application form and I got the job and I believe I'm here for a reason. And what I always say to my staff as well that we don't see the negative things that we work on, the positive things, the more we are positive in the end we will get pay back. But we mustn't do that to get pay back neh your recognition in the end of the time, yes I said so for me don't matter where I work neh. If you do it from your heart. Yeah, yeah. You can make a difference. Yes. And here, it's more chance in, in, in public hospital to make a difference. You know, if you're working private, you get a proper salary here salary is much better anyway than in private. But it's us not going to private. So we are very much more demanding and things like that. So here we can mean something to the the the community because a lot of patients that this here don't have any support, so by giving them the support here, we very easily and very quickly get social workers and if you hear social workers, everyone think that someone gonna take my baby from me, neh. And that's not the reason we get social workers. We get social workers to assist the mother’s and I think that is one good service that we can render here the moment the sister feel a mum and that's where we're connecting, if the maybe the doctor not even have realised this mother need a social worker, then the night staff will come and say no, this mum sit at night crying neh and maybe there's something wrong. You didn't want to speak to me, so I will first try and then if I say no, this is really there is a crisis here she don't want to talk about it. We immediately try to get the social workers. The fact that we immediately then follow up with social the social workers, so we go to the doctors and say please arrange for us social the social workers to come and follow up here for us. So we really I think we support the mothers as well, and we also even have a thing like all our mothers been admitted as lodgers, although don't don't, they don't have beds and things. You know, there's no space for things like that. We have a few stretches where they can sleep, so we make turns. And then we admit them, because a lot of mothers are on chronic medication. So then they come near end of the month here, then the medication is finished, then the doctor can just take the medication that they are using. We re-prescribe for them. They normally just give for five days just to, so if they stay long, every time we for five days, we give them new medication. So, I think it's it's good things to to support their mothers with. And then sometimes we bend the rules a bit you know, so it's just small extras if your child is very sick all your families coming from far, and they just go through Bloemfontein. We allow them without, with, not within the visiting times just a quick quick to come and say hello and yeah. That we tried to support because most of the mothers here don't have any support systems, so if we are not there, they don't have any support system. So we really try to where we can to support. Like I said, I've got all the other goodies to assist because a lot of mothers are coming in, they go to the clinic and then at the clinics they tell them you must be admitted. Then they are just straight coming here with the clothes that they have on their bodies. So, by doing things like that, I think also we support them and the nurses also know they can come here. And ask if there's open and things to assist their mothers again. So I I think it's it's something good that we do here just for for something extra maybe we can't because most of the patients in my department is chronic children. Most of them not very good prognosis, so it's a sad ward and to keep everyone smiling, it's nogal a hard job so you need to be present. You need to hear people out. Don't matter if it's lower categories. Even me I need sometimes to just go and rebrief with my manager or with one of the doctors, you know, because we need because of the death rate. Ohh well and and what it is like the the way it's it's like now our of death rate is quite low me for me because sometimes this with the type of illnesses we've got here it's mostly if this months passing that there's no child that's dying. Although it's very bad condition. And I think it's the team is good and and and with my consultants that's working here also because again on my side I need to go to the doctors and get answers and things that we can give the information again back to the doctors and things. So if a nurse, that was one of your earlier questions. Sorry about the complaints that come in. Other things is the prescription shots that's not fully completed and then we don't get the medication from the pharmacy. Again, it's the whole circle. If one don't do the job proper, the next one struggle. So need to go back to the doctor's and after the second time I spoke to the same doctors, then I go to the consultants, you know. That they can talk to them that we all the time, keep to try to keep the the the this truck driving the whole time, neh we we can't stop in between because one didn't do what is expected. Yes. And then we are very quickly also on things like I need to know if a child is for ICU neh, if a child's condition is very poor, I need them to put it on paper for me that they did discuss it. So I want my mothers to know there is nothing that we can do for the child any more. But we won't stop caring for your child, neh. So I will sit on the doctor's neck because again, it makes it easier when we know what to do in this situation like that for the patient or the parent of the patient and for the nursing staff because we will resus every child even if the prognosis is 0. If the doctors didn't talk to the mother and didn't put it on paper for me, yeah, so. I'm I'm trying to do the correct things. I know everyone don't like the fact that we do the correct things. You know, people like shortcuts. I don't like shortcuts. You do this the right thing the right way, then you don't spend so much time. By doing the correct thing so in private there was a lot of things about this. That time must use your time proper, so using your time proper is doing the right thing on the correct way, then you save time. Yes, yeah. And I've said most of the things that I want to have said.](https://livenmmuac-my.sharepoint.com/personal/s219266212_mandela_ac_za/Documents/Transcribed%20Files/Part%202%20universitas.m4a)

[Researcher: OK, thank you very much. I hope if I have follow up questions that I can approach you.](https://livenmmuac-my.sharepoint.com/personal/s219266212_mandela_ac_za/Documents/Transcribed%20Files/Part%202%20universitas.m4a)

[Participant 3: You can come to me again 100%. Well, thanks a lot. Thank you.](https://livenmmuac-my.sharepoint.com/personal/s219266212_mandela_ac_za/Documents/Transcribed%20Files/Part%202%20universitas.m4a)

[Researcher: How many years did you say your years of how many years are you in your position?](https://livenmmuac-my.sharepoint.com/personal/s219266212_mandela_ac_za/Documents/Transcribed%20Files/Part%202%20universitas.m4a)

[Participant 3: In in my position, I'm here from 2017, I were acting for a year and then in 2018 I were I I got the job. So yeah, yeah. Six years? Yeah, 2018, 2019, 20, 21, 22, 23- six years.](https://livenmmuac-my.sharepoint.com/personal/s219266212_mandela_ac_za/Documents/Transcribed%20Files/Part%202%20universitas.m4a)

[Researcher: OK. Thank you very much.](https://livenmmuac-my.sharepoint.com/personal/s219266212_mandela_ac_za/Documents/Transcribed%20Files/Part%202%20universitas.m4a)

[Participant 3: OK, something else that is also important for me is that as manager, you mustn't be strictly on time neh. You must be strictly on time on duties yes but not going home near for me and putting my came on come on duty past six in the morning. And I only put it on on paper 6:30 neh because then it's my normal time coming on duty, but anytime before that I don't capture anytime I'm leaving. I don't capture for me 4:00 o clock is not my knock off time because if the ward need my assistance or they're struggling because there's a crisis in the department. I go off when they don't need me anymore. So sometimes even 8:00 o clock I'm leaving the department. My husband know he’s married to a nurse. And this nurse don't have time. I just will send him maybe a WhatsApp if it's after five. I'm, I'm still coming but it will be later. So for me, there's no time for going home now and coming on duty, I sometimes come 3:00 in the morning on duty because they need training. Then I come that time in the morning. So yeah, they don't like me when I come that time. But if they need training, I need to come and give it and I try not to take their sleeping time away from them because they they work night shift, they need to go home on time. And normally between 3:00 and 4:00 it's a quiet it's more quiet time in the department. So yes, I I come for for all their things during that time of the morning. So yeah.](https://livenmmuac-my.sharepoint.com/personal/s219266212_mandela_ac_za/Documents/Transcribed%20Files/Part%202%20universitas.m4a)

[Researcher: Does your working hours, your working schedule, doesn't it have an impact on your life and how do you balance your work life?](https://livenmmuac-my.sharepoint.com/personal/s219266212_mandela_ac_za/Documents/Transcribed%20Files/Part%202%20universitas.m4a)

[Participant 3: I'm. I'm like I said, my family know I'm a nurse, so my when I'm off my private time is normally weekends. But if the walk work need me, I'll go back there. Luckily for now I'm long married, so my husband understand, neh. So he will just continue. So if it’s me that supposed to cook, he will start cooking. So we fill each others on because in the end of the day, I'm working here with other people's lives, neh. And if you are a pediatric nurse, you work with someone else's child neh and I know how I feel about my children. Luckily, my children are adults now, but I know how I feel about my children and if I can support my staff neh to to give their part supporting other peoples children. I'll do it by all means. When I'm on leave I'm also always 24/7 available my staff knows so my husband also know and he will even drive me and sit and wait for me in the car. Neh just to or come and sit in my office till I'm done and then we leave together. Yes. Now. So we I don't think there's a real balance between work and in my private life because being a manager, I feel you must be 24/7 available if they need you, although they they know also not to to bother me I don't answer the phone for everything neh. They normally must write me a WhatsApp neh, that I know about what it is. Because the sisters, that's on duty have all the rights that I've got. They must make a plan at that moment. If it's something that they can't handle, then they they will phone me. Yeah. So if there's two nurses that is having trouble with each other, it's not my problem now it's the sister that's on duties problem to solve. And there's always a manager in the hospital after hours. If there's first things that they can handle through them neh if it's then above them, they know they can contact me anytime.](https://livenmmuac-my.sharepoint.com/personal/s219266212_mandela_ac_za/Documents/Transcribed%20Files/Part%202%20universitas.m4a)

[Researcher: OK, thank you very much.](https://livenmmuac-my.sharepoint.com/personal/s219266212_mandela_ac_za/Documents/Transcribed%20Files/Part%202%20universitas.m4a)

Female; Age-51; 6 yrs unit manager

**Participant 4:**

Researcher: Good day, ma'am. My name is Bernardine Smith than the L&D practitioner for Free State northern Cape and I'm a master’s student at the Northwest University. Thank you very much for affording me the opportunity to do this research with you. Just a few things. There's no right or wrong answer or that we're interested in is your perspective of the topic. The information will be shared with you if you want us to share it with you and if you have any questions, feel free to ask me. Is it OK if I record this session?

Participant 4: answer – “Yes.”

Researcher: OK, thank you very much. Should I have any follow up questions, is it fine that afterwards that I can come back, maybe just for a few questions if possible. OK. Thank you very much. OK. So, I'll be taking up more or less 30 minutes minimum of your time. Thank you very much. So, my first question. Is what are the present practices currently among unit managers? Broadly, what will you in your own words, what do you think? If you are, I have to give you a few seconds to think about it. If you think about currently what is happening here in your hospital, what do you think is the current presence practices of unit managers.

Participant 4: Yeah, that's a tough one, that's a tough one. I don't know with with the practices of unit managers, but it's difficult. It's a difficult question. What do I say.

Let's come back to that one. If you have to define what presence mean to you present practices. What will you say it is in your own words?

Participant 4: Present practices as what we are doing currently.

Researcher: What is presence to you in a ward with patients. What does that mean? Being present with a patient.

Participant 4: As being there for your patients, and also it will be also about guiding and supervising your staff also to be there for your patients now, now that I'm thinking about it as we we are now talking about clinical governance, yes. Now, with clinical governance, we are accountable for, for whatever that we are doing to our patients, we are accountable. Therefore, we need to give quality care. So, if you are there as a unit manager, you are guiding your staff, you're also involved in the patients care. Then you are able to account and you are able to ensure that they are getting quality care. So, what we are doing currently as unit managers now is we should be having questionnaires we are giving out questionnaires because now when we talk about clinical governments, we are talking about also involving the patients in the care like in neonatal we talk about family centered care with family centered care you not only nursing the baby, you also nursing the parents. So, with parents, the only way you can know if what you are doing as as you approach using quality the only way is to to get a feedback from them. So, we will be having complaints, compliments and suggestions. We encourage them to to feel free to if they have a complain they need to raise their complain. If they have a suggestion. If they have compliments also because it shouldn’t only be about negative positive also should also like if there's anything positive that we are doing to that is what we are doing, and then, yeah.

Researcher: So, you mentioned about the necessary guidance, we talk about guidance, what type of guidance is there that you can actually give your your, your, your, your, your sub ordinance?

Participant 4: OK, like now when you went inside, I was with one of the contract workers teaching her, that's one way of doing it. The support actually it starts with when when we start in the unit it starts with orientation. You need to to to make sure that everyone is orientated and then there must be reorientation for those that are already here, sometimes you find them already working there. There must be that reorientation because you don't know what they know, what they don't know, and then the other thing is that you you will be having with the support also it's it's it's all about teaching actually you will be having in service trainings they are also supposed to give training to others like peer group peer something, yeah, sorry. And then the other one will be, you remember we have direct and indirect supervision, so there are those that you will really because of, I'm sure you are aware that we have problem with shortage of staff in this era that we are in. So at times the other professional nurses won't be there won't be able to supervise them, therefore as a manager you also need to jump in if there are those instances you can do direct or indirect supervision. You do delegation of them or when you do your delegation you should know that this one is really competent you you know, with principles of delegation. Yeah, basically that is the support that you give them and so this thing of taking care of them that is also part of support. You need to be able to see when they are they have challenges and if you have to refer them then you will refer them to EAP you refer them to occupational and maybe the actual claims that they are having.

Researcher: OK. When you talk about challenges, what type of challenges, if you can just name name a few, will they be having?

Participant 4: At times they have family problems. At times it will be the the the very work environment, and you can pick this up from from usually they they they won't be. They won't be attending. They won't be coming to work and then so their their absenteeism will show you you also monitor their absenteeism rate and then you can be able to pick up there is a challenge here you can sit with them at times they become open, they will open up. But then if they don't open up and you're not able to help them and you will refer them. Others will be abusing substances. There are those that are abusing substances. There are those that are that are going through divorce. Maybe the other one has lost a spouse, so basically those are the things.

Researcher: OK. When you first spoke about presence, you also mentioned something about accountability, how you keep them accountable for, for, for, for their actions. OK, so how do you keep them accountable?

Participant 4: OK, when I started with because I joined them I came here in December, they were already here, so I presented clinical governance to them, I think it was a new thing to them. So with clinical governance you explain everything that you are going to account, you need to to, to produce quality, you need to produce results and if you don't do that, then you are going to account. And then as you explain to them, you also organize like there are things that you will not be any expect of. So with me, I also organize training from Labour, that is one way of knowing that if you do this as a misconduct and you have to account for that. So you organise training for them from labour whereby they will be taught about disciplinary actions. They will be taught conducting themselves in the workplace and then also the other thing, because I've already spoke about the absenteeism rate, you also engage HR because at times you will find that they don't even understand these leaves that they are taking and they need to account if they don't come to work, so you also engage HR so that they can be able to explain all the types and kinds of leaves that they are entitled to take and the procedure there of of applying for those leaves. And then now we go to the actual actual accountability let saying maybe there is a misconduct. Then you go to all the letters you will be issuing all the letters, actually it will start with with the first step would be depending on what what they have done it's verbal warning and then written warning. So you will be issuing out all the letters whereby you also advise them that when you answer this, whatever allegations that we have against you, you have to consult also, with your rep your union rep and then from there you discipline them.

Researcher: OK. OK. Thank you very much. So my first question was: what are present practices amongst unit manager. Now I want to direct it directly to you as a person. Seeing that you are the unit manager of your unit, how do you practice presence in your unit when it comes to your patients as well as to your subordinance?

Participant 4: When it comes to let me start with my subordinates, like I I have explained earlier I started with, I started with, I don't know if I'm on the right channel you will tell me but that one and I spoke about is orientation as delegation and supporting my staff and supervising them then after and then we go to the to the very last one. You cannot orientate people, you cannot give people performance agreements and engage with them with with those things and then you do not assess them at the end. So at the end of everything you sit with them, you assess them performance assessment and then you give them feedback, you you let them assess themselves, you also assess them, you give them feedback, then on what to improve and you also motivate your staff and the other. The most important thing is at times there will be gaps when you assess your staff, you see that there are gaps you need to attend to those gaps, and you need to to develop your staff to bridge the gaps. So that's basically that's what I'm doing. That's what I'm doing and we are we are talking about nursing strategy at the moment. I don't know if you've heard about it. Are you from the state. OK, you not from the state, we're talking about nursing strategy, but I think it's all over there just where we are saying we are restoring back the dignity of nursing. So that is our main thing at the moment that we are doing, so it goes with staffing, how you utilize your staff and then also the use of digital access and then the other thing will be training your staff and then yeah, it's all those things.

Researcher: And as towards your patients in the unit?

Participant 4: The patients, you need to as a unit manager. You need to have, I have a relationship with my parents at what they don't know they don't even know that I'm a unit manager but what I'm supposed to be doing is that I need to be introducing myself, that I am the unit manager and this is the service that you will be getting and then I also get feedback from them, are they getting the service from the staff and the other thing will be that one of complaints and compliments, usually you give, I give them questionnaires to evaluate us and if they are thing, if they have concerns or if they have complaints. Usually, I sit with them or address those complaints and they will tell me if they want to elevate them because you start at this level where you you can try to solve their problems, but if you cannot then they can actually elevate it. So you also guide them through the steps that if you are not happy then you can actually elevate this matter. And we also with with parents, I'll, I'll be talking about parents because our babies don't even see what is happening. So with parents, also, as is about that and you also teach them about their responsibilities because it's not only what we are doing to them, they should also know their responsibilities.

Researcher: OK. Thank you very much. OK. So as a unit manager, how do you practice relational care as well as human connectedness with your subordinance in your unit? Just how are you, how are you building relationship and that connectedness between you and your subordinance?

Participant 4: OK, it's it's this one of motivation of staff. Usually, they're not getting incentives. They're not getting incentives. So you try to motivate them. The other day I issued acknowledgement letters and you try to approach them and, in every respect or way and you accommodate them because they are human beings, they will be having needs. You, don't you? You don't need to be stereotype when they request leave and when they request things ,then you are just like this, there are times when you need to accommodate, accommodate them and then when you do things like quality improvement plans for the unit you sit with them, you identify the challenges that you are having, you prioritize them with them, because if you don't involve them, it means you're not, you're not building relationships and then if they are not part of what you are doing, they're going to call it your thing. So you try to to engage them with many things that you are doing, you engage them, you get feedback from them.

Researcher: You see, it's not that difficult. That's why I'm trying to make it so relaxed and I don't stress you doing very well. So as a leader in in your capacity, you are a leader. So how does your leadership role affect you when you want to, you know, practice presence. Your leadership role as a leader, how does it impact, how does it influence or affect you practising presence? Let's go to another one whilst you are thinking about that one. So we've spoken about presence all the time, So what is the advantages for nurses and for even you as a manager, why is it so important? What is the advantages of practising presence in your unit when it comes to the patients?

Participant 4: Are we talking about evidence based practice here?

Researcher: We talk about presence being there, what Impact being there physically, spiritually, emotionally being there for your patients in the hospital? What is the advantages if we as nurses or you as nurses as a unit manager. What is the advantages should you do that towards your patients.

Participant 4: With patients, remember, with us we get patients from Lesotho from North West, from all these areas far areas, Northern Cape not North West. Now they don't have any one they don't have family. So if you are present, you are supporting them. If you are, you're present, usually when when, when you are there for them they are able to vent out their frustrations, they are encouraged by our presence. So that yields positive results even for the babies with us if the mothers don't feel supported usually they won't be producing milk for the babies, so with all those, all the support that you are giving them, they become relaxed, they they they tend to learn how to care for their babies and you won't even struggle with milk production. So I believe that they need that support more, especially because you know better than them, and they don't even have family to support them.

Researcher: OK, so if that is the advantages, what could then be the disadvantages? If you as a unit manager as well as your staff, your sub ordinance if they do not practice presence, what would how will that affect the baby as well as the mother in your unit.

Participant 4: Yeah, the mothers well if we don't practice presence. They they are also not supported, they they don't support their babies and they feel frustrated. They don't know who to talk to. Therefore, you'll be having this, this, I don't want to say bitter mothers that you will behave in these mothers that are not active in their babies care that are not participating whenever you need them to participate, and that also indirectly affects the baby. Because they need their moms to be there for them. So if you cannot be there for their parents, then it means they won't be able to be there for the babies. And usually, if if if we are not present, there is this thing, there is this gap, there will be a gap between us and them and yeah, what can I say?

Researcher: So, what is your role as a unit manager. I'm trying to get back to the leadership one, so we'll break it up in two. So as from day-to-day, what is your role as a unit manager? Not only I'm not talking just now about presence towards your subordinance, but in general, what is your what is your role, what is expected from you in your position?

Participant 4: In my position, what is expected from me like in the morning when I get when I get here I need to check that we have everyone is has reported on duty. If we have those who have who called that will be coming on duty. So, you need to see that you are allocating everyone you are balancing. So, for service delivery and then the other thing that we will be checking as you a unit manager is the drug control, the emergency control because you don't want to be when you want to resuscitate and then there are no equipment, there are no items there in the emergency trolley. That is one of the things that we check and we also ensure that our patience for the day we plan for the day. So with delegation, you'll be seeing you you will be trying to achieve that your patients get the care, all the patients get the care that they, they they they need that is your delegation. Then when you do your your delegation you need now to check if this person really is qualified or is skilled enough to care for that baby. So that's how you do your delegation. Usually, we don't even do delegation we supervise. They do delegation wena you just see if the the delegation that is done is fair to everyone.

Researcher: OK? Any other things that you are doing like in just an example administrative and other things that you need to other commitments besides working physically in the world, what other commitments do you have as a unit manager?

Participant 4: And other one should be attending meetings. The other ones would be ensuring that the off duties you actually yeah that you have planned, leave plans, off duties, everything that that's the way it's supposed to be. Like I was busy with overtime now I don't have a clerk, she's on leave so I need to make sure that the overtime is captured, so those are things that you do if you don't have a clerk and then the other one will be with us in neonatal and will also prepare for for the province. We also the reports that we are submitting to the province. There are reports state we are even submitting to the facility that is admin part of it.

Researcher: So now having all these other duties except working in the ward as a unit manager, being there, so how does that influence that role of that admin and meetings and stuff? How does it what impact does that have actually on you practising presence not only for your patients, but as well as for your subordinates.

Participant 4: With with all those things that you will be doing, like maybe you are compiling reports for clinical governance as you go through the complaint as you go through the compliment as you go through everything that you will be reporting on those things, I believe those are the ones that will teach you then that I need to be there for my staff, I need to be there for my, for, for, for my patients also you when when you are exposed to those things at the management level then that's only when you will be able to see now I need to come down and support these things. We are talking about, there's a time when they were talking about priorities things that are priorities. If you are not part of the management, at times you won't even know what they are talking about when you are there. Now, when you come in the unit now you see that OK, when you talk about staff attitude, actually this is what we are talking about. Now I need to make sure that we bridge the gap. Initially I think I don't know that's my my, my my thinking. We used to staff used to, to, to talk anyhow with the patient, but now it's no longer a case. Now you you are you, you should be accountable. You need to be disciplined. You cannot know that if you are not in that level and now, now that we are talking about the nursing strategy, we are moving that thing not to be only known by managers. It should only come to the staff, so it will also influence their presence I believe and their patients and their patients.

Researcher: Okay, so having all that rolls and acknowledging that you need to be when you see it all they look at all those reports and you acknowledge that I really need to be there for my sub ordinance. I really need to be there for my patients. My question now. Is it possible? How is it possible? Is there any impact? Any influence? Is there anything that will held you from actually being there?

Participant 4: That will prevent me from being there. Ah Ah, there isn’t, I don't think there's anything that can prevent me from being present. One, one of the things from where I was before I came here. We we were not there I think I wasn't there for my patients, for my staff because I was forever attending meetings. So now with removing OPM from this many meetings that the managerial, those that can be actually attended by the Exco. Now, if the OPM's are also attending all those meetings, it means they are not physically in the units, they don't even know what is happening in the unit. So with us here we don't attend all the meetings so we have enough time to be with our patients to be with the staff. So I don't at this moment I don't see anything preventing me from being there for my staff.

Researcher: OK. Thank you. So lastly, I would like to know. With this that you are you mentioned that with challenges and providing support for your subordinance and for your patients in the ward, do you as a unit manager get the necessary support and guidance that you need to execute your duties, your leadership role, as well as being present.

Participant 4: That's a tough one. That's a tough one. That’s a very confidential one. Yeah, I don't think personally, I don't think unit managers are getting enough support. I started somewhere else as a unit manager and you are thrown, you are thrown, I don't know at the deep end because there's no orientation like I'm saying I need to orientate my staff, but I don't get orientation. Now the meetings that I used to attend, they were using this big language. They were using these big terms and this abbreviations that I didn't even understand. I even had to when I left the facility, I had to draw a train orientation program for OPM's because I felt that new OPM's are not supported, so I don't think there is enough support for OPM. So I just told you need to do this and this and this and this. And remember, you were just a professional nurse now you are a OPM, you are not even guided and you are expected to care for, I think they can do better. I think they can do better. But I think with this nursing strategy thing, because it's also about leadership, supporting leadership, I think maybe we are getting some where.

Researcher: Okay, so besides not getting the necessary support. Is there anything else that is like a challenge for you not being able to actually execute your duties? Actually being there for your subordinance as well as for your patients?

Participant 4: The other challenge that I would I would say I am facing is the consumables. Shortage of consumables we don't have consumables, so at times I'll be running going to the warehouse, checking all those things when I'm supposed to be supporting here. This is me now being a supply chain officer, checking when are we getting this? What is happening with this and this and this and this. So we have a serious challenge of consumables. It is time consuming because at times if you don't go to supply chain, you have to sit here and phone your colleagues from other hospitals.

Researcher: OK. Thank you very, very much. I really, really appreciated you did well if I have any follow up questions is it fine if I contact you just for a few questions, if there's any. OK. Thank you very, very much. Is there anything else that you feel that?

Participant 4: You are welcome.

Researcher: Maybe I didn't cover or didn't ask that you would like to share with me.

Participant 4: No. Don't think there is.

Researcher: OK if you can think about something you've got my cell phone number, I'll leave it with you and you can contact me. Thank you very much. Once again. Bye.

Female; Age-44; 2 yrs of unit manager

**Participant 5:**

Good morning, ma'am. My name is Bernadine Smith and I am a Master’s student at the Northwest University. I'm also a registered nurse. OK, thank you very much for taking your time to answer a few questions. I really appreciate it. OK, so my research like we discussed its about present practices amongst the unit managers in a selected hospital here in the Free State province. First, I'd like to say that there's no right or wrong question. It's just your perspective that counts. If there's a question that you feel that you are not comfortable with answering, you are more than welcome to say you prefer not to answer it. If there's anything that you maybe remember on the later stage that you'd like to add to, please feel free to call me. I just want to know if it's OK if I maybe I follow up questions one or two few questions if I can contact you on a later just to get the information, yes. OK. OK. Thank you very much. Is it OK if I record the session? You give permission. Thank you very, very much. I promise I won't take up a lot of your time, minimum. 30 minutes. If it goes over, it's because we're having a nice conversation. OK, so it's a broad topic. The first question is very broad. So what I just want to know. In general. If you look at you as an operational manager, unit manager, what do you think are the present practices for a unit managers amongst unit managers? Present practices. Hmm. Can you simplify your question maybe or elaborate further. OK. So talking about presence, meaning that as a unit manager like I'm saying it's a broad question. What are your the present practices? How do unit managers practice presence currently?

Participant 5: It starts in the morning. I'm here with them when they take report and then whatever mistakes that I pick up during report taking, then we use it as a learning opportunity. Either we do it on the spot or when we share the report later, before, after praying. The other thing that I normally do for the ward we have scheduled meetings, where we will discuss the topics that I've already planned prior, the meeting and the ones that they need to be added. So I'm one person who doesn't take decision on my own. I involve them despite me having my own vision so but I always like to make them part of the decision making. So I give them opportunity if it fails, then I'll say to them now it's time it's my turn to practice what was in my mind. So that's normally how I do it. When it comes to the patients, every Thursday I have, this is pediatric ward neh, I have interviews, dialogue and meetings with lodger mothers. Where I would I normally want to find out if the doctors are involved in them in the decision making. Are they up to date with why are they here? On daily basis, the doctors would run tests or test or assessment. Then I would ask them did the doctor tell you why did they do 123 and four? And then you would write it in the report so that we have it down that the doctors are communicating with the mothers where there's lack of knowledge, then we intervene at times it's language barrier, so that's where we intervene. But up to so far, our doctors are trying their best. So where I pick up problems then I have a meeting with the doctors to say this we have 3 disciplines in this discipline. 1234 is lacking. Can you please improve on 1/2? And then when it comes to the religion or spiritual upliftment in the morning, when we sing and pray, we are together, and then there's a local preacher by the name of ntate John, whenever we have one who's struggling like out of with personal problems or not taking in what the doctors have just said to them, I normally invite our local preacher to come and have one-on-one sessions prayer sessions with them and he you would even go to extend of coming voluntarily to check if there's anyone that needs prayers. We also involve our social worker. Whenever we see social problems, we take it upon ourselves. At times, we do involve the doctors, but just to let them know that there's a mother like 1234, the initial consultation of social workers, we can do it ourselves without even notifying the doctors.

Yes, thank you very much you doing a great job.

Participant 5: Thank you.

Ohh, when you talk about presence I just want to pick your mind. I just want to find, I've briefly explained my version of what I think presence is. Can you please elaborate what do you think if you have to define presence in your mind, what is it?

Participant 5: Presence is always being there for staff, for our mothers, for our patients and even for the Community. There’s times come and ask questions and you just help out with the information that you have. Presence is being there for the staff on a personal level, we are here, but we have problems other than our job related problems so if you are present not digging too much, but the little that you can get make a referral for those who need your help. Can be financial, you can just give advice. Can be emotional, if I am unable to support emotionally, I can refer because we have EAP here in hospital, so I can refer. A healthy staff a productive staff. So that's what presence is you need to know each and everything that is happening in your ward. You need to be there to help, not to to to know and to take a backseat. You need to be involved at all times. Nah, I always tell them you'll only get me in the office when there's work office related work that I need to do, but most of the time I'm amongst I'm amongst them to see whether do they need my help. At times you will say do 1234 and they are afraid to say to you. But how do we do it? So for me to see that they are doing it the right way or they are struggling is for me to be present at all times so.

So you just mentioned your office duties that you're having, which is like your documentation or whatever you have to do. How does that impact? Does it impact you on being present inside for your patients and your subordinance. Participant 5: No, it doesn't affect me. If you can look there. I have my weekly program, so I know on these days these are the things that I'm supposed to do. So in between if it's it's a dire need for me to be in the ward, I will just leave it and go and attend to whatever and then come back. So I stick to this. Hence you'll find that I'm done with whatever that was planned for the day so then I have time for them, them meaning staff and lodger mothers and patients. So I stick to, so I can't go wrong with weekly plan.

How do you as a unit manager? Practice like that relationship, building relational care and that human connectedness between you and your subordinance? Participant 5: OK? What I normally do every end of the year we would have a one on one like where I would call them, like tell them wena your performance is like this, praise and wabona. And then where they are? Where I need to put more effort then I'll tell them but mostly I do it just to to encourage them. At the end of the year and then I give them opportunity to say wena you are like 1234 and then it's your turn tell me where can I improve? Can I keep whatever that I'm doing? Are you happy with whatever that I'm doing? So I do it yearly because you will think that you are doing the right thing at the end of the day you are not. And for me to fix whatever that I've picked up from them, it's it's when I hear it from one person, more than one person, then I know it's a problem. There's no way three people can say the same things at once. So then I work on whatever that they've highlighted to say this is a problem for us, so I do it every year at the end of the year. That's where we sit and and one-on-one and then we have our recreational trips. At times they do include me when I'm not busy, I would go with them and then at times they do it on their own. And they do it more often and I like it because they come back united. So I often often even encourage them to do it. We can't be here. Remember here in in government hospitals, we don't have team buildings. So recreational it also serves as. During Heritage Day we would share responsibilities, wena you’ll will cook this wena you will cook this and then at the end of the day we sat down and share the meal and talk. About everything, the one that I have just said, yeah, at the end of the year with sit down, we also include a private achievements to say for this year I have achieved 1234 and then next year I'm still going to work on 1234. So that's what we normally do at the end of the year in this ward, Thank you.

So tell me, is a a leader having so as a unit manager having so many roles like your leadership role, your leadership responsibility? Do you think that as a leader? In your ward, in your hospital, you think that could affect or impact on you being present in your unit for your patients and for your, for your staff? And if so, how?

Participant 5: Me being involved in other. Do you mean that?

That. No, I mean it's you are you are the unit manager. So obviously you are a leader. So you've got your leadership roles, OK, other commitments, meetings, whatever. Do you think those roles, how does it affect you being present? Participant 5: They don't because with meetings we have prior notifications. So you know already on this day I'll be having a meeting. So I'll have as minimal a commitment in my ward as much as possible. So it doesn't. It doesn't. Because really, as I've said to you, I have my own schedule that I follow and we have a list of meetings that are provided to us on time. So you can plan around your scheduled meetings and weekly plans.

Knowing now what presence, what that we are talking about, what do you think is the advantages when nurses as well as you as the managers? What’s the advantages of you guys practising presence? What does it mean? To everybody else, if you as a nurse, you as a manager practice presence in the unit.

Participant 5: I think it helps with misunderstandings. Because you have. You attend to them in time, whatever that is happening in the ward when you are present. You can pick it up early and deal with it before it could even cause problems, like for instance nna, I am present. I have my own routine like I am saying I'll be taking report with them and use the opportunity when I pick up problems, then I use it already as as a learning opportunity. Me again taking the report also helps me when the doctors come, I can advocate for my patient. If you are not taking the report, if you are not involved, people will come from outside, ask questions., then you are clueless because wena you are not part of the team. So that's what I normally teach them for, they must be inquisitive, they must ask question, they must be present. They mustn’t shy away when they do not know they must ask if I do not know mina I'll invite other people relevant stakeholders to come and help all of us. So we are honest with I've built that thing of us being honest with each other of asking for help if you don't know, but you know it's not always where things will run smooth. There will be one or two who will take their own negative way? But you concentrate on these positive ones and then at the end of the day the ones that were not in end up giving up and joining. So while I like again we have different roles, we have different job descriptions. But what I like about the nurses in this ward, they are present at all and you'll never hear them saying this is not my my scope. They take each and everything as a learning opportunity. The only thing that the assistant nurses of this ward can't do it's administration of medication. But As for the rest, they can admit, they can receive patients from theatre. You can leave them with staff nurse, you'll find the ward in order. So that's what I I'm sure that's what they've learned from me. I'm always there to help, even if the sisters are there. So if you are a leader and then you lead by example, it's easy for your followers to do the same. So, it's helping us a lot. They are so independent. I don't want to lie.

What other advantages are there when it comes to the health of the of the of the patients when nurses are present.

Participant 5: For me we do have, we have less adverse events because they are always there and you can be there, but if you don't know what to do, it's still a problem. So they are there and they know what to do. So we have less adverse events. Because whatever that they pick up, either they act if it's beyond them, then they report to the their to the supervisor of that shift. So it helps in that way, that our problems our, patients don't have complications because of early detection of of whatever patient is presenting with at that time.

There's something that you also mentioned about at times there might be some of the subordinates that might be a bit negative. So as your role, what do you do to rope them in to try and change their perspective to join the others that are more positive?

Participant 5: OK. Then I also do a one on one interviews to find out what is it? Why are they not part of the team? Then I'll work on whatever we have discussed and I won't make decision on my own. I will also ask what are you, what are your expectations of me? How can I help you? So based on whatever that they've listed, then we'll work on that. Failing, which I'll also involve my assistant manager to come and step in and then, as I've mentioned earlier, we also have EAP. Because it's not, it's not always where a person will be existent resistant for no reason. At times they have personal problems that is difficult for them to discuss with me. At times they don't trust you as a leader. So for them to open up, you need to sit down with them and talk and find out. Try to establish what where the problem is and work on.

It OK. Thank you very much. We've spoken about the advantages. Now, all of all the things that you've listed previously was about what the advantages is when nurses and the unit managers are practising presence. So what is the opposite? What will then the disadvantages be should nurses not practice presence in the ward? Should you, as a manager, what can go wrong if you guys do not practice presents?

Participant 5: Still the same opposite of what I've just mentioned, one patient will complicate, two you won't identify the problems on time. Three, there won't be unity in the ward and the other thing is you won't know your staff better, you won’t know where they are lacking. So I think those are some of the negatives.

OK. So as a unit manager you have this huge role on your shoulders being a leader. A unit manager and as well as being part of your team physically there, guiding them, showing them the ropes. Are use when you are sometimes faced with challenges. Do you have the necessary support and how are you supported when you are faced with these challenges?

Participant 5: Yes, I do have the necessary support neh from the sisters, from my assistant manager and from nurse manager. From the professional nurses, maybe they are here when I'm not here on weekends. They are here after hours and I'm not here. So whatever that they come across, they will sort the problems out and then nna in the morning, I'll just get the report to say yesterday we had 1234 but this is how we dealt with it. At times they would even phone me at home to ask for advice. Is it fine if we do it like this? So I've I've also told them to, they must feel free to make mistakes as long as it's not affecting the patient's health. So you know, today it's a mistake, but tomorrow you’ll be fine? So that's the support I get from them. And also from the whole team I get the support because that whenever I ask them for guys we have performed badly in our audit results. Let us sit down and discuss what went wrong the following month, then you get you can see or there's a change. At least we sat down and discussed everything and came up with solution and then our audit results will improve, so that's the support I get from from the ward. And then from my assistant manager I'm only two years as unit manager so where there are challenges I would go to my assistant manager to say; How can I tackle this problem? Then she will sit down with me and say do 1234 and then I'll come back practice whatever that I was told to do and then give feedback to say whatever that you have assisted me with has worked. As much as matron (name withheld), I had a problem in this ward with one of the difficult professional nurses, so it went to extend where Matron (name withheld) could see I’m not fine and end up calling me and said you mustn't look like your problems as much as this is a learning opportunity. So whatever that you do, we are not always right. There's somewhere where we are wrong as operational managers and she said to me, but that doesn't mean you must stop disciplining the staff. So pick up your socks and do what you are here for so we do, me nna personally, I do get support from the two ladies that I've mentioned my assistant manager and my nurse manager, recently just they've supported me fully. I don't want to lie.

That's great. That's great. Yes, and that is it for me. Is there anything that you feel that I haven't covered or under this topic that you would like to add? Participant 5: I just want to add. Being present, you need to be present. Remember we have PDMS where you monitor the performance of your staff so that at the end or we do it twice, biannually you need to sit down and assess them. So how will you do it if you are not present, you won't know that this one is performing this way and then this one is performing. So, through me being present, one of the advantages is it makes it easy for you when you sit down to do their performance assessments.

OK. Thank you very, very much matron. I really appreciate your taking your time. And like I said, if there's any more or follow up questions that I maybe missed I will come back to you and thank you for allowing me to do that, I really appreciate that.

Participant 5: Hope I was of help.

Big, big help. Big big help. Thank you very much. OK.

Participant 5: OK. Thank you.

Female; Age- 48 yrs; 2 yrs unit manager

**Participant 6:**

[Researcher: Thank you very much, ma'am. Matron. I really, really appreciate you taking your time to answer few of my questions. OK, so my name is Bernadine Smith and I am a Masters student nursing student at the Northwest University. I really appreciate it, and if there's anything that you would like to add, if there's any questions, please feel free to ask any questions. If there's questions that you feel that you would rather not answer, it's fine with me. You don't have to answer the question. I just want to ask if possible, if I maybe have follow up questions that I didn't ask during the interview, is it fine if I can come back again on a later stage? Thank you very much ma’am. And then I also want to ask permission. Is it OK if we record this session? OK. OK. Thank you very, very much. OK. So let's start. So like I said previously, my study is about present practices amongst the unit managers in this specific hospital. So it's a very broad question. My first question, what do you think are the present practices amongst unit managers?](https://livenmmuac-my.sharepoint.com/personal/s219266212_mandela_ac_za/Documents/Transcribed%20Files/Part%206.%20Univer.m4a)

[Participant: I think the present practices among the the unit managers. We are we are constantly, you know, focusing on the good governance of the units. Making sure that everybody, SOP’S and all that and everything is in place not only in place, but people are aware and they are signed. We are forever, you know, reminded about the importance of, of, of, of remembering our national nursing strategy. So it's more of being compliant, being on the on the, on the, on the good standing with your managers, you know. Being being there for our employees, yes, we are trying, but we are more concerned on the side of being my unit has to be percent compliant. We have to be sure that we are submitted on time. We have to show that everything is being done according to how it's supposed to be? Yes. So we are more of you know, SOP guideline, goals, objectives and orientated type of yeah, that presence that how that's how I can explain it.](https://livenmmuac-my.sharepoint.com/personal/s219266212_mandela_ac_za/Documents/Transcribed%20Files/Part%206.%20Univer.m4a)

[Researcher: OK so. You've given me a broad perspective of what you think the presence are amongst you’ll as managers. Thank you very much for that. Now I want you to, if you should be able to define presence, how will you define presence? What do you understand by that specific word presence?](https://livenmmuac-my.sharepoint.com/personal/s219266212_mandela_ac_za/Documents/Transcribed%20Files/Part%206.%20Univer.m4a)

[Participant 6: Yeah. For me, presence means being visible being with your team. Being with supporting them all the way, because most of our nurses or most of our professionals who are coming to the OPD's, we find that they they have been working somewhere but they were genius, if I put it that way. But if they come here, they're exposed to being taken the charge ship of the clinic. Seeing that things are processes are running and everything. So my presence being visible being the to guide them to support them and to applaud them, even if they made a mistake to say no, this is fine, let's do it again. You will be better the second day because the first time that you are in the limelight, you've been under, somebody in the ward but now here you are the leader, you are in front. So being for me is is being with them and seeing that because there's many clinics that I'm looking after that and they're different. The other one is different, they're not the same at all. So it's for me overseeing this process is running well, she's now, she is, she understand what's expected of her and going to the next one just like that. That's for me, simply being present for them and helping them to be also make their things on time because we have to have timelines here. We don't do things forever. If we have said KPI must be in by 2:30 today, we must all you must comply.](https://livenmmuac-my.sharepoint.com/personal/s219266212_mandela_ac_za/Documents/Transcribed%20Files/Part%206.%20Univer.m4a)

[Researcher: OK, so you've already touched on my next questions that that that I wanted to ask on. How are you as a unit manager present in your unit? So you already explain how you are present for your staff. So how are you present for your patients in the in the, in the, in the unit?](https://livenmmuac-my.sharepoint.com/personal/s219266212_mandela_ac_za/Documents/Transcribed%20Files/Part%206.%20Univer.m4a)

[Participant 6: We we must remember, we we we are having about 5 clinics here which we are seeing about per clinic average of 25 patients follow-ups and maybe at a maximum 10 patients in In-patients. And having having to have to visitors every day, it means that we have to speak to everyone as if you know this the first time that I see you doing that tomorrow you will be like the other person, you won't be here tomorrow again. So for me being present for the patient means that I must see to it that they are protected in terms of we nurses complaining to our practices and what is expected of us. For them to know me that this is a matron this is a leader here if there’s anything, I'm not happy here, she is around us, I know where the office. That's for me being present. Knowing my face and me, knowing them of course it's difficult to know them, but at least I must say, ma’am I saw you last time. How was the experience and how are you today? So that for me that's has been present for my patients. And to ensure that they know their rights, of course they know they will know their rights even know your responsibility, but they know their rights, yeah.](https://livenmmuac-my.sharepoint.com/personal/s219266212_mandela_ac_za/Documents/Transcribed%20Files/Part%206.%20Univer.m4a)

[Researcher: Thank you for that. So tell me, how do you as a unit manager in your unit practice like relational care and that human connectedness, you know, with your sub ordinance?](https://livenmmuac-my.sharepoint.com/personal/s219266212_mandela_ac_za/Documents/Transcribed%20Files/Part%206.%20Univer.m4a)

[Participant: Yeah, it's a difficult one. We are dealing with staff members who are on the edge, they're they're here, anything, they just know catch fire, anything, any small comment? My supervisor told me that our files in OPD are more than any other place he has ever worked in and it's it's a male. He said mme I've never seen women that are fighting like this once in OPD. So, it's it's very it's very it's very draining is very stressful to try to balance because remember each and everyone who comes here crying I must sit down and listen. I must sit down and say ma’am are you ready to put this in writing or want us to solve it you know, informally, just call a person to sit here because when the things sit in my office, I always tell them that It's no longer a gossip. I have to attend to it. If you come to me, then really it's not between us now. I have to interview that person because I don't want to be troubled at the workplace. One of the responsibilities is to to to ensure that all the conflicts are resolved so that the key environment is very friendly to all of us. So it's very it's very it's very it's very straining to try to keep to try to keep up with their fight, is very straining to to try to keep up to say let's have a team because somebody have done something to and they take it personal. Don't ever think that it's work thing and it end here it goes on and on and on and on, I know they. The surgical team mme they've having a committee team but they call it team building committee, we we we see people connecting there but after somebody come to work they differ as if they never seen each other again. So it's it's it's it's not easy at this point in time and with the people that were leading to keep up with their stress levels at home, where they bring it to work and to keep up with whatever the the work the work environment is very stressful because there’s a lot of things going on here now, if the person is also heavier burden that cannot be compartmentalized. If you if you understand me well, then it can be a mess. So it's not easy to keep up with all those demands as a leader, you end up saying you know what the other one does said to me i’m resigning and I said please resign and the next day she brought me a letter. My supervisor called her after two days and say no you have to come back because, I was tired and that's not how it’s supposed to to be done. I said please resign, I will receive it, so it’s not easy. Even if you try hard to move them to swim with them and to support them, but at times you feel like you know I'm done.](https://livenmmuac-my.sharepoint.com/personal/s219266212_mandela_ac_za/Documents/Transcribed%20Files/Part%206.%20Univer.m4a)

[Researcher: Yes, everybody reach a stage in life where you feel it now. OK, So, what are most of the reasons why they are fighting?](https://livenmmuac-my.sharepoint.com/personal/s219266212_mandela_ac_za/Documents/Transcribed%20Files/Part%206.%20Univer.m4a)

[Participant: It will be like, oh, the other the other others are so personal that they have nothing to do with their work. They've got nothing to do with their work. I remember the case that we are still busy with his between his sister and a staff nurse. They called me that morning to say they said, you know, they didn't call. Me a household aid called me to say they the enrolled nurse was rude to her and I came in and we sorted out the matter, even the enrolled nurse was apologizing saying I'm so sorry if I was ugly with you, that was not intentional. But then when I left the clinic, the sister started or the enrolled nurse started or I don't know who started, but they were telling they were insulting each other very badly. Now we are busy charging they are charged. We are busy with their process. And the other one I don't know whether it's being bullying or what, but every time you know we have been this thing of saying our clinics don't run every day. If your clinic is not running, if maybe you are two nurses in the clinic we have to exchange to say this one is going out and this week will be that looks up that type of rotation, but this particular one will always have the excuse of not going out. It means that the other one must be we said no, no, no it can’t be. So, such things they, they, they they bring about, you know, sour sour environment with the environment. But if you look at it, you know, if it out-of-the-box you see this person said you're from from somewhere, but now it is displacing this anger or the frustration in the workplace. So, there are little things that are happening. There was also a fight. We had a meeting and I made a comment about the incompleteness of the emergency trolley. Guess what? When we left it, that sister was saying to me matron, this the last time that you are talking about me in the meeting. It’s a misconduct, she’s been charged because, how do you stop me from doing my work? If I comment to say that I'm not happy about how the emergency trolley is being checked, it was incomplete, she felt that I was, I'm on her case. Such things, you can't say they are not, they do not want to be corrected. They don’t want to be confronted, you know, they feel that if you are want things to be done as they're supposed to be done, you're on them, on their case. So, that's what I'm saying people are just on the edge. If you say a thing, no matter how in what context they're just on fire day. We had a case yesterday just between the nurse and the that clinic in charge. Someone was sitting here. I was sitting that side. Now we're trying to find out that this nurse is is crying, that the sister is on top of her. The sister is not treating her well but we wanted to find out what happened, what did she said? But we could not find anything serious red flag to say, but sister you did it you were very rude or you were not, you ugly you ugly with her why, we could not. We end up saying, you know we we think that we need to refer you to EAP. Because, even, we've been taking out of that place, but we need EAP. She might be having things that are troubling her so much, but she just need she's just spacing everything to the sister. But instead, well, everything that that happened was reasonable, and it was within their right of the sister to correct things to say that this is how we're doing things. We cannot do these things this way. So, this is the type of fight that they are having, type of you know, time and again there is a case we have to sit down for things that we feel that these people are very rude, they rude. So, but we do have to check things that we can’t ignore. That we have to sit down and we have to resolve them. To say, but we, we are supporting you don't resign or don't drink. Because a lot of things came out yesterday, she's not in a good state. Just to find out, she's not drinking. We said OK we'll take her out of that place, we are putting in another clinic, but while we're doing that, we support you and motivate.](https://livenmmuac-my.sharepoint.com/personal/s219266212_mandela_ac_za/Documents/Transcribed%20Files/Part%206.%20Univer.m4a)

[Researcher: Thank you matron. How does you as a leader in your position obviously you have a leadership role, with other commitments and like meetings and administrative roles, so is your role as a leader, do you think that role has an impact or affects you being present in your unit for your staff and for your, for the patients and how do you think does it affect you?](https://livenmmuac-my.sharepoint.com/personal/s219266212_mandela_ac_za/Documents/Transcribed%20Files/Part%206.%20Univer.m4a)

[Participant 6: I'm overseeing about 8 clinics and they are scattered like hematology outside of the university and we having gastro up there, we have genetics on the 5^th^ floor, then this group here. So, most of the dermatologist also their department is that side of the university, although the clinic is this side. Most of the clinics prefer to have their own departmental meetings and they also prefer the matron must be there because there are some other issues that needs me to respond or to give information or to take over to say I’m going to inquire or bring back the feedback. So, it is impacting on the visibility on the, on the, on the platform, in terms of the patients and other clinics. When you invited to the specific clinic, their meeting might take an hour. Afterwards you need something that you need to do in the office. Then it means that for a for the first three hours of your morning, nobody saw you. You just reporting to say I'm here, but I'm going somewhere, if you need me please WhatsApp me. So, it is really affecting, it is affecting, yes. But hey, on the other hand, I would like to say also is important for me to be also be part of those meetings, because there are real issues that needs the head or the leader to say something or to help or to support. So, I don't know how best can one balance the two, but I think that it is important. It is important that I attend those meetings.](https://livenmmuac-my.sharepoint.com/personal/s219266212_mandela_ac_za/Documents/Transcribed%20Files/Part%206.%20Univer.m4a)

[Researcher: OK. So talking about support when you are conflicted and have all these challenges with all the staff here always on the edge, always fighting and you said that sometimes it becomes too much for you. How are you being supported in this matter to try and sort such things out?](https://livenmmuac-my.sharepoint.com/personal/s219266212_mandela_ac_za/Documents/Transcribed%20Files/Part%206.%20Univer.m4a)

[Participant 6: My supervisor is very supportive. He is very supportive. I will, I will just go to who's office and phone to say, can I come? I need to you to help me with this or I’m having this issue then he use to tell me try to help me to navigate or to say this is how we'll do it or just leave it to me. Give it everything to me I will handle it, it’s a bit complicated. You know he's very supportive, really I must say. We never feel that we are left at the at the deepest end or alone, no he is always there they are supporting us, yes.](https://livenmmuac-my.sharepoint.com/personal/s219266212_mandela_ac_za/Documents/Transcribed%20Files/Part%206.%20Univer.m4a)

[Researcher: It's nice. OK so. What are the advantages re even nurses and yourself, practice presence? What can people gain out of it if nurses as well as the unit manager practice their presence in the unit with the patients?](https://livenmmuac-my.sharepoint.com/personal/s219266212_mandela_ac_za/Documents/Transcribed%20Files/Part%206.%20Univer.m4a)

[Participant 6: I think we'll have less, less conflict in the in the workplace and the other thing is that people will tend to know each other better. I always see you with the patient and I always see you being stressful, but I always want to see you when you are relaxed as a person, I want to see you when you are in a good mood, you know when there's nothing we want to see. Maybe we are just missing out something that when you are stressful, you are ugly person, but actually you are a very nice person. So, I think it it it it will benefit all of us, if we can have know, if I understood your question, I'm still answering you well.](https://livenmmuac-my.sharepoint.com/personal/s219266212_mandela_ac_za/Documents/Transcribed%20Files/Part%206.%20Univer.m4a)

[Researcher: You on the right track, yes. How would it benefit the patients, their health, their overall health and then being in here in the hospital? How how will it benefit them if to, what advantage will it have if we practice presence?](https://livenmmuac-my.sharepoint.com/personal/s219266212_mandela_ac_za/Documents/Transcribed%20Files/Part%206.%20Univer.m4a)

[Participant 6: Yeah, yeah, if we are, we are there for each other. No, I think even the the complaints. The patient complaints will be minimized because, but hey, I must say for the past few months I've seen decline in people coming here being very angry for for the nurses have done this, this and that and that and that. I've seen it less and less I don't know whether they are sorting for themselves or what is going on, but. Yeah. So I think we'll be having less patients that are dissatisfied and we are also behaving less nurses who are also dissatisfied. Because it’s not only nurses who are who are at times very ugly, patient, the patient themselves and their families are also at times ugly with the nurses, the nursing staff. So, with them being presence for patients, for each, for each other, it will minimize such things and the fight between themselves that we are having I think that we are now, I don't know what where we are with that, but it is high, high, very high. We are sitting, we having meetings, having cases for people who are fighting each other in the workspace like never before.](https://livenmmuac-my.sharepoint.com/personal/s219266212_mandela_ac_za/Documents/Transcribed%20Files/Part%206.%20Univer.m4a)

[Researcher: So you said, you did mention it is most of the time, these are the frustrations are more personal than work related.](https://livenmmuac-my.sharepoint.com/personal/s219266212_mandela_ac_za/Documents/Transcribed%20Files/Part%206.%20Univer.m4a)

[Participant 6: But they end up being influencing the attitude in the workspace, you know, in the timeline of a person and you know such things. Researcher: OK, so if a nurses and you as a unit manager, if you are not there present for the patient what could be the disadvantages?](https://livenmmuac-my.sharepoint.com/personal/s219266212_mandela_ac_za/Documents/Transcribed%20Files/Part%206.%20Univer.m4a)

[Participant 6: Yeah, it will be an ugly, ugly space. Look, all of us, we need some sort of supervision. Can you imagine if there's no one who's looking that this patient is being sick because you’ll find at times people seated here. Somebody is having a paper but why, why which clinic are you ma’am? Why are you still having a paper in your hand? No, I’m at the hematology. No, please hand it in because they don't know you are here. They're looking for you. You have to go and report yourself that you are there so that your Dr. can call you. So, we we all need supervising some way or the other. Nurses are going up and down there, they are just going on with their own routine. Nobody looks at the patient and say are you well, there ma’am? Why are you sleeping? Are you good? Can I give you a blanket? You know, they get so much engaged or caught up in the routine that they forget about these people, that they are here. Small things, we’ll find that it's 7:00, somebody's eating pap, pap and milk, whatever. But it's cold food. And you say, but why are you eating, let me warm it up for you? Those are the small things that make difference in patient’s lives. Those are the small things that make them feel your presence. Because they're saying we are all here with our uniform, with our red lips or make ups, but nobody is taking care off that patient who's lying there. Why are you ma’am, don't you want a pillow or don’t you want a blanket? So at least you're comfortable while you are waiting for the doctor? I think that that's that if I'm not here to look after such small things, patient will suffer. This small things that i’m talking about. They are, i would call them suffer medically, but at least feel that somebody take care of me. She said that I'm eating cold food. She wants to warm up food for me. She can see that I'm lying and I'm not comfortable. She's giving me a cushion. She give me a blanket. You know, they they end up feeling accepted and welcomed and that we are pleased to have them here as patients. I might not be doing much because there's a lot of clinics. If you ask me when last did I take blood? I like take blood but I always, I think my eyesight is getting bad after the operation. I always miss the veins I never do it. But I'll always come and greet the patience. Ask them how are you doing? Are you good? Is there anything that you want? Those are the small things that I’m able to do.](https://livenmmuac-my.sharepoint.com/personal/s219266212_mandela_ac_za/Documents/Transcribed%20Files/Part%206.%20Univer.m4a)

[Researcher: If you are not be able to do that, what can what can result from?](https://livenmmuac-my.sharepoint.com/personal/s219266212_mandela_ac_za/Documents/Transcribed%20Files/Part%206.%20Univer.m4a)

[Participant 6: I think they'll be frustrated. They feel that we are here, but we are more burden than as patients. They will feel nobody is taking care off us. It is only Mrs. Van der Merwe please come this way, come that way, please Doctor, want to see you. But Mrs Van der Merwe wants need more than to ask are you good? How are you? OK or are you fine? OK, no I'm good, that's all.](https://livenmmuac-my.sharepoint.com/personal/s219266212_mandela_ac_za/Documents/Transcribed%20Files/Part%206.%20Univer.m4a)

[Researcher: OK, so as a unit manager, what can you do to support or give guidance to your subordinates on those small things that can make the patient feel welcomed and happy?](https://livenmmuac-my.sharepoint.com/personal/s219266212_mandela_ac_za/Documents/Transcribed%20Files/Part%206.%20Univer.m4a)

[Participant 6: Feel welcome? I always fight with, especially these young student Doctors. Because they will come, they never greet patients. They just come maybe they are so on high horses so they don’t greet them the patients they're waiting for you. The nurses please don't call the patients by your name by the name, by their names. At times, they are our mothers, their age groups, our famous age groups, Mrs, Mr, Mme, Ntate, dumelang, lekay, reteng mummy, have a seat here. They are patience or adult me myself too, I don't want to be treated in that awkward way, the same with them? The same with them. So we we, we, we, we are, we are, we are on it to say no doctor here we call patients by their name. And if it happens, I'm not going to speak to you i’ll speak to him. This is your clinic i’m just the supervisor speak to your doctor. Tell them this is how we do things. But teach them to see doctor whenever you call my patients, please use their title or their gender Mme, Ntate, Mr or Mrs.This is how we do things and treat them, as I'm speaking to you now, I was at the back there and I saw a lady standing up, but she nearly fell. I didn't understand what happened, but. I said, oh, Mama. You know I'll jump and I'll fall with you, because I am here I cannot allow you to fall. Only to find out that the tip of her crutch is fading. I said OK I'm quickly going to physiotherapy to get you a tip. The tip of the crutch and I brought somme three. I said i saw three ladies maybe it's the same thing and definitely, the other one said matron, please let me have one, let me have one, you know. So those are the things that we have to take care of our patients holistically. That patient was not there for the crutches. She was here for something, I don't know for which was she, but we ended up attending to other things that might also be a high risk of falling in our presence or even at home causing a fracture which were unnecessary. So those are the things that I, we hope that the the the team will copy from us. That is not about the prescription, it's about also seeing that oh is she fine is her crutch OK, ma'am, is it comfortable? Because you are in hospital, can we order you another one or is the tip OK. So, I'm hoping that they are learning.](https://livenmmuac-my.sharepoint.com/personal/s219266212_mandela_ac_za/Documents/Transcribed%20Files/Part%206.%20Univer.m4a)

[Researcher: Thank you very much. I really appreciate it. Is there anything that you'd like to add, maybe anything that you feel that I didn't ask you during our conversation that you feel that it's important that this needs to be noted as your role pertaining to practicing presence in the unit?](https://livenmmuac-my.sharepoint.com/personal/s219266212_mandela_ac_za/Documents/Transcribed%20Files/Part%206.%20Univer.m4a)

[Participant 6: No, I think we have covered all of the important aspects, yeah. Getting to that to that subject that we're having and I think we've covered most of all of the important things.](https://livenmmuac-my.sharepoint.com/personal/s219266212_mandela_ac_za/Documents/Transcribed%20Files/Part%206.%20Univer.m4a)

[Researcher: Thank you very much and I'm looking forward to giving you the results of this study and if possible, like I can mention if I need anything follow questions, am I welcome?](https://livenmmuac-my.sharepoint.com/personal/s219266212_mandela_ac_za/Documents/Transcribed%20Files/Part%206.%20Univer.m4a)

[Participant 6: Yes. Yes, please. You're welcome. You're studies is going to benefit all of us. Yeah. The studies, because at times we might think that we're doing things right, only to find out that we're missing the point. This is the way we're supposed to be going. So things such as this, you people who are who like reading we can support you.](https://livenmmuac-my.sharepoint.com/personal/s219266212_mandela_ac_za/Documents/Transcribed%20Files/Part%206.%20Univer.m4a)

[Researcher: Thank you. Thank you very much. I appreciate it.](https://livenmmuac-my.sharepoint.com/personal/s219266212_mandela_ac_za/Documents/Transcribed%20Files/Part%206.%20Univer.m4a)

Female; Age 54; 8yrs unit manager

**Participant 07:**

Researcher: Good day sir, my name is Bernardine Smith and I’m a master’s student at the North West University. Thank you very much for affording me the opportunity in asking a few questions. There’s no wrong or right answers, just your perspective that counts and like I mentioned that if there’s a question that you not feel comfortable answering, you can say no. You don’t have a problem if I record this session.

Participant 07: No, I don’t have a problem.

Researcher: Okay. Thank you very much. Is it okay if I maybe have follow-up questions, if I forgot to ask you something, if I can come back at a later stage.

Participant 07: You are very welcome.

Researcher: Thank you, thank you very much. My research is about presence practices amongst the unit managers in a selected hospital in the Free State. So, my first question is very, very, broad, okay and you can answer it however you feel like answering it. So, my first question is like what in general according to your knowledge, what do you think are the presence practices of unit managers.

Participant 07: Presently, it’s so challenging because like, some of the things that, I can’t even, not a problem, for example there’s a shortage of staff, there’s not enough personnel as I indicated, shortage of what you call it, equipment and also absenteeism also affect the things that we manage, some of the things that affects me, yes. And also, I can say, my supervisor also they would wants things to be on time, of which I at times I have to be in the unit not to in the office because people didn’t turn up. So, I have to get out here, they are waiting for their down their, so I’m not able to do the things on time. So that is one of the challenges that I have, yes.

Researcher: So, what is your understanding about practicing presence, what does the word presence mean to you when it comes to your patients in the unit and your staff.

Participant 07: What I understand when you said practice presence, according to me, I think I must be present on the patient at all times. So it’s what I understand when you said practice present, yes.

Researcher: Okay, so you mean being with the patient all the time doing what?

Participant 07: So, like in the morning when I arrive at the patient or when my staff arrive with the patient, I will do …….. myself because at times I have to go to the patient the whole day. I do the assessments of the patients before I start, you know, then after my assessment I will do my observations, ill check my patients safety, like around the patient. There is a checklist that we have to fill so when we at the patient bedside I have to give them medication, control and document. I have to, to do if maybe I’m in charge of the day I have to do some allocation of my subordinates and also is doing the bed bath in the afternoon and then that’s basically what I’m doing.

Researcher: okay thank you very much. So as a unit manager you have a leadership role, isn’t it, okay. So for you to practice presence, meaning like being there for your patient in totality, spiritually, physically, mentally and going that extra mile for them and for your staff the support for being there for your staff. How does your leadership role affect the possibility for you to be present?

Participant 07: Come again mam with the question.

Researcher: So, I say, being present meaning you have to be there for your patient in totality, nursing them holistically and for your staff to support your staff. Now how does your leadership role having to do all your other activities, your administrative duties whatever, how does that impact you needing to be there for your patients and being there for your staff.

Participant 07: On my side, I can say like, I will check for the day or for the next day. I will check for the personnel are enough for the patient, so if they are not enough, I will have to organize the staff so that I can be able to be in the office to do office work. And also, if maybe there’s a shortage I help them and reassure them, you know at times there will be a people who will be working after four o clock, so that I can continue with my work doing my work in the office.

Researcher: Okay, so is it a challenge at times to be a leader and to be present in the unit.

Participant 07: Yes, yes, very, very challenging. Like I indicated before like, you know there’s a lot of work in the office of which I have to be on time, do the like example, I have to do auditing, do like overtime, there must be on time at my supervisor. So, at times I’m able to do them because I’m next to the patient, that’s one of the challenges that I’ve got. It’s the shortage of staff as I indicated, it’s also the problem, really, really, yes.

Researcher: Okay. Tell me as a unit manager, how will you practice that relational care and that human connectedness with your staff, with your subordinates?

Participant 07: How do I?

Researcher: How do you practice relational care, you know that building a relationship, a professional relationship and that human connectedness, between you and your staff, how do you go about practicing that?

Participant 07: Usually we do have unit meetings, we do have some topics, like to discuss, for example we can choose the topic about the group dynamics, like how to behave in a group, how to behave in a difficult person, how to behave with the relatives so, that is happening in our unit meetings. And other thing is when we are in the unit whereby I will maybe call them, if maybe something come up, I will call them, maybe reassure them, if maybe they be having a shortage, I will reassure them that maybe after four o clock when I’m done with my office things I will come and help them, so that they can continue working, you know, it’s how i relate with my staff. Yes.

Researcher: So when your staff are faced with challenges, how do you support them?

Participant 07: Most of the time I do get the challenge from them. At times the best is to be calm at times and listen to them, reassure them through the hospital policies or SOP’s, like how things is you know, how things is suppose to be done. At times they will come not knowing that they have to do things like this, that’s why I’m referring to the hospital policies, I have to tell them no that thing you have to do it this way then they’ll say no I didn’t know because they don’t read the policies you know at times they forget all those things, so it’s how I, you know.

Researcher: And when you are having challenges or complaints from you patients about your staff, how do you go about handling those?

Participant 07: I will listen to the patient or the patient relative what is the problem, then I will go to the my staff and ask what was really happening just to investigate the matter. And then if we maybe the thing need to be solved, because at times it will be misunderstanding between the patient and the nurse, you know, miscommunication then we resolve it in my office like asking apologies and all those stuff. But if it is a thing that is really really serious matter, maybe patient fall out of the bed, it’s a very really serious matter, we’ll escalate that problem to my supervisor as a adverse event, yes.

Researcher: Tell me, if you could name a few, what might be challenging for your staff, in the unit.

Participant 07: It’s shortage of staff, shortage of resources, cos sometimes they are the one next to the patient. If maybe there is no resources they become frustrated. So they have to leave the patient in the ICU to go and ask around you know, so those things like frustrate them and then their interpersonal relationship also like people they are not the same so those things. I think it’s the things that makes them frustrated.

Researcher: Tell me if your staff, is practicing presence, they are there for your patients, they are building a good relationship, they take care of the patients in totality or holistically, what could be the benefit or the advantages of them doing that? What is good about it if they do it?

Participant 07: It’s all about like patient being like healed going out of the hospital being like healed you know going back to his family his or her family so that makes us very good as a team yes.

Researcher: Then what does it mean to the family of the patients?

Participant 07: The family.

Researcher: What does it mean if the staff treated the patient in totality, what does it mean them?

Participant 07: They become exited at times they will send the messages like through the phone, sms’s or write us some letters for us to say thank you, as a staff in general, yes

Researcher: Okay, and what would happen now say for instance if your staff now totally don’t give that special care for the patients in totality, what is the disadvantages?

Participant 07: The patient care would deteriorate definitely so we’ll having the mortality rate going high, you know. So, those are the things that will happen if maybe they are not in there ntho.

Researcher: Anything more that you would like to add maybe, that you can think about.

Participant 07: Even our image as a hospital, you know will be at the bad side you know to the community, they won’t even trust our hospital, yes.

Researcher: Thank you very much. So when you are faced with all these challenges like lack or resources, staff shortages, your duties, you know all the paper work that you have to do, when you are faced with all those challenges, how do you go about navigating around them and how do you get support.

Participant 07: It’s all about time management, you know I’ll make sure your like I will do the things that are very important. Do it maybe in the morning when I arrive, usually I arrive early I’ll make sure that I do some of the important very critical things so that I will be able to help them you know all those things.

Researcher: Okay, thank you and do you get the necessary support that you need in your position.

Participant 07: I do have a support from my assistant manager, very very because she will do the rounds to check everything is in order every week or every day it will depend on her schedule but she give me very support, yes.

Researcher: That’s good, so tell me is there anything that you would like to add, anything that maybe I didn’t think about to ask you pertaining to you practicing presence being there in the ward for your patients and how do you practice presence when it comes to your patients in the ward. Anything else that you would like to add.

Participant 07: Just rephrase the questions.

Researcher: Is there anything else that you would like to ask about how you practice presence being in there for your patients as well as for your staff. Anything that you would like to add.

Participant 07: No nothing perse but at times especially when coming to the shortages of staff neh it would be wise if maybe our, because we’ve been scheduled to work only 24 hrs of which is not enough. We still have a shortage even if we work that 24 hrs off which at times we are having a shortage, if maybe the management can increase the working hours, argg the what you call it?

Researcher: Overtime

Participant 07: If the can increase it then it will help for shortage of staff.

Researcher: But if they increase our overtime, what to you think could be then what is the reasons why the don’t want to increase the overtime.

Participant 07: No. It’s all about saving the money, budget. So they have to save the money to work on the budget of which we are not working with the budget they are the one who are working on the budget. At times like for example, last night people we were only 4 with 6 patients, critically ill patients. Two, one nurse had to work argg, two nurses had to pair the patients of which and they were very very critically ill, off which it’s straining for them.

Researcher: But when it comes to the staff’s perspective do you think that giving them a lot of overtime could maybe not lead to negative effect on the staff themselves.

Participant 07: It will yes, but it will help for the patient because the personnel will be more for the patient, yes it will.

Researcher: Anything that you will like to add.

Participant 07: No not at all nothing. But what I can say I’m happy if people are coming to do some research, like you do, so it shows to us there are people that care for us. Yes.

Researcher: Thank you very much, I really appreciate you taking this time off to answer a few questions, should I have any follow up questions, it’s okay if I come back.

Participant 07: You are very very welcome.

Researcher. Thank you very much. Keep on doing the great job that you are doing. Okay and God bless. Okay, bye.

Participant 07: Thank you so much

Male; 56 yrs; 1 yr unit manager

**Participant 08:**

Researcher: Good day ma’am. My name is Bernardine Smith and I am a master’s student at the North West Universitas. Thank you very much for affording me this opportunity during your busy schedule, I know you guys are very, very busy and I really appreciate it. Remember there’s no wrong or right answer all I’m interested in is your perspective. You can stop me at anytime or you can add any information if you feel that I’m not asking the sufficient questions, okay.

Participant 08: Alright

Researcher: You don’t have any problem with me recording.

Participant 08: No.

Researcher: Okay, thank you. If I forget to ask you a few questions is it possible if I can come back on a later date just to ask one or two follow up questions, if needed.

Participant 08: No you can.

Researcher: Okay. Thank you very much. So my first question is a very broad question. So in general if you have to look at the topic being, Presence practices amongst the unit managers, what are the presence practices amongst the unit managers.

Participant 08: Present.

Researcher: Presence

Participant 08: So how does the staff.

Researcher: The unit manager practice her presence in the unit.

Participant 08: Okay, like when you are a manager mos you must communicate with your staff. So like every morning when during report taking I’m there with them so that I can know what is going on in the unit and then to understand if there is any challenges like staff shortages or equipment. And then also like if they having problems my office is always open like social, psychological problems. Also confidentiality is very important if you are a manager or my staff here they are very free to com to my office because we are talking about like everything, so I’m there for them always.

Researcher: And for your patients in the unit, how does unit managers practice presence when it comes to their patients in the unit?

Participant 08: Like here in my unit every morning, I do greet them and then interview them like daily on daily basis about any challenges any problems, yes.

Researcher: Okay, and when you interview them can you name few of the challenges that they sometimes have.

Participant 08: The challenges sometimes the nursing staff others especially at night mos they would be rude maybe they are tired so like when the patient is asking for something they would be like rude to the patient or ignorant. That is the challenges that hmmmm.

Researcher: Okay, so how do you navigate around that, how do you guide your subordinates when you get these type of complaints.

Participant 08: I usually call the person into my office and then I will tell him or her that the patient complained about one two three, what was the problem. And then she or he will say whatever and then I will say lets go to the patient you must go and apologize cos what you did was really wrong even if like the patient mos is always like even if you sometimes there’s rude patients but as a nurse you must not go up there you must be always be calm even if the patient is rude to you, you can always call somebody, there’s always a matron on call even at night.

Researcher: Okay, so if we have to look at that one word presence what is your perspective of it. How will you define or describe presence?

Participant 08: Presence like they must feel, they must like well your presence, they must respect you, is respect. And then there must not be afraid even if you are there like if your staff they must feel free to say, to work, sometimes when the matron is there others will feel intimidated but they must just feel no this is our supervisor like they must just feel free.

Researcher: And how would you describe presence, what is presence when it comes to nursing care?

Participant 08: Joh, I can say to be always be there for your staff for your patient., hmm, hmmm.

Researcher: Okay , thank you. So as an operational manager you have a lot of responsibilities, besides the fact of being there for the patients physically, mentally, psychologically. How does your leadership role affect or impact on you being present inside in the ward?

Participant 08: I don’t understand like

Researcher: So you’ve got other operational duties that you need to do, leadership duties. It’s not only being there in the ward for the patients and for the staff, you’ve got a lot of other things that you got to do. So all that other things that you have to do as a leader how does have an impact on you being able to be present to support your staff and to provide the care to your patients.

Participant 08: It does affect because, most of the time I’m not always there in the unit. Like I’m a manager I don’t have a PA neh. I’m doing stock taking im doing orders, I have to attend like meetings and then I’m doing inventory, so most of the time I’m not in this office. Like, so I’m sure only during report taking I’m there with like the nursing staff from there’s a lot, I must audit do a lot of things, so it does affect my presence in the unit.

Researcher: Okay, thank you. Seeing as you are a leader here of this unit, how do you as an operational manager practice that relational care, you know, relational building that human connectedness with your subordinates? How do you practice that?

Participant 08: It’s sometimes so difficult neh as a manager mos when you become too friendly to your staff, they will take like advantage, like they will maybe think no she is our friend like again when you like distance yourself like I’m sitting in my office I don’t again it’s a problem. So it’s very difficult sometimes, yahh because I had a challenge, there was a nurse here like I was very close like with her like but she was going through a lot so I tried to bring her closer to me like but at the end huh well she just change like ahh, she lost respect like for me, you understand that’s why I said its so difficult so to bring them closer and again to be that distance from them so it’s a bit challenging, but we must try.

Researcher: Okay, so how do you provide support for your subordinates, when they need it?

Participant 08: Like I speak to them. So if I cannot solve like a problem, we having EAP program in the hospital. I will refer them to the EAP like for help.

Researcher: Okay

Participant 08: And then do follow ups and sometimes maybe sometimes if the problem you know when you are working in an environment and then I’m working with you maybe I’ve got issues with you, like I’ll try to find out what is the problem, then I will separate them like in a shift maybe the other one will go to the other shift and the other one to the so to prevent the mis understanding.

Researcher: You just mentioned misunderstanding, what causes sometimes the misunderstanding amongst staff, or yaahh, the misunderstanding?

Participant 08: Sometimes it’s work like related. You’ll find out there are those I’m saying lazy so laziness, so she always expect you to do like checking the emergency trolley and do what else like and she’s sitting or just focusing on her patient, so when you confront her then it’s becoming like that misunderstanding. Sometimes it’s social things from outside the work environment, so and the gossiping it’s a problem.

Researcher: Okay. So when your staff is practicing presence meaning that your staff is there for your patients, they care for them holistically, what would be the advantages of that?

Participant 08: The advantages of when the staff they like taking care of the patients

Researcher: 100% what will the advantages be

Participant 08: The advantages is obvious gonna be less stay in ICU. And then less incidents and then there are questionnaires that the ntho our patients when they are being discharged, so positive feedback like from the family or the patients themselves.

Researcher: Okay, and what do you think should they not be taking proper care of the patients in the hospital, what will be the disadvantages of this.

Participant 08: Well there will be like lot of litigations. If like there is no proper care, obvious it’s gonna be like long stay of patient in like in ICU and infections, what again or even the name of the hospital like people will gonna talk bad things about the hospital like there’s no care there, you see.

Researcher: So what can be the advantages now if you as a unit manager, if you provide proper care and that support and assistance to if you are present for your staff and for your patient? What positive can come out of that? What is the advantages of that?

Participant 08: if your personnel they are satisfied, happy and you always support them, even if you are having like, we having shortage of staff like in South Africa, even if there’s a shortage, they will always be willing to help, you see because they know that our matron is always there for us, so they will support you.

Researcher: And what could be the then now the disadvantage should you not be present there for them.

Participant 08: Joh, you’ll be left alone here in this unit. Like today in neuro ICU the matron was alone. She had to call people from other units to come and help. The staff all of them they are sick you see. So they will sabotage you if they are not happy. And you will always go to answer about the mis management of patients, all the incidents you will have you’ll be responsible.

Researcher: And if you are not there as a unit manager for the patients what’s the disadvantages of that

Participant 08: Like if I’m not there for the patients

Researcher: Yahh, if you don’t practice presents for your patients

Participant 08: like if I’m not there maybe the patient will not be able to verbalize whatever they’re not satisfied about so we will not know whether the staff were good or bad to them so we will not have the proper like feedback you see if we are good or bad.

Researcher: Okay, thank you, facing with you facing all these challenges, staff shortages, patients complaints and stuff like that having the workload a lot of work load, are you getting proper support yourself.

Participant 08: No ways, I don’t get support from anyone. If something is wrong I have to even let me say I was busy with something else and then I have to submit, if I’m late then I have to answer, they don’t like check whether even if I can explain you know what I had a challenge , there were no staff so I had to do one two, three if they want this they want this, you see. I had to answer no excuse. There’s no support at all, at all.

Researcher: Okay, is there anything that you feel I should have asked or just anything that you would like to add from your side before we end everything, the questions.

Participant 08: Like, I feel like, the subordinates neh, I don’t know as a manager we are also human so they expect us to even if you can come to me and say being rude I also have emotions, so I can even myself I can also be high there neh, I can also be rude sometimes, its emotions, so they think we must always be calm, sometimes it’s not possible, some situation they, you will be out of your way and.

Researcher: And when they complain to you about that how do you handle it?

Participant 08: No, I apologize I’m saying you know what I’m sorry I was rude. I’m also human but also please when you come to my office, you must come with respect you see, so they think we are not humans.

Researcher: Okay, thank you very much, I really appreciate, I know you guys are busy, there’s shortage of staff and there’s a strike here as well. Yes, thank you very much for availing yourself. If I have any follow up questions like you said it’s fine if I come, is it okay. Thank you very much, I really appreciate it, thank you.

Participant 08: It’s fine, thank you.

Female; Age-50 yrs; 1 yr unit manager

**Participant 09**

Researcher: Good day matron, thank you very much for affording me the opportunity. My name is Bernardine Smith and I’m a masters student at the NWU, my final year. Thank you very much, you don’t have a problem if I record the, is it fine. If maybe, it’s my first time doing research, if I have follow-up questions, would it be fine if I could come maybe just back for one or two follow up questions.

Participant 09: okay no its fine

Researcher: Thank you very much. Like I mentioned there is no right or wrong answer, its just your perspective and I promise I would take up a lot of your time but I hope our conversation will be so nice that we actually interact little bit longer with each other, okay. So, like I explained my, research is about presence practices amongst unit managers neh, so I want to know according to your perspective what do you think are the presence practices amongst unit managers, in general.

Participant 09: How, how, I don’t understand.

Researcher: Okay, remember I explained what presence is, presence means in the sense that how are you there for the staff and for your patients, not only physically but emotionally and psychologically and that’s now my definition of what presence is, I don’t know what how do you classify or understand.

Participant 09: What I understand is that as a manager you do everything because even I, we go in the ward just today there was only one sister so I have to help in the morning until now. So just all over.

Researcher: So what is your understanding about presence. How will you define presence in general when it comes to nursing presence.

Participant 09: Presence is just to be there everyday for the them and for the patient. Ja, because previously we have to sit in the office and do office work but now, it’s not like that, we have to work also in the ward help just like I tell you from in the morning I never worked in the office I just worked in the ward, because of shortage.

Researcher: So you said you have to be there, so you have to be there, what does that there include.

Participant 09: To be there include nursing in general, everything.

Researcher: Okay, so just doing nursing practice.

Participant 09: Yeah just doing nursing practice in general, ja that’s it.

Researcher: So tell me, mmm how do you as a unit manager practice like relational care, relationship building and human connectedness with your subordinates.

Participant 09: So I don’t understand. Because we use to be there for them every day and then we worked as a team.

Researcher: so how do you build that team, how do you build your team, to build that relationship, a good relationship between your team members.

Participant 09: We do the activities, different activities. Sometimes we go out, sometimes we make parties in the ward. Some events with all the teams, with different akiri, we have different shifts night duty and day duty, so its how we make it. Always, we work as a team even with the patient ja.

Researcher: And how do you build relationship with your patients in the ward.

Participant 09: Relationship with the patient?

Researcher: With your patients in the ward, professional relationship, I don’t mean a personal relationship. How do you build a professional relationship between you and your patients in the unit.

Participant 09: Okay, in the units, always in the morning we talk to the patients. I book time to sit with them and talk to each one individually. To hear their concerns, how they get their treatment from the nurses and then solve the problem if the problem is there immediately and I asked her if they have a problem not to shut up not to be quiet. They have to step up if there is a problem and come to me and explain what is happening. That’s why we have to build a relationship cos every time when I enter in the morning they know I’m the matron and then they know if they have problem they ask where is the matron and they will call me and then I will go to them and hear what the problem their concern.

Researcher: So if you can name a few, most of the times, what will the problems be like.

Participant 09: The problems is like eee maybe they not satisfied with the food, not satisfied with other nurses attitude, other not all of them, ja. The bad attitudes we have to correct it immediately, ja.

Researcher: So how do you address the attitude problems, if there’s attitude problems from nurses.

Participant 09: Attitude, I just, in general I just address it in general. If it’s sometimes one person or other person they do it continuously, I have to talk individually to that person, to correct the mistake. If it’s continuous I have to discipline.

Researcher: So how do you as a manager, practice human connectedness when you connect with your staff, when it comes to support and guidance.

Participant 09: Yes when they have support they know mos they have to come to me and then I make time for them to talk. If they have problem they sit here, we talk and then we find the solution, how we are going to deal with that solution and I give guidance where I have to, ja.

Researcher: Okay, thank you.

Participant 09: We talk to each other no problem. In which case I told them communication is a good thing. We have to communicate not to keep quiet, ja. If you have the problem just talk and then you to deal with it, to deal with it.

Researcher.: Thank you. As a unit manager I know you guys have a lot of work to do like you mentioned earlier on when the staff is not here you have to stand in and you have a lot of other duties as well that you have to do in the office, yes. So tell me now how does that leadership role impact you, or impact your ability to practice that presence in the ward, being there always for your patients.

Participant 09: Yes, they have that negative impact, not because I have to work overtime. Like yesterday I knocked off at pass five because I never finished the office work. I have to sit after I finish in the ward to come here and sit and finish my work, ja. Because at the end you have to do your work.

Researcher: Yes, how do you get your support when your workload becomes too much.

Participant 09: I have the assistant manager who is very, very, supportive because every time if something I didn’t do it if the deadline is past I just explain to her that I didn’t finish my work because of one, two and three and she understand, and she supported me that I can do it when you have time.

Researcher: Thank you. Two more questions, okay. So, hmmm, practicing presence, I will take it as something happens in the ward, okay. So what is the advantages would you say? What can come out positively, what is when the staff as well as you practice presence, not only in your ward but with your staff.

Participant 09: With my staff.

Researcher: What positive can come out of it.

Participant 09. The positive thing is that I gain an experience. If you are the manager, you do all the work. Its not the manager for the office work and then to them I like to work with them. I don’t like to sit in the office for the whole day because the positive way I have to see if they do work and I’m so happy because when I go there I find that the work is going on. They didn’t say we are short of staff, we didn’t do this always we have to be positive. Even now its only one sister who worked from the morning, I helped.

Researcher: Okay, and uhh for the nurses, what positive can out of it if the nurses practice presence there in the ward with the patients.

Participant 09: The positive thing is that for them I don’t know.

Researcher: For the patients.

Participant 09: For the patient. No always the patient they will come after the discharge they have the discharge questionnaires that we give them just to tell us how we treat them and everything and the questionnaires but every time it’s the positive reports. I didn’t get any lot of complaints. Ja, because always the complaints we will just deal with it immediately.

Researcher: Okay, so how would the, would you say would the patients feel if the nurses not always there with them and they not doing there job. How would the patients feel about that.

Participant 09: Yoh, they feel great, I don’t wanna lie. They feel great because every time I ask them how do you feel, how do you treated, they say we love this sisters we don’t want anything but always they are there for us, when we need them, they help us all over.

Resercher: Okay

Participant 09: I didn’t get the negative attitude, sometimes for that time is one nurse. Sometimes we come from home with our own things, we have the stress from home and when I ask I find that this person is having a problem really that day.

Researcher: Okay, and tell me now if the nurses would not be able to be there what would be the disadvantage be of it.

Participant 09: The treatment, they are not going to get the treatment that they suppose to. If the nurses are not there, they are not going to get attention and the treatment, they are not going to get the treatment, the problem is that. That’s why if there is a shortage of staff I have to stand up make sure they get treatment as they should get it. Because, now we are here for the patient whatever our differences, our problems, patient first.

Researcher: Okay. Thank you. Tell me how do your staff feel, all your staff feel when you practice presence, when you are there for them.

Participant 09: They feel great, they feel great if you are there, matron you are here yes, they feel great.

Researcher: What positive can come out of that?

Participant 09: They know when I’m there they work, they can work as a team and they ask questions where they don’t know. I’m there to teach them. To supervise them, if they don’t know anything they know I’m there and they will come matron don’t understand this come and help and I go and help them.

Researcher: Tell me, I think we’ve come to an end.

Participant 09: I can tell you here in ward 3 A we work as a team, we work as a team really, so I didn’t find anything that I can say this shift I have the problem with this shift, no. they work as a team because I told them if they have the problem with someone come to me and then we can talk. Eyy, so we can solve the problem. Because we are the people, we are human beings.

Researcher: So matron is there anything else that you maybe think I should’ve asked? Anything that you from your side would like to add about your position what you are doing here concerning practicing presence, in the unit, is there anything else that you think that you would like to add.

Participant 09: I think what I can add is that its hectic here, due to shortage off staff. Sometimes we are so exhausted even the nurses I see sometimes really they are so exhausted but we keep on working, we keep on working because even if there is no someone, I have to call them come to do overtime, come to help us. Sometimes I feel this person is exhausted but there’s nothing you can do, we have to look after our patient.

Researcher: Okay, so what other support are you able to give them.

Participant 09: The support that I give them is I’ll be there for them. They know even if I’m off during the week there is a problem, they called me and I come and help them. For there’s nothing I can do. There’s no way I can get other people somewhere else. So I’ll be there even if I’m home, they know they call me, matron come and help, I come and help.

Researcher: Okay

Participant 09: Even, someone if he sick, okay matron I’m sick, okay I’m coming, go to the doctor.

Researcher: Okay, thank you, that’s good.

Participant 09: and they don’t stay away from work, I don’t even this one today that is not here, I know very well, he’s not someone that stay away from work, if she called me I’m she’s sick I know she is sick really. Ja, they don’t stay away from work.

Researcher: Dedicated. Okay, thank you very much matron. I really appreciate your time and like I said if there is any follow-up question, you said it’s okay if I come back. Thank you very much.

Female; Age-50 yrs; 6 yrs unit manager

**Participant 10:**

Researcher :good morning matron, my name is Bernardine Smith and I’m a masters student at the North west University, hopefully my final year. Firstly I would like to thank you very much , I really appreciate you offering up your time knowing that you are busy and short staffed to answer a few questions. Like we spoke to each others , there is really no right or wrong answer neh, I’m just interested in your perspective, okay. You don’t mind if I record this session.

Participant 10: I don’t mind you can record it.

Researcher:My first time doing research so, I might not ask all the correct follow-up questions, is it fine with you should I need additional questions, if I can come back.

Participant 10: Yes

Researcher: Okay, thank you very much

Participant 10: You welcome.

Researcher: Thanks matron. So, my first question is; according to your knowledge what is the presence practices amongst unit managers in the free state

Participant 10: I call it like that now to my experience having 3 years now as a unit manager, a challenge that we had in our department mostly that I’ve noticed that its there is shortage of staff. If it can get enough staff, than that now our work will be easy. And our patient will have that total nursing care they supposed to get especially when we work in a specialized unit. And my suggestion now was that now maybe department of health could consider it, when you working in the specialized area, the susters working on the patients must be one on one with the patient rather than to have every 2 personnel for four patient especially critically ill patient, that some other things especially documentation like care plan and continuous evaluation of the patient that will be in a high standard when somebody is nursing you one to one patient its going to be in a higher, if you are looking at one patient rather to look so many patients at some stage, you rather some other things that you supposed to look at the patient ignored because of focusing on so many patients. So if you can have enough staff, I think it would be the best ever and that one.

Researcher: Thank you matron, so tell me when we talked about presence practicing, or practicing presence, how would you define practicing presence? When you think about it what is presence practicing?

Participant 10: The practicing is like I said before, I use to be a professional nurse in the department before we had enough staff and now right now I have to go and look at the office and I have to go and look inside, check that everything goes well, make sure that everything is in place at that time the only nurses the in charge was in overall for all the patients, as the matron you do you part. But now its double shift because we doing everything. And if you can get enough of the equipment, shortage of equipment also restrict our hands to give our best nursing care to our patient because we sometimes don’t have equipment to do our work with especially like linen shaver, an example we have a patient who is suffering from diarrhea that is a patient who is prolonged on antibiotic that need a linen shaver to be covered our patient’s never had bedsores though even if you having an NIMBUS-mattrass to protect the skin of the patient, patient must have a napkin , linen shaver, and must be nurse on the nimbus -mattrass all of them, then I think the risks of the skin of the patient in that way can be protected. If you having a four bedded patient let all the beds must have a nimbus mattrass of them so that our patient they are lying here helplessly on the bed not being turned time and again we turn only 2 times in the morning and in the evening when we wash them. Then those, nimbus will relieve those pressures to the patients in some other patients they have sensitive skin. So, we don’t end up having an adverse event where our patients end up having bedsores. And, now even for that matter the patient that are laying, lie here in our department, to reduce long hospitalization, nursing the patient in totality will reduce the patient to be here to be nurse in the ICU and go to the ward, they mobilize in the ward but now because of there’s some equipment’s we nurse the patient with the big wounds here that take long to be healed that need special care, that take time, that take long in the hospital and the patient end up not being well when she arrive there especially I’m working in the neuro ICU, you find that now patient does not have a family which is very painful for me as a supervisor or matron of the department I have to call the police because of the patient that is coming from Dihlabeng, Lesotho we don’t have those patient, and when the patient arrived here maybe for example when I make an example the patient is having a multiple aneurism doesn’t have any family from here, to get the consent we have to go through social worker, we have to go through the superintendent or the CEO of the hospital to sign the consent of the patient so that the patient can be operated. Because everything it needs to be done according to the patient consent giving the consent for the operation, so some other patient really, they are suffering because of they are coming from far and as the hospital is accommodating Free State as a whole, we can’t say we cant take the patient we are here to help the patient so that the patient no matter the family are there or what but the patient must be helped, you understand to save life of the patient. Like sometimes now I’m having the patient that’s the child that is 21 years old that is deceased now that I have to look for the family, to make them aware, no contact numbers, the patient’s been here and been deceased and it’s very emotional and difficult time when the family members arrive at the hospital and you told them that your child is already passed on without not knowing at that time that the child was critical ill, yeah, because the condition of the patient change time to time especially in the department where I’m working, it can change time to time the patient been send alone with the transport to the hospital. I think also that must be a family member who accompany patient so that can get the contact numbers, so that we phone those family members for any changes happens to that patient, so that they don’t hear at the last moment when the patient doesn’t do we’ll , and then the doctors also don’t have to struggle when they have to done an operation on time. Some other patients, they do delay on the operation because of the contact numbers of the family the consent and everything, that they could be helped.

Researcher: Okay, that’s sad neh. Yes, you mentioned something that because of the shortage of staff the patients, if there were more staff, patients would be able to get total care, okay. So do you want to elaborate a little bit on that for me.

Participant 10: Yes. When I’m saying shortage of staff, when we are nursing two ventilated patients. It becomes difficult to do everything at your level best that cause the staff also to be exhausted, but if you are focusing on one patient, you do everything. You’ll make sure that everything goes well, where you supposed to even then to turn the patient as per you usually do when we been taught nursing taking care of the skin of the patient cause there’s no time. We are rushing that now this two patients just have to write the report about them and give them medication that is all and lets make sure that the patient is well, but focusing on that one, only one patient with the shortage of staff that’s really disadvantage most of the personnel do their level best because of nursing, you’ll find yourself sometimes nursing three ventilated patients, how will you going to be, you come exhausted. When you go to the third patient some other things you don’t do what you supposed to do because you are rushing to finish the shift off. And the long shift that the nursing are working according to me is too long, it’s really too long but we can’t do anything about because now we have to work 40 hours per week, it’s two days, two days. Some others are working seven nights in, for the whole seven nights so the staff will come in emotionally exhausted and physically exhausted and mentally exhausted. How do you emotionally and physically and mentally exhausted to help a critically ill patient? You know very well now that you are two with four patients. Some other things that you are supposed to do like you suppose to do just do to finish the work, but not giving that thing that love the patient needs. Our patient here needs love more that anything. They love, want caring, they want to be cared for because they are here. They are here so that even for that matter, even those they are having the family, their family bring the patient to the hospital, thinking that our family member will be well cared for even if she’s from another province and she’ll be well cared for. But now because shortage of the nurses really, I think that now if you can get enough staff from those nurses nursing don’t have a struggle if we getting the post, taking long time for the post to be advertised. No matter even if it’s the lower categories. It’s the student nurse most of the colleges are closed for training of nurses, training of nurses, recruitment that is the best way to cover this shortage . if you can get enough, recruit more nurses, than it will cover everything that you are here because now there will be enough hands and replacement of different nurses of different categories in different departments in the ward and what so ever. For that matter I’m not working in the ward, you find one professional nurse with 34 patients giving the medication. Really do you think how strong you are mentally and physically that is exhausting. Nurses really, when you work until you don’t want anything, nursing 34 patients, too much.

Researcher: So is it safe to say that the nurses as they would like to care for their patients in totality, they want to be present there psychologically, physically, mentally for the patients, but due to the shortage of staff and the amount of patients in the hospital they are unable to actually give their level best.

Participant 10: Exactly, that is my point, yes. I think really that is the most thing really for that if people really they will complain of if you can get matron the staff and we will do this. Like a nursing processes, a nursing processes that is where you will see for the first time when the patient come to the hospital. You identify the problems and you follow up the problems, but now the shortage. You see the patient is having problems and you will just record it but you don’t follow up. Is that patient progressing from that problem that she been coming for because of that hands, do you understand. When are you going to follow it up because mentally you forget that, physically exhausted, mentally exhausted but if we have enough personnel our nursing processes will grow much better and our caring of our patient will much better, in our publics really, in our community because really in nursing is a calling but it is a calling, yes we understand but with hands, short of hands to nurse this critical ill patient. My point really is the shortage that we are facing, to give our best care for the patient. According to Batho-pele principles in the department it say the patient deserve best quality care but how do you give a person care quality care when there is skeleton staff, you can’t. how do you do your best to the patient when you exhausted physically and mentally and the most important thing is about the training, training that is the one. Go to the conferences that we use to go, moreover refresh your mind, some of others they’ve been long time in this department as professional nurse, to refresh your mind you learn so many things, you learn so many ways how to nurse your patient, you learn so many things to come with a skill where you have learned but nurses are not been send for training, nurses are not been send to the conferences. I can’t say for 20 years I would still be like before, I’ll be lying, my mind needs to be refreshed with some new fresh things and new ideas so that now you give best care of your patient.

Researcher: Thank you very much, matron. So tell me, you as a unit manager, how do you practice presence inside the unit, to your patient?

Participant 10: My patient, like I say right now, if I operate with 2 personnel, the other one is sick, I have to go inside and close the office. I think my patient comes first. I close like you’ve seen you get me inside, I think the best thing that is the patient. I rather leave my paperwork and focus on the patient so that my patient can get the medication that they are supposed to get, my patient been suctioned, my patient being washed and patient were given whatever that I can. I rather leave this papers in the office and attend to the patient. What I usually do, I’m suppose to work until 4 o clock but because there will be only one sister with this critically ill patient, I go and help by making sure that I go off even if I can work until seven o clock, I don’t mind. But as long as I have know that I’ve done something for this patient that are lying here. That we are here for them, we are not doing any favors for nursing this patient. Is not their choice to be ill, but their sickness make them to end up in the hospital, to be lying helplessly like this. That is why I think like let me close the office and go and help that patient that is helpless on the bed, that can’t do anything for themselves.

Researcher: Thank you matron. Now tell me how do you then practice presence for your staff in the ward?

Participant 10: They know that when I’m there I’m here , I go all out. What I do , I just make sure that the equipments in the first place they are working, make sure that those that needs to be repaired they are being repaired. When there is a need I’m here for them, sometimes I go all around to make sure they don’t short everything, make things easier for them, to make work easier for them then I’m here. You understand, sometimes you can see when I’m here at work it will become so relaxed that the matron is here because they know even if we don’t have any equipment or they sure I’ll go to other departments or I will go and if the patient is suppose to go for a to the brain scan I’m here I don’t mind to go to send the patient to the scan you understand and you continue with another patient and connect the patient and to make the patient and even for that matter I can even make sure that the patients been washed or is clean making turning of the patient, though I’m the matron, you understand not forgetting I’m the matron but my priority is those people that are lying helplessly on the bed. It’s the patient that I’m here for. The other thing if I can see the unit is quiet, it’s then I can sit and do some other duties that I’m supposed to do. And we work as a matron we have some other challenges is whereby some other things they need you alone, and it’s difficult even for that matter to give some of the personnel also maybe to take one file to audit some other file because they overloaded with some work inside. So you better do every thing for yourself, end of the month everything has been covered, that is supposed to be done. Everything according to your job description as your matron.

Researcher: Thank you matron. You just mentioned job description, so how does your role as a manager with all your job description, how does that impact you from being present inside of the unit?

Participant 10: Our job description as the operational manager when I get the post, it was said that now as a unit manager, you check every body. It’s overwhelming because now you become responsible for those people who are not coming on work, you come responsible for the people not doing their work correctly, you come responsible that the work must be done as it’s supposed to be done, you understand because at the ultimate end the responsibility and the question to be come to you. When the doctors need a bed, you can’t refuse any patient as a matron. They come to you, if the patient is there, it’s an emergency, you just have to make a means that now, you make sure that now, even if you can see really these people are short but you can’t refuse the patient. The patient must be helped, the doctors will say I’m having the patient in theatre, they can’t breathe and everything, he need an ICU bed, the only thing what to do, even the same shortage, you put the shift in so that the patient can be helped inside, that’s the only thing. Our job description people they don’t work according to the job description, if they have to stick on the job description, it doesn’t work at all. If I have to stick on my job description I couldn’t have been inside because my job description say supervision, organizing and planning, but I’m nursing. Making sure that everything goes well because if anything goes wrong on the same patient that I, it will come back to me and I will be the one who needs to write an adverse events, so by going inside I’m avoiding writing incident reports, you understand, I just want everything, the patient to be fine.

Researcher: Thank you very much. So with this shortage of staff and you also mentioned that your staff is emotionally drained, physically drained exhausted, how do you provide that relational care to them?

Participant 10: Usually, there’s something that we call that is the PDMS. Even in the PDMS, according to the hospital policy, even if you see that the person is doing well you can’t give more than expected because of the financial restrains you understand. You just have to go and just say thank you, that’s the only thing. And now thank you to other people are not enough, people they want money. If people can be paid for PDMS’s with outstanding amount and giving even though we are shortage giving enough it’s been cut from one day of overtime. You understand, we use to work 36hours now we have been cut to 24hours. People they even want to come and help because there is no money for overtime, they don’t come. Because now they have to wait you as a matron until there’s no patient then you give their off time because they have come for extra day to work in the department. And if you say to the people okay the 36hours per month for you for overtime, I think it’s then that they will see people coming to volunteer to work overtime and I think the workload even if they are having the shortage will come and say but now overtime is only one day. If you have work for the whole month you have worked, if you having 12 personnel, they are working only 12 days how many days are left with that which means on other days those that are left behind they have to work like that. Because now all of them the whole 12 people have already worked. And if you say come and work I’ll give you time of when are you going to give the time off whereas the patient they are always here. We can’t say the patient can’t be admitted. I think going back again of giving people overtime, recruiting, training really will relieve the stress and boredom and motivate some others that want to become we are still having matriculant that want to be a nurse. How we’ll talk about them, you understand. Nursing it was a profession where everyone would like to be a profession but now you find yourself o my goodness it seems that I’ve chosen the wrong path, because of long hours, exhaustion, mentally are you with me. Even time, even the people if maybe they are retired on the profession, they take long to replace those retired nurse. Now before I become an operational manager the manager I was working with if you know that maybe in two years-time so and so is going to go on retirement, she put the request that I’ll need this to person to be replaced but you know, it takes long for those people to be replaced if the people they are long been retired here but these people are not been replaced. So you are left with whatever that you have. If maybe you are 12 the two retired now you wait for a very long time for those people to be replaced. And let other people work in the department where they are willing and be effective, like there are many nurses that want to work in a specialized department let them give the opportunity to come and work wherever the want. It’s really depressing to work in the department that you don’t like. You thing that they are going to be effective, no, let work really is really the work that you motivate. You know that if I’m going to the specialized area I’m going to be trained, I’m going to be developed, I’m going to be a better person, then I can work overtime. Money is the important issue for everyone, that one I don’t want to lie.

Researcher: Okay, so as a unit manager how do you build that human connectedness with your staff, where you support and guide them, how do you build that human connectedness between you and your staff, your subordinates?

Participant 10: The only thing that I do, my staff even then I will say can we please go contribute, I will ask then can we have a lunch or tea or everything and then or in December month then I’ll ask can we please go and eat somewhere so that we can just have like a closing party only once per year, it’s how I try. But I will say okay or going for a massage only in six months when I see there is a special somewhere then those who they are working night duty off and there are those who will accommodate then I’ll try another group when they are off and go and book for them and so that they can have massage. And that’s is how just to motivate them to be there I will say okay go for a picknick here’s some drinks sitting together and then making that now we enjoy it together all of us, even if it’s just a nice breakfast, remember always each and every time I’ll be just take them to second avenue where we go and some you walk and I put the table of maybe six people and everything and then I set up time and then they know at one o clock then those when they are off even if they are working night duty I’ve booked the table then everyone sitting together order a meal that you want, order a drink that you want with them just forgetting about the place here, just going to just to relieve the stress outside in another environment, talking to some other different issues, even though we end up talking about really the situation that we are working in but now we’ll be sitting together, it means a lot.

Researcher: Thank you matron. Then when staff practice their presence in the unit, what are the advantages of that? What are the advantages when staff practice presence in the unit?

Participant 10: Advantages, is due to be more confident, confident and more skilled, you understand. It’s better if you are confident enough to know what you are doing, you understand, really. It’s not nice to work in a ward and you do not know what you are doing. Being confident that really that is my speciality I love to do this work you go in totality to do this work, you understand, that is the only thing we see of the staff when voluntary do the work as being expected as a nurse to do on their patient.

Researcher: And what will the outcome be if they practice presence?

Participant 10: The outcome, I don’t know how, usually what I do usually I will give some say okay this one have done this or if maybe for the past 6 months on my own in my department, if somebody have done and everything correctly I will buy some a cup just to say you have done well good for this, you understand. Others are depending being depressed neh because of some other social problem that I have but what I usually do I will let the staff to contribute whatever they have to buy something, it can be a slipper or whatever or a present or whatever, just to say. Even if one of us is being hospitalized I encourage the staff to contribute some other things just to give you some flowers in the hospital, they’ll go and visit you and see how are you doing. We are having a group on our whatsapp for every problem. Where we motivate our staff and talking and everything. I’m having like I don’t want to say it’s a society but a birthday when you are birthday I’m having all the names of all the personnel that on the list I know birthday and after doing the birthday you put different maybe we’ll say a kettle, maybe cutlery and everything whatever that you choose you know that now it’s what you want like raffle you choose for you and then we’ll buy you that for that year for each and every month on somebody’s birthday.

Researcher: Okay, so tell me now. If the staff do not practice presence what is the disadvantages of that?

Participant 10: the patient will die, definitely the patient will die. That is the only thing the patient will suffer, there’s nothing. If you don’t do anything the best to the patient that you are here for, who’s suffering, that helpless patient, if you don’t do anything if you don’t do wound dressing on daily basis the patient will end up being septic, if you don’t clean the wound of the patient the patient will be septic. If the patient lying for a long time on a diarrhoea the skin of the patient is pealing off, you understand, really but doing everything that’s supposed to be done to our patient I think our patient really can be, be not being long in the hospitalized in the department and being transferred to the ward very quickly.

Researcher: Thank you for that matron, so tell me now, if you as a unit manager, if you practice presence, being there for your staff and for your patients in totality what is the advantages of that?

Participant 10: Disadvantage?

Researcher: The advantages, what good will come out of that if you are there for everybody?

Participant 10: If I’m there for them, I feel good because I’ve done good for the patient not specifically for the staff for the patient, you understand. When I want to do good is for the patient, even I can help the staff but I want to do good to the patient not for the staff, you understand. The patient are my priority then the staff even though I help the staff , the patient because I’m here because of them, they come first. And what I teach each and every of my colleagues there the staff I will say can you please treat the family of the patient with respect because some other family if you treat their family of the patient with respect you see now they can get some advices from the family, they get some information on the same family talking to the family respecting the family, I think that now if you respected that family we’ll here everything even if you don’t know what is going on about the patient then it gives you better option on how to treat this patient. Let me say for example the patient is an alcoholic neh where are we going to know, if the family does not tell you or maybe he’s using some other drugs. And confidentiality of the patient in nurses is very important, let the patient trust you whatever she told you that you will never say to anything and the patient will be free to tell you whatever problems that you can know that you know that talking to this person I know that now I have relieved myself to somebody who can help me. Does not mean that now anything that patient has told you, you, just have to gossip with whatever the patient deal with you is what I say. If the patient told you something does say you have to go and just say it, keep to yourself. She tell you because she trusted you, let our patient trust us, that trust and love of our patient that can make our patient healthy and go home healthy, that is only love and trust that can make our patient to can give the patient and support with the family even if in their last moment of a dying patient, be there for that family, so that the family can never say even if there love one is going to pass on but due to your support that you are going to give them and the trust and the love and everything, you relieve them with their grieving processes. Treat everyone equally.

Researcher: Okay, thank you matron. Then I also just want to find out from you now if you as a unit manager of your unit, if you unable to, if you do not practice presence with your staff what is the disadvantages, if you are not there for your staff to support and guide them, what can be the disadvantages of that?

Participant 10: Disadvantage that now the work now going to be done as supposed to be done. Things are not going to be done. I don’t know nurses are the people that now just have to look at them like, following them up. Just to make sure that the work now is being done, you understand. Disadvantage of not being there conflict, is then and it would be a supervisor or a matron that is not bias, you understand not going according to favors love them all, treat them all equally. If you treat everyone equally then you will be a bias and then you don’t gossip about your staff, your staff will trust you, that now you don’t gossip about them. If somebody is been gossiping about you I don’t think that you are going to trust him, no you are not. Are you even if you are having a problem are you going to help her, no, I know even if I’m having a problem, my matron will never help me but if you know that now even I can talk to my matron, you’ll be there for me. Even you ask them favors to do whatever they will do, to please you because they know that now you are there for them. If you are not there for them they are not going to be there for you also. Even if you are not off on the weekend they will make sure that now every equipment can’t go out of the department without not being written on the borrowing book, they will take the responsibility. But if you are not there for them they can sabotage you its how I see about the nurses. Treat them equally, that is the only thing, have meetings, let them come with decision making on your meetings, you understand. You can write the agenda there if somebody wants to talk about something to be rectified, give him or her a platform, so that that thing must be discussed and be rectified. Decision making is very important, hear from them even you can hear that but this one doesn’t go according to what I planned but you don’t say you are wrong you say to them what about doing this not this one because this one will help us not doing this. You don’t say the person is so, you don’t say the person is stupid no one is stupid but you advice the person to make the right decision, involve him during the meeting monthly even if don’t have monthly, I usually do quarterly meeting. And if anything that you go for any training as a operational manager on your whatsapp group like said before we are having whatapp group give them the report back, what has been said there, encourage them to read our SOP’s to know our policies. Teach them for each and every equipment that is in the department, bring the REP’s from outside for each and every equipment. There’s no use that now you bring the equipment but the staff there they don’t know how to use them. A simple glucometer if I give it to you for the patient you don’t know how to operate it are you going to know, no you don’t. but if somebody show to you the REP that is having that giving teaching them, they know how to use the equipment correctly. There will never be an adverse events or any incidents occur in our patients unnecessary. Because this patient know how to use the equipment they can see if the equipment is not working, they can report equipment, the equipment can be fixed in time, then it can be replaced in time and then continuous nursing care of the patient is in progress.

Researcher: that’s true. Matron. Thank you thank you very much, we had such a lovely conversation, look at the time. We’ve been almost 50 min together. Thank you very much I really really appreciate your insights. And everything of the best you doing a great job.

Participant 10: thank you very much also just to have a turn also to chat to you really to ask and also to its nice to

Female; Age-55 yrs; 3 yrs unit manager

**Participant 11:**

Researcher: Good morning, sir. Thank you very much for sparing your time, affording me this opportunity to ask you a few questions, okay. My name is Bernardine Smith and I am a master’s student at the North West University. I’m going to record the session, is it okay with you.

Participant 11: Yes, it’s okay with me.

Researcher: Thank you very much. There’s no right or wrong answer. What counts is your perspective of things of how you experience it and what you believe it is all about, okay.

Participant 11: Okay.

Researcher: I want to ask you one more favor. It’s my first time doing research, so if I maybe couldn’t get to answer all the questions, may I come back for follow-up questions?

Participant 11: Yes, you can come back.

Researcher: Okay, thank you very much. So my first question is what do you think are the current presence practices of unit managers in the hospital?

Participant 11: The current practices of the unit managers at the current state of time. Ahhh, you working as a unit manager and sometimes as a floor nurse due to the shortage of staff. And then as a manager sometimes there are so many things that come your way that you have to deal with it with them, like absenteeism, the management of sick leave of the personnel. And then the shortage of staff, mxm, sorry for that not staff the shortage of resources that we have to use for the sake of the patients.

Researcher: Okay. So when we talk about presence, how will you define, what is your understanding about presence? How will you define presence?

Participant 11: According to my understanding, presence mean at the present time, now.

Researcher: Okay, so how can you please explain to me how do you as a unit manager, I’m specifically talking about you now, how do you practice your presence in your unit? Meaning, how are you there physically, mentally, psychologically present for your patients in the unit?

Participant 11: Ja, I my patients I give them holistic nursing care. That simply means I care for them wholly. And then I see that I attend to their problems, if they are having problems and then I advocate for them for my patients during the rounds when the multi-disciplinary team is there. And then again I make sure that my patients are safe in the unit. And then I also make the infection control, infection prevention and control adhered to in order for my patient to be free from the infections, I think does it?

Researcher: Yes, thank you very much. So then how do you as a unit manager practice presence when it comes to your staff? Being there for them physically, mentally, psychologically, being there for your staff?

Participant 11: For my staff, I support them with their social problems, and then their emotional status. And then I also make sure that I help them develop themselves that simply means if anybody needs to go to school I support and motivate for the person to can go on for further study.

Researcher: Okay thank you very much. As a unit manager I would like to know how do you practice that relational care when it comes to your subordinates? How do you build a relationship, a professional relationship with your subordinates, with your staff?

Participant 11: Ja, the relationship, the relation I’m building with my staff, listen to their concerns and then if maybe I see they are not on the line I discipline them and then I try to motivate them to do what they are suppose to do.

Researcher: Okay, thank you. How do you practice that human connectedness with your subordinates?

Participant 11: Human connectedness to my staff, sometimes we hold the small anynah, the small parties together and go out to be together in our free time so that we can have time to know each other well even outside our workplace.

Researcher: Okay, okay, thank you very much. As a unit manager I know your role is very big. You’ve got a lot of duties that you need to do like your administrative duties and meetings and all the things you have to do. Does it impact you being present in the in your unit?

Participant 11: Sometimes it impacts me being in the unit because if I have to go away then you’ll find that maybe is one professional nurse and if I go and attend the meeting the professional nurse that is on duty is going to struggle to can handle the patients alone. So the other thing is prioritize but then if I see the ward is covered then I attend to such.

Researcher: Okay, thank you very much. If your staff, say for instance your staff do not practice presence, meaning that they not there in totality, nursing the patients in totality, if they don’t do that, what will be the disadvantages?

Participant 11: If they don’t do that the patients they are going to stay long in the hospital. And then most probably the others that are fragile are gonna develop the pressure sores of which the pressure sores are very, how can I say it, very difficult to can treat so and then the pressure sore are the thing that make the people to sue the hospital most. So I try by all means to talk to my staff to take care of the patients. They must know their reason here, they are not here to come sit or make friends they are here for the patients to care for patients in totality.

Researcher: Okay, so that is the disadvantages, what will the advantages be if your staff practice presence?

Participant 11: If my staff practice presence the advantages is that the stay of the patients in the hospital will be short and then one other thing they would have too much burden of work if they do what they are supposed to do.

Researcher: Okay, thank you very much.

Participant 11: Pleasure.

Researcher: What will be then the advantages if you as a unit manager practice your presence in the unit?

Participant 11: As a unit manager if I practice my presence in the unit, I think the department will be run smoothly without having any problems because I’ll be there and then overseeing that the people are doing what they are supposed to do for the patients.

Researcher: Okay, then what will be the disadvantages if you do not practice presence?

Participant 11: If I don’t practice presence the disadvantages is that there will be no smooth running of the department, there’s gonna be chaotic, and then its gonna, there’s gonna be lot of complaints that the patients are not cared for. And then even that will lead to the long stay of the patients.

Researcher: Okay, thank you. Is there anything that you think I should ask or anything that you would like to add to this conversation?

Participant 11: No thanks I’m fine.

Researcher: Are you fine, okay. Tell me how do you when your staff going through challenges, how do you support them?

Participant 11: If my staff going through challenges, for example if I see the person most of the time is off duty ant then I sit with the person and ask what could be the problems and then try to help, but then if I fail to can help I refer my staff to EAP, to can get a better help.

Researcher: Okay, so you also mentioned earlier on shortage of resources, how does that impact you as a unit manager when you want to practice your presence in your unit?

Participant 11: the shortage of resources impacts my presence because the patient will not be cared for as we are supposed to, so we’ll be improvising, improvising and then at end when the families came here then they gonna lock the complaints?

Researcher: So tell me when you are faced with such challenges, shortages of staff and shortage of resources, how and where do you get your support from?

Participant 11: If I have a challenge like that I get my support from my assistant manager but with the resources I ask from the other departments.

Researcher: Okay

Participant 11: And then staff sometimes, myself I ask the one’s that are off to can come and help.

Researcher: Okay. So thank you very much, very short and very sweet interview, I really appreciate taking your time to answer a few questions. Thank you very much, sir.

Participant 11: Thank you, you are welcome.

Researcher: Thank you.

Male; Age-53; 1 yr 7 months

**Participant 12:**

Researcher: Good morning matron, once again, thank you very much taking your time to answer a few questions, I really appreciate it. My name is Bernardine Smith and I am a master’s student at the North West University.

Participant 12: Okay

Researcher: Thank you. There’s no right or wrong answer, all I’m interested in is your perspective of the topic as we discussed the topic earlier on. If you have any questions afterwards, you can feel free to contact me or if you want to add anything, you can feel free to contact me. Is it fine with you if I record the session?

Participant 12: It’s fine with me

Researcher: Thank you. If I need follow up questions will you agree if I maybe can come back for one or two should it be needed.

Participant 12: Yes.

Researcher: Thank you very much. So let’s start with the questions. My first question is, what do you thing are the present practices of the unit managers in the hospital?

Participant 12: Practice like more or less.

Researcher: Presence like we discussed what presence is being there for your staff and the patients holistically, so what are the current practices?

Participant 12: I don’t know whether I understand the question, because the presence practices like it would differ from department to department. So I don’t know whether you are talking about the practice of when I’m arriving on duty what is it what we do, because I think it will differ from department as I’ve just said. So, in the morning akiri rhona in our department, that’s were we do the role call and previously, the previous day in the afternoon, the person who’s in charge will arrive on duty, will be doing, I mean the person in charge in the afternoon, like the sister that we left in there neh, she will do the allocation for us for the next morning because it’s not easy for us to do it in the morning, it’s the theatre where we having how many theatres 15 theatre’s. So, in the morning there’s not enough time for us to do the allocation in the morning, we will assess what has been done yesterday and then we re-allocate if it’s necessary neh. So, as they are coming in as we are doing our role call, our staff neh, others will be phoning that we are not coming, so then from there we have to re-allocate to cover the gap that is left by the one who’s not coming. So, you can imagine each and every theatre needs two professional nurses and two nurses. So, if it happens in the morning most of the time, sometime, maybe one nurse is not coming and then depending on the cases that they have, so the team should be four, you have to check where can you take who to close the gap. If that the other nurse, maybe they are having maybe two cases and then this one the are having five then you have to remove the one with the lessor cases to the more cases. And then sometimes in the morning you’ll find that there are some problems that we need to attend to of the staff itself neh, maybe someone is coming to work with emotionally stressed up, you also have to attend to that. So, you as an OPM on your side neh, like this department as I’ve said is so huge unfortunately with us there are doctors that has to come in the office with complaints that we have to attend to, as we are busy attending this one another one comes in so it’s a mess in the morning. So, it goes on like that until it comes to a level of calmness whereby all the theatre have commenced with their operations. So, I’m not sure whether I’m getting to your answer.

Researcher: Yes, yes are you done?

Participant 12: So far?

Researcher: So far, okay. Thank you. No, no its fine. So you mentioned something there, about some of the staff might come with did you say emotional stress?

Participant 12: Like, you know what they will come with their problems neh, you can imagine maybe it’s a problem that needs my attention immediately or sometimes I’m unable to attend to her immediately then I will just say okay come later when it’s quiet neh. So, you find that others, you don’t know how emotionally the person is heard but in the mean time on my side I have to start to make the environment of the theatre to be calm so that every body is covered, all the patients are wheeled in on time, no I must avoid the delay in the department. So then when I’m okay then I will call the person, now we can sit down because attending to a the emotionally disturbed person it takes a lot of time because you have to understand, you have to do the root cause analysis what was the problem, what happened you understand until the person is ready to can function, it depends on the problem of the day.

Researcher: Thank you matron. Can you tell me what do you understand by the word presence?

Participant 12: The presence means, okay on my side I understand it as I must be there all the time when the staff needs me, when there is something about my patient, I need to be there for that patient if someone is coming to report about the patient, akiri, you’ll find that maybe other patient arrives in our department but maybe you find something wrong, I need to attend to that. That’s where my presence comes in. then should I phone the matron of the other ward to solve the problem of that patient or should I solve it with the doctor, you understand or should I solve it with the team that are going to work with that patient, that’s where my presence comes in. Firstly it was my presence to the staff, presents to the patients, presence to the whole department, present with everyone who’s coming to me with the help need, am I able to attend to help those people with what they are coming to me with, its where my presence is.

Researcher: Thank you very much. So tell me being a unit manager having your leadership role and your leadership functions okay, how does that affect you being present?

Participant 12: Orite, since I said I have a year and six months, you can image how overwhelmed I am neh. There are duties that I still have to learn, for me I have to attend to the rest of the department as it is, I have to attend meetings neh, it is so so overwhelming for me but at some stage fortunately with me I’m a very, very, calm person, even if I see that I can’t manage, I’m a very, very, calm person, I wouldn’t even show you that now I’m so much overwhelmed. I’ll take them one by one, one by one until I finish what I’m supposed to be doing for that day. Though when I’m walking I can feel that joh this is heavy but even if someone can come into the office and shouting and doing what I just keep quiet look at that person in the eyes, just hear what the person is saying and understand, then I attend to that thing, even though something is still waiting for me I just prioritize okay let me help this person and then I’ll go to the next one, I’ll go to the next one one by one, my calmness really helps me a lot only when that I’m home that it will hit me. I will start by thinking joh maybe if I should have known that one and that one and that one at least you understand, the only thing I’m fighting myself with is my calmness, that all.

Researcher: So, your role how does it impact you being there for your staff?

Participant 12: Funny enough if I have to tell you neh, because there is something that I have realized from the staff side of it, as I’m saying my calmness fight for me. So, you can understand the staff neh, they don’t want to be shouted at neh, so they will always come and peep and look in the office and if they don’t see me there, others they just go back and if you ask them why didn’t you go to the office and then there was someone, they will just say no, we needed you. That’s why I’m saying other people need someone who’s calm who can manage something.

Researcher: Okay, so tell me how do you as a unit manager practice that relational care to your subordinates?

Participant 12: That relationship neh as I’m saying building up the relationship, as I’m saying, my calmness fight for me. So, the way I’m calm it allows people to come to me and then it allows some of them to divulge some of their information that they don’t want anyone to help with. And sometimes what I do, I’ll just do my rounds around just to show them I care for them or I’ll just go and sit down with them and just have a chat with them. At some stage when they see me, they will just say matron, there’s matron coming and I’ll just say don’t worry I’m not here with any delegation, I’m just here to visit and to hear from your side how are you feeling and what, what. That’s how I manage my relationship with them.

Researcher: Okay thank you matron. How do you as a unit manager practice that human connectedness, how do you connect with your sub ordinates?

Participant 12: From my side I don’t think it’s something that I practice, it’s something that is in me, I don’t practice it, it just happens. And then, at some stage even if someone has done wrong or offended me, I usually take some time, what can I say, to wake it in me. And then after some time then when I meet the person, they would sometimes think that I’m cross for them no, with them, but at some stage they would just hear me making jokes with them, okay and then making them understand okay that was work related thing now we are here let us forget about that one, it was supposed to be done I was supposed to correct you, you were wrong or I was wrong so its easy for me to ask forgiveness when I’ve wronged someone, so if I see the person can’t ask forgiveness from me then I’ll make that person to feel comfortable to feel like I’m not taking grudges, so that’s how I do it.

Researcher: Okay, thank you. Tell me if your sub ordinates having challenges how do you provide the support and guidance to them?

Participant 12: It depends from challenge to challenge, neh. So if something it’s something that I will advice a person with. I will sit down and I’ll make a person understand that I’m gonna give her the chance to make me understand what is the challenge and then every time as we are talking I’m making the person to trust me neh, then I’ll even have some examples that I will mention as we are talking before she even mention some other things. I will just make an example that this and that and this and that so that she feels, you understand, trusting me. And then after the person has told me with whatever is challenging I’ll tell the person you know what neh I’m gonna advice you but you are the one whether you take my advice or not or you assess the advice and then you keep what is what will work for you, so most of the time I will be like as I look at you neh sometimes they gift that I have I can see when you are going through something, you understand, and then I will come indirectly to you, maybe talking to you you know what I can see whatever, whatever and then you’ll find that the person is feeling comfortable and talking and talking and talking it’s then that I will say okay I’ve seen it neh but now I’m gonna advice you, the choice is yours. That’s the relationship I’m trying to I’m building with them.

Researcher: Okay, so tell me, how do you as a unit manager with your patients, how do you, if you can give me a few examples, how do you practice your presence in your theatre with your patients?

Participant 12: Unfortunately I’m not scrubbing in the theatre, but as our patients are lying here neh, that’s where maybe you can go and greed your patient, you understand and then just to make the patient you know what they didn’t forget about you as you are lying here, so we have so many theatres but don’t worry if you see someone arriving after you and they are taking that person you are not going in the same theatre that’s the only time that I can have the chat with my patient, be present for them, that’s the only time otherwise they are going inside that theatre, so I’m not there and I’m not scrubbing unfortunately for now.

Researcher: Okay, thank you. So if you are not there for your patients in that manner what will be the disadvantage of it?

Participant 12: The disadvantage is that for an example myself as a patient I would prefer neh to be informed so I think the disadvantage is that if they are not informed for example what I’ve just said that if they are lying there, they need to be informed, you are lying here you are waiting to be collected by the people who are going to help you with the different theatres you understand, so feel free if you want to sleep, you can sleep. The disadvantage, the anxiety with our patients they are lying there not knowing. I’m being collected from my ward but I’m lying here, you understand, so it’s the anxiety that I think that can go through. And then others with our cultures akiri we have different believes, you even call, you know what you bring different ideas into you as a patient, thinking that maybe my ancestors doesn’t want me to be here why others are going whereas I’m lying here you understand. I think the disadvantage of not knowing why are you lying there not going inside also it can have an impact on our patients.

Researcher: Okay, and if you practice your presence like when you go and talk to them and inform them what is the advantage of that?

Participant 12: To make them feel relaxed that which ever way I’ll be taken into theatre, I will be operated that’s it, the most important thing is for our patients to know that is the most important thing, I mean the advantage of knowing is you are calming them, you are making them relaxed, you are making them believe that whichever way I’ll be done today.

Researcher: Thank you so much. We spoken now about the advantages and disadvantages of the unit manager in your position, so then what will be the advantages if the, if your subordinates, the nurses, practice their presence with the patients in theatre?

Participant 12: All right, the advantage or the disadvantage?

Researcher: Advantage first?

Participant 12: Okay the advantage will be clear, akiri now, as they are lying here the environment in discovery is different neh, when they enter the theatre its another different environment you see big lights and what what neh, so another thing for them to be present for their patient is to make them understand what is the inside of the theatre immediately before they open the door, before the they wheel the patient in, they must make the patient that now we are entering where you gonna be operated but the environment here is different, you need to explain to them, the theatre might be very cold, you might see different huge light you understand and then this and this is gonna happen especially when during the induction they should know that some what is gonna happen of which sometimes other patients when their anesthetist went to do the pre-op they do explain what is happening inside the theatre that we gonna put a mask and a what what but now here its happening in real, yesterday it was just the explanation but now its reality is here you are now entering the theatre. I’m sure the anesthetist who visited you yesterday this and this, you have to explain everything that is gonna happen before you wheel in. immediately when you enter that whatever you explained the patient and then they see everything that you have explained I think from my side they also become relaxed because they are now knowing what is, what they are entering, which room what kind of a room they are entering in now.

Researcher: Okay and if the nurses do not practice their presence what will be the disadvantages?

Participant 12: The disadvantage is the anxiety again. Then you can imagine you as a patient you become afraid because now you don’t even now whats gonna happen with you. What can I say now, so you know the disadvantage again once you shrink akiri so your veins everything constricts, we struggle when we put up the drip akiri the veins are now constricted you understand so its more and more pricking, that’s lot of disadvantages that is happening inside, it’s anxiety, it’s struggling with putting up drips, and relaxation they will be shivering you understand.

Researcher: And vitals.

Participant 12: So the vital signs will be dropping will be low akiri at that time .

Researcher: Okay, thank you very much matron. We’ve spoken about challenges, how you support your staff, how do you get your support when you are faced with challenges?

Participant 12: For support I have a supervisor neh, I have my colleagues, of which if I’m been challenged I will come to them and discuss the challenge with them, guys I’m going through this and this and this or this has just happened how do I tackle it if it’s work related. Then if it’s personally related then I will it depends to me is it something that I share with my colleagues or does it need me to share it with my supervisor, one way or another I need to share it with someone else, that’s how I’m doing it.

Researcher: So you do get the necessary support that you need.

Participant 12: Yes I do get it from my colleagues and from my supervisor or akiri in a workplace there are people that you can choose whereby you can be feel comfortable to share some other stuff with and then they can advice you, like I’m doing to others, they can advice you, they can comfort you.

Researcher: So I’ve just said challenges what type of challenges are you been faced with in your position.

Participant 12: In your position, starting from the patient’s side, you’ll find that maybe patient is not prepared well. And then secondly, maybe the consent forms are not signed and what what neh. And secondly you find maybe the challenge of the stock, challenge of the complaints around neh from the doctors, complaints from the subordinates, and sometimes not meeting my deadline with my admin work, is another challenge and then running to the meetings again whereas I’m thinking to finishing up this but I have to run somewhere that’s the challenges on my side that I’m faced with.

Researcher: Okay, thank you very much matron and lastly if you can tell me how do you tackle those challenges personally?

Participant 12: Personally, as I’ve said I’m a very calm person that’s how I challenge it, that’s my stick to fight. So I become very calm even if, maybe let me say there is a stock complain I’ll just be calm and see what I can do either I borrow from one hospital and what what or I borrow from the ward or I do something but sometimes what I do if I’m unable to have a plan neh I usually say to the person who came with the complain to me I’ll come to you later, you can go now so that the person does not see that you know what I don’t even know do I go to that corner or to the other one, so I’ll just say I heard you, you can go I’ll come back to you in the meantime as I’m doing my plans.

Researcher: Thank you very much matron I really appreciate you taking up your time to answer a few questions.

Participant 12: I also hope that I helped you but it’s from me, that’s what happens. It’s form me. Its not something that we are doing all of us. Everything that I’ve told you is what I do nna to survive my days.

Researcher: No, you definitely did help me and I appreciate, thank you very much

Participant 12: Thank you so much.

Female; Age-54 yrs; 1 yr 6 months
